# An agentic framework for autonomous scientific discovery in cancer pathology

**DOI:** 10.1038/s41591-026-04357-y

**Published:** 2026-04-29

**Authors:** Florian Trost, Bide Zhang, Ines Aring, Marcus Bauer, Lennert Glamann, Michael Wessolly, Kyra Johnson, Heike Göbel, Tristan Lerbs, Taban Sangenne, Peter Herrmann, Fabian Mairinger, Christopher Kopp, Sebastian Michels, Anna Rasokat, Matthias Heldwein, Steffen Wagner, Birgid Schömig-Markiefka, Jürgen Wolf, Sylvia Hartmann, Claudia Wickenhauser, Andrey Bychkov, Jens Peter Klussmann, Alexander Quaas, Reinhard Buettner, Yuri Tolkach

**Affiliations:** 1 Institute of Pathology, University Hospital Cologne, Medical Faculty, University of Cologne, Cologne, Germany; 2 Institute of Pathology, University Hospital Halle, Halle, Germany; 3 Institute of Pathology, University Hospital Essen, Essen, Germany; 4 Department of Otorhinolaryngology, Head and Neck Surgery, Faculty of Medicine, University of Cologne, Cologne, Germany; 5 Clinic I of Internal Medicine, Center for Integrated Oncology, University Hospital Cologne, Cologne, Germany; 6 Department of Cardiothoracic Surgery, University Hospital Cologne, Cologne, Germany; 7 Department of Otorhinolaryngology, Head and Neck Surgery, University of Giessen, Giessen, Germany; 8 Kameda Medical Center, Kamogawa, and Nagasaki University, Nagasaki, Japan

**Keywords:** Cancer, Biomarkers, Evolution

## Abstract

Artificial intelligence has advanced cancer pathology, but many systems still depend on hand-crafted features, are hard to explain and rely on fragmented workflows. We introduce SPARK (System of Pathology Agents for Research and Knowledge), a foundational agentic artificial intelligence approach that uses language as a universal interface to autonomously generate biologically driven concepts for tumor analysis. SPARK turns biological ideas into analytical tools and works directly with complex pathology data without extra model training. We evaluated SPARK across 18 patient cohorts spanning five cancer types (lung adenocarcinoma, lung squamous cell carcinoma, colorectal cancer, breast cancer and oropharyngeal squamous cell carcinoma) and more than 5,400 patients with available histopathology images and clinical/follow-up information, in both prognostic and predictive settings and on a well characterized spatial biology breast cancer dataset (patient *n* = 625). We found that SPARK produced clinically and biologically relevant concepts correlated with prognosis, known pathological variables and predictive biomarkers, including patterns of tumor progression and temporal change inferred from static images. A dedicated module allows for human interaction with SPARK. Further prospective validation is needed to evaluate the clinical utility of the tools created by SPARK. All code, parameters and results are openly released to help researchers and clinicians improve diagnostic precision and deepen tumor biology insights.

## Abstract (web summary)

Evaluated across 18 multicancer cohorts, the agentic artificial intelligence workflow SPARK uses language as a universal interface to autonomously generate biological ideas.

## Main

Artificial intelligence (AI) is rapidly transforming pathology by helping to automate and standardize diagnostic tasks^1^. Beyond improving efficiency, AI tools can extract new types of information from routine histological slides, especially in the oncology domain^2–4^.

There are several main avenues how AI can analyze hematoxylin and eosin (H&E)-stained images of tumors. One approach, called tissue segmentation, divides the slide into different tissue types (for example, tumor, stroma, necrosis or lymphoid areas). This allows pathologists to quantify broad structural patterns (for example, tumor-to-stroma ratio) that have been linked to clinical outcomes^4,5^. A more detailed strategy uses cell-level detection and classification, where AI identifies individual cells and their interactions within the tumor microenvironment (TME)^6–12^.

The newest generation of AI models, known as foundation models, are being trained on vast collections of histopathology images to learn general patterns of tissue organization. Once developed, they can be adapted (‘fine-tuned’) to perform a wide range of diagnostic or predictive tasks^13–15^. However, foundation models are not accurate by themselves—their performance depends on how well they are adapted to specific datasets and clinical questions. Other challenges are limited interpretability (‘black box’ behavior), the need to aggregate information from thousands of small image regions per case and potential pretraining-related biases^16^.

Recently, new classes of AI algorithms have emerged that aim to bridge these gaps. Large language models (LLMs) have shown remarkable ability in text-based reasoning but cannot be directly applied to pathology images^17^. Even vision-language models (VLMs), which combine image and text understanding, currently struggle to provide reliable answers to even simple quantitative or reasoning-based questions^18–21^.

A promising direction is the development of agentic AI systems, where several specialized algorithms (‘agents’) are connected into a coordinated workflow. Each agent performs a specific function, and together they can tackle more complex analytical tasks than any single model alone^22,23^. Evidence from small cytoplasmic RNA sequencing analysis^24^ and foundation-model-based pathology applications^25–28^ demonstrates advantages of multiagent systems. Nonetheless, current approaches remain constrained in flexible, unconstrained reasoning. This paradigm could therefore address several limitations observed when LLMs and VLMs operate alone.

**Fig. 1:**
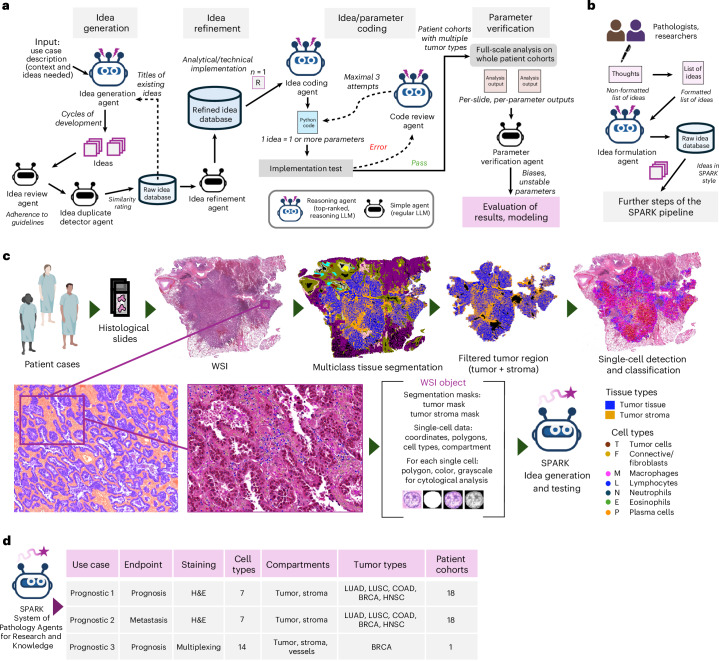
SPARK overview, data structure and study design. **a**, Simplified overview of SPARK, a reasoning pathology ‘brain’ that autonomously generates biologically grounded ideas and implements them for testing in large cohorts of patients. SPARK consists of four linked parts: idea generation, idea refinement, idea/parameter coding and parameter verification. **b**, One additional SPARK module allows human-initiated concept exploration. Human analysts provide their ideas in minimally structured free-text form (for example, ‘1. Number of lymphocytes close to tumor cells. 2. Density of macrophages in tumor stroma’.). The module prepares them to include in the main SPARK pipeline. **c**, Structure of the data used as input for SPARK. Routine digitized histological WSIs of tumors are preprocessed by a quality control algorithm and organ-specific multiclass tissue segmentation to identify tumor regions and precisely map epithelial and stromal tumor compartments, followed by an algorithm that detects single-cell types. Resulting WSI object is used as input for SPARK in evaluation phase. **d**, Study design and use cases with translational relevance and cohorts (clinicopathological characteristics in Supplementary Table 1). In use cases 1 and 2, SPARK is tasked for generation of biological concepts and extraction of prognostic information from routine H&E slides; in use case 3, from spatial biology data. LUAD cohorts, TCGA, PLCO, NLST, UKK, UKE; LUSC cohorts, TCGA, PLCO, NLST, UKK, UKE; COAD cohorts, TCGA, PLCO, HAL, UKK; BRCA cohorts, TCGA, UKK; HNSC cohort, UKK. T, tumor cells; F, connective/fibroblasts; M, macrophages; L, lymphocytes; N, neutrophils; E, eosinophils; P, plasma cells. Illustration in **c** created in BioRender; Tolkach, I. https://biorender.com/jol566b (2026).

Here we introduce a new paradigm for image analysis in cancer pathology, termed SPARK (System of Pathology Agents for Research and Knowledge) (Fig. 1a). SPARK functions as a pathology ‘brain’—an interconnected system of AI agents that use language as a universal interface to autonomously reason, generate and implement biologically meaningful hypotheses as analytical tools—all without additional model training (Fig. 1c).

We validated SPARK across 18 patient cohorts representing five different cancer types in both prognostic and predictive settings, as well as in a spatial biology application involving highly complex datasets. SPARK provided new biological insights, revealing how tumor evolution and mechanisms of increased aggressiveness can be inferred from standard histological images.

## Results

### Overview of SPARK

In essence, SPARK functions as a digital pathology ‘brain’ that is designed to ‘think’ about tumor biology, autonomously generate strategies for tissue analysis and execute analytical approaches for large-scale studies in patient cohorts.

SPARK has a flexible, modular design with four linked parts: idea generation, idea refinement, idea/parameter coding and parameter verification (Fig. 1a; for technical details, see Methods). Beyond autonomous discovery, an interactive interface lets clinicians and researchers work with SPARK directly, enabling rapid, code-free creation of new analytical parameters for exploratory biomarker discovery (Fig. 1b).

SPARK adapts to new tasks through reasoning and tool-building rather than retraining. It processes routine H&E whole-slide images (WSIs) by performing quality control, organ-tailored tissue segmentation to create detailed tumor maps and single-cell detection and labeling across seven major cell types (Fig. 1c).

In the following sections, we demonstrate how SPARK enables discovery of robust, clinically relevant tissue biomarkers across multiple applications selected for direct translational impact (Fig. 1d): (1) routine H&E-stained tumor sections across five tumor types and 18 patient cohorts (Supplementary Table 1), chosen because H&E is universal in practice—here SPARK yields prognostic and predictive biomarkers to improve risk stratification and therapy selection; (2) spatial biology and multiplexed imaging, selected because they resolve fine-grained cellular organization within the TME—here SPARK derives mechanism-aware markers that clarify tumor biology and inform prognosis and therapeutic targeting (for example, immuno-oncology agents); and (3) analyses of tumor change over time, prioritized to elucidate mechanisms of progression—here SPARK infers dynamics from static images to guide targeted molecular studies of natural tumor evolution with direct implications for patient outcomes.

### Use cases 1 and 2, idea generation

**Fig. 2:**
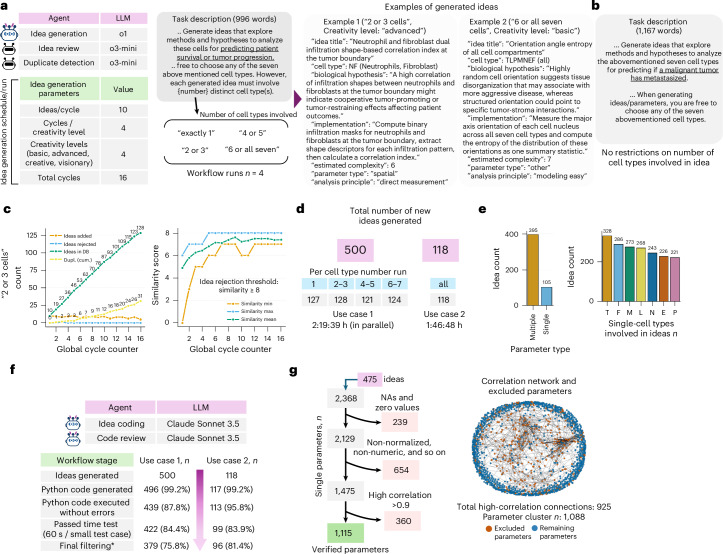
SPARK idea generation and implementation for use cases 1 and 2. **a**, Use case 1: LLM agents, idea generation scheduling, task description and examples of generated ideas (full list of ideas is available in Supplementary Table 2 and in the full idea databases; Supplementary Information). The number of cell types involved in each generated idea during one run is used to make the idea generation process more structured and systematic. **b**, Task description for use case 2. **c**, Use case 1: statistics of idea generation process. Shown is one run of four, for ideas involving two or three different cell types (for other runs, including token consumption, see Extended Data Fig. 1a–e). Left: number of original ideas added to database, number of duplicates (already available in idea database) and rejected (due to guidelines violation) ideas during generation. Right: similarity scores (produced by LLM agent to identify duplicates) of newly generated ideas when compared to ideas already present in the database; note increasing trends in later cycles, indicating reduced capacity for original ideas. **d**, Use cases 1 and 2: numbers of ideas generated during all runs and time required for idea generation. **e**, Use case 1: most ideas involve analysis of multiple cell types at the same time. There is an excellent involvement of different cell types among generated ideas (more statistics in Extended Data Fig. 1f–h). **f**, Implementation of ideas in code. Each idea usually contains one or more measured parameters. There is high yield of ideas successfully implemented in Python code. *Final filtering involves computational efficiency. The parameters that, for example, require hours to analyze single slides were excluded (computationally inefficient parameters). **g**, Parameter verification. The statistical outputs generated were analyzed, and redundant parameters (highly correlated, effectively measuring similar features) were excluded, leaving 1,115 parameters for downstream analysis. The full list of verified parameters before and after decorrelation is provided in Supplementary Tables 3 and 4. Dupl. (cum), cumulative number of duplicate ideas; DB, idea database, NA, missing value.

**Extended Data Fig. 1:**
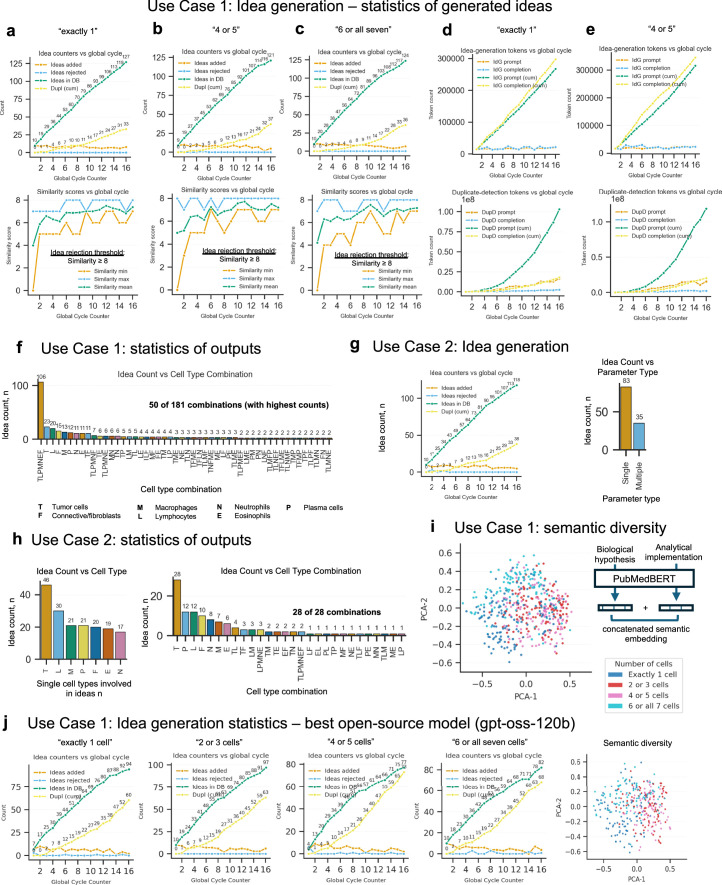
SPARK: Idea generation. **a-c:** Metrics of idea generation for Use Case 1 (runs with different amount of allowed cell types/idea), top - number of original, duplicate, and rejected ideas during the generation process, bottom - similarity scores of ideas during generation cycles, showing increasing scores in later cycles, indicating reduced capacity for original ideas because of saturation. **d-e**: Use Case 1: Token consumption for different parts of the idea generation pipelines, top – idea generation step, bottom – duplicate detection step. Shown are only runs with one cell types and 4 of 5 distinct cell types. **f:** Use case 1: statistics of output. Plot shows the number of ideas based on analysis of certain cell type combinations. **g,h:** Use case 2: idea generation and statistics of output. Plot shows the number of ideas based on analysis of certain cell type combinations. **h:** idea generation statistics for Use Case 2. **i:** Analysis of semantic diversity of ideas. Two semantic vectors are produced by PubMedBERT, one from biological hypothesis description and one from description of analytical implementation. These vectors are concatenated and processed by principal component analysis (PCA) and two dimensions. Significant spread of ideas (stemming from 4 different runs, corresponding to colors) can be observed. **j:** Idea generation statistics (same as in a-c) and semantic diversity for the best performing open-source model (gpt-oss-120b).

We used SPARK to propose prognostic biomarker concepts from routine H&E tumor slides. The task description of SPARK included the data available (Fig. 1c), and all formulations were tumor-type-agnostic (ideas should be applicable to all cancer types). To explore the space systematically, use case 1 fixed how many cell types could appear in each concept and ran four independent cycles (Fig. 2a). Use case 2 asked SPARK to identify tissue parameters linked to metastatic spread (Fig. 2b) without constraining the number of cell types. Within each cycle, we varied ‘creativity’ across four levels—from basic to visionary—to elicit both conventional and novel ideas. SPARK used the OpenAI o1 reasoning model (January–February 2025) as the Idea Generation Agent. We removed near-duplicates by similarity analysis; summary metrics (idea counts and similarity) for use case 1 are in Fig. 2c and Extended Data Fig. 1a–d. Examples of ideas appear in Fig. 2a, with the full idea set and parameter book in the Supplementary Information.

Each cycle of use case 1 took up to 2 h 19 min and yielded, in total, 500 unique ideas (121–128 per run; Fig. 2c,d). Most concepts (79%) involved multiple cell types, with balanced representation across major tissue compartments (Fig. 2e and Extended Data Fig. 1f).

This single, unrestricted run of use case 2 produced 118 additional ideas, with 70.3% focused on single-cell-type parameters (Fig. 2d and Extended Data Fig. 1g,h). Notably, this simpler setup still surfaced integrative, multi-cell-type ideas. Semantic analysis of generated ideas shows notable breadth of concepts (Extended Data Fig. 1i). We provide extensive tests of open-source LLMs (general and medical domain) for idea generation representing a viable alternative (Extended Data Fig. 1j and Supplementary Figs. 1 and 2). In systematic post hoc experiments, we were not able to show the unambiguous effects of creativity levels on idea yields or diversity in saturated idea generation outputs (Supplementary Figs. 3 and 4).

### Use cases 1 and 2, idea coding and verification

Based on preliminary benchmarking, we selected Claude Sonnet (Anthropic) as the coding agent. Across both use cases, 99.2% of proposed parameters compiled to executable code (Fig. 2f). After applying quality filters for efficiency and reproducibility (Methods), 379 ideas (75.8%) from use case 1 and 96 ideas (81.4%) from use case 2 were retained; most mapped to multiple analytical parameters (that is, a computable, numeric image parameter derived from an LLM-generated idea; measured on patches/regions; aggregated to the case level). Larger reasoning open-source models can be an excellent cost-free alternative for parameter coding (Supplementary Fig. 4e).

Use case 2 was an exploratory search for a single ‘magic-bullet’ parameter explaining metastatic behavior. Early correlations with pathological nodal (pN) stage (‘Results’) showed that no single parameter captured this complexity. We therefore reclassified all ideas as prognostic and combined them for downstream analyses.

Verification of the coded parameters (2,368 parameters from 475 ideas) yielded 1,115 nonredundant (decorrelated) parameters suitable for further analysis (Fig. 2g). Complete parameter definitions are provided in Supplementary Tables 2–4 and in an interactive, searchable HTML compendium.

### Correlation to key pathological variables and predictive biomarkers

**Fig. 3:**
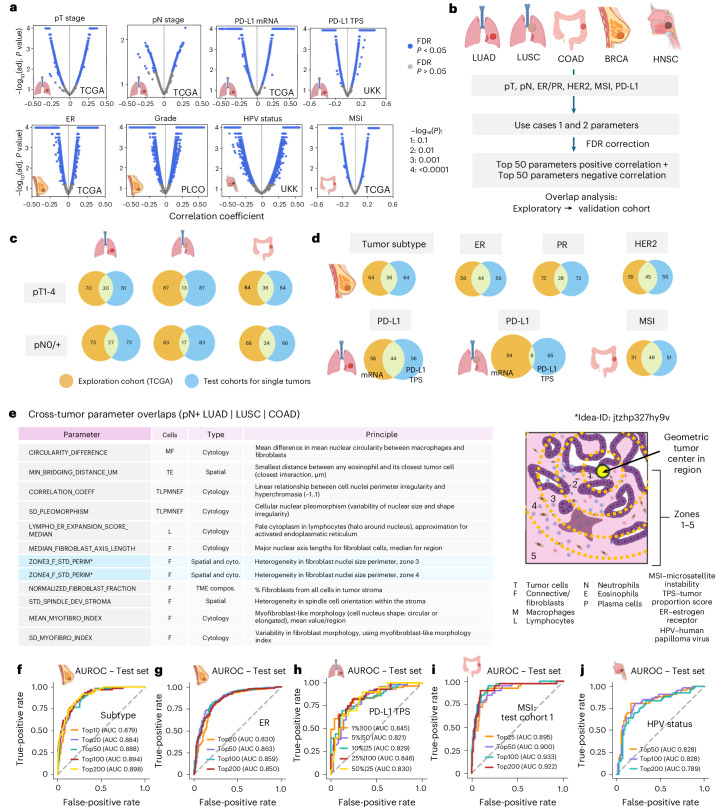
Correlation of SPARK-generated parameters with pathological variables and predictive biomarkers. **a**, Correlation of SPARK parameters with pathological variables and predictive biomarkers (*P* values are from Spearman correlation after FDR correction; two-sided). TCGA, PLCO and UKK are single cohorts analyzed for respective tumor type (full results in Extended Data Fig. 2a). **b**, Validation principle of detected parameters with high levels of correlation. For each tumor, we use two sets of identified correlated parameters: one for exploration cohort (always respective TCGA cohort) and one for test cohort (Methods). **c**,**d**, Analysis of overlaps in parameters with high correlation for exploration and test cohorts (other tumors in Supplementary Fig. 5b–d). In **c**, analysis for pT and pN stages are shown. In **d**, the tumor subtype of BRCA includes non-special type and invasive lobular carcinoma type. For PD-L1, mRNA expression of CD274 was used for the non-small-cell lung cancer TCGA cohorts and compared to parameters with high correlation to the TPS (immunohistochemistry; test cohorts UKK/UKE), respectively. **e**, Review of parameters with the highest correlations to pN+ status (in TCGA LUAD, TCGA LUSC and TCGA-COAD cohorts; corresponds to Supplementary Fig. 5d). The graphical abstract of one idea shown on the right side corresponds to parameters highlighted in blue (ZONE3_F_STD_PERIM and ZONE4_F_STD_PERIM) and represents one of the zonal concepts developed by SPARK. **f**–**j**, Accuracy of the trained models for predicting the status of pathological variables or predictive biomarkers: subtype/BRCA (**f**), estrogen receptor (ER)/BRCA (**g**), PD-L1/LUAD (**h**), MSI status/CRC (test cohort 1) (**i**), HPV status/HNSC (**j**) (XGBoost; full analysis in Extended Data Fig. 2d–q; feature importance analysis in Fig. 4a,b and Supplementary Fig. 5). All AUROC curves are for independent test datasets. Shown are models trained on different numbers of SPARK parameters with top correlation to the target variable. Top*X* means that the model includes *X* top features with positive and negative correlation (that is, Top20: 20 positive + 20 negative parameters). PD-L1 TPS (**h**) is predicted using dichotomization at different cut-offs (1%, 5%, 10%, 25% and 50%; full analysis in Extended Data Fig. 2g–j,m–q). For each cut-off, a separate model was trained. For MSI, training was on the TCGA-COAD dataset, test cohort 1 is COAD-UKK (**i**) and test cohort 2 is COAD-HAL (Extended Data Fig. 2e). adj., adjusted; PR, progesterone receptor. Illustrations created in BioRender: **a**–**d**,**f**–**j**, Tolkach, I. https://biorender.com/bab1y26 (2026); **e**, Tolkach, I. https://biorender.com/s16jj2n (2026).

**Extended Data Fig. 2:**
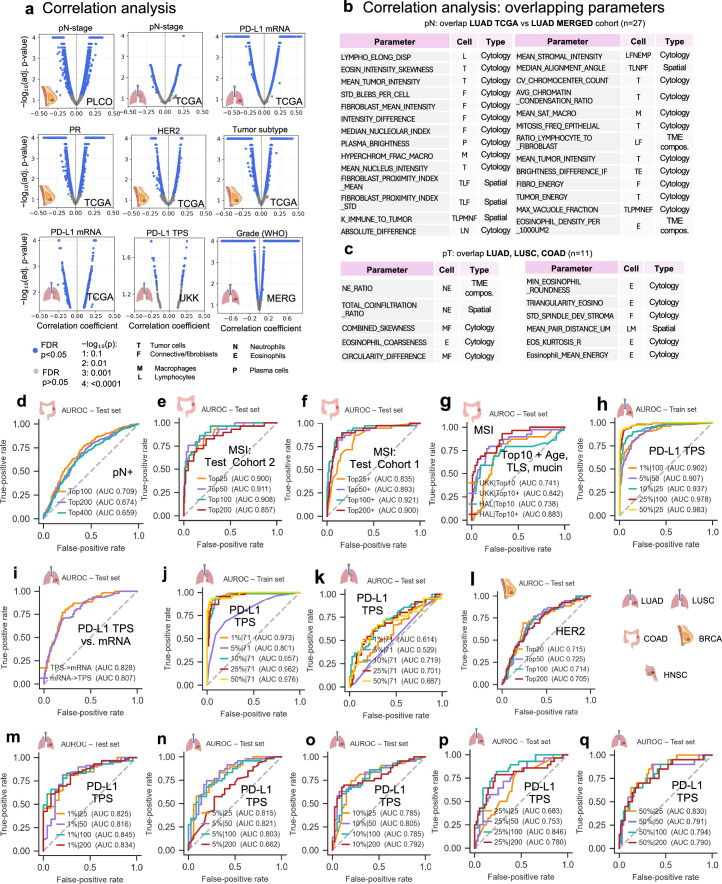
Correlation to pathological and predictive variables. **a:** Correlation of SPARK parameters with pathological variables and predictive biomarkers in tumor cohorts (Spearman correlation; after FDR correction; further analysis in Supplementary Fig. 5). p-values are from Spearman correlation after FDR correction (two-sided). **b:** Analysis of overlaps among parameters with high correlations to pN+ status. Cohorts: LUAD TCGA vs LUAD MERGED (test cohort). **c:** Analysis of overlaps among parameters with high correlations to pT status. Cohorts: LUAD TCGA vs LUSC TCGA vs COAD TCGA (corresponds to Supplementary Fig. 5c). **d-q**: Accuracy of the trained models (XGBoost-based models for tabular data). All area under receiver operating curve (AUROC) curves are for independent test datasets, except **h** (training set; to demonstrate learnability under different TPS positivity cut-offs). Shown are models trained on different numbers of SPARK parameters with top correlation to the target variable. TopX means that the model includes X top features with positive and negative correlation (that is, Top20 means 20 parameters with positive + 20 parameters with negative correlation, for a total of 40 parameters). **i:** training on the TCGA-LUAD dataset using mRNA expression and validation using the merged UKK/UKE dataset (mRNA 75th percentile → TPS 50%), and vice versa (TPS 5% → mRNA 75th percentile). **f-g:** prediction of MSI using Top parameters and selected clinical variables (Supplementary Fig. 5e). **m-q:** predictions using different cut-offs of PD-L1 TPS score positivity. Illustrations in **a,d**–**q** created in BioRender; Tolkach, I. https://biorender.com/bab1y26 (2026).

Many SPARK parameters track with core pathological variables across cancers (Fig. 3a; principles of case-level aggregation in Methods). They correlate with histologic grade in breast (BRCA) and lung adenocarcinoma (LUAD), breast tumor subtype and standard predictive biomarkers: estrogen receptor (ER)/progesterone receptor, human papilloma virus (HPV)/p16, microsatellite instability (MSI) and PD-L1 in both immunohistochemistry and mRNA expression (Fig. 3a and Extended Data Fig. 2a). Notably, PD-L1 status (tumor proportion score (TPS)) shows a strong signal in LUAD but not in lung squamous carcinoma (LUSC), indicating that H&E morphology carries cues about immune evasion in LUAD specifically (Fig. 3a and mRNA-level validation Extended Data Fig. 2a).

These correlations replicate in independent exploration and test cohorts for each tumor type (Fig. 3b–d). The top 100 significantly associated parameters show broad within-cancer overlap (Fig. 3c,d), especially for predictive biomarkers (Fig. 3d). We also observe shared signals across tumor types when considering the top 200 parameters (Extended Data Fig. 2b,c and Supplementary Fig. 2b,c). Full parameter and overlap lists are in Supplementary Tables 5 and 6.

Detailed review showed that parameters cover a diverse range of biologically meaningful concepts (Fig. 3e). Among parameters overlapping across LUAD, LUSC and colon adenocarcinoma (COAD), 10 of 12 involve fibroblasts, including their cellular and spatial patterns (Fig. 3e). Specifically, SPARK identified intratumoral zonal concepts that showed significant associations in both correlation and subsequent prognostic analyses (Fig. 3e). Notably, the parameters driving this finding originate primarily from cell analyses in zones 3–4, which likely represent the active tumor front and reflect how far the tumor can influence and activate its surrounding microenvironment. For pathologic T (pT) stage in the same tumors, 7 of 11 overlapping parameters highlight neutrophils or eosinophils (Extended Data Fig. 2c), foreshadowing their roles in tumor evolution discussed below.

### Prediction of biomarker status

Building on the correlations above, we asked whether SPARK parameters can predict biomarker status. We trained lightweight XGBoost models (≤5 epochs; Methods) on the exploration cohort and evaluated them on an independent test set, reporting area under receiver operating curve (AUROC; higher is better).

**Fig. 4:**
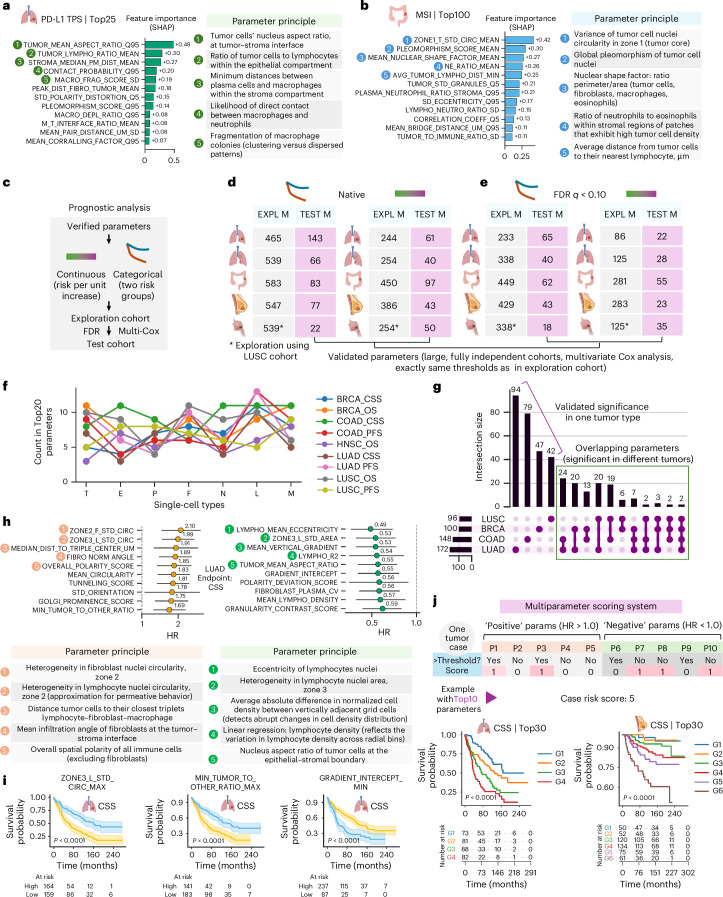
Analysis of predictive and prognostic value of SPARK parameters. **a**,**b**, SHAP analysis of feature importance for the models presented in Fig. 3h (**a**) and Fig.3i (**b**), including top parameter review (full results, Supplementary Fig. 5). **c**, Principle of prognostic analysis for SPARK parameters. **d**, Results of multivariate Cox analysis (numbers are parameters with prognostic value) without FDR correction in the exploration cohort (always TCGA cohort; ‘EXPL M’) and test cohorts (merged test cohorts for all tumor types other than BRCA and HNSC—only single test cohorts available; ‘TEST M’). Two types of analysis are presented: each parameter as a categorical (using the best cut-off) and a continuous variable (details in Supplementary Tables 8–17. **e**, Same analysis as in **d**, after applied FDR correction. **f**, Single-cell-type participation in Top20 independently validated parameters for different tumors and endpoints. **g**, Analysis of overlaps in significant parameters between different tumor types (non-adjusted version; FDR-adjusted version in Extended Data Fig. 3b). **h**, Top independently validated parameters with positive (left) and negative (right) significant prognostic associations for LUAD/cancer-specific survival (CSS; merged test cohort, patient *n* = 324); other tumors in Extended Data Fig. 3d–f and Supplementary Fig. 6. All values (central measure) are hazard ratios; error bars are 95% confidence intervals (CIs) from multivariate Cox analysis. Biological review of the five most significant parameters is provided. **i**, Examples of risk stratification via developed parameters (Kaplan–Meier curves with 95% CI and log-rank test *P* values) from **h** (more examples in Extended Data Fig. 6). **j**, Principle of integrative prognostic scoring involving multiple parameters. LUAD (independent merged test cohort; endpoint, CSS) and BRCA (independent test cohort; endpoint, CSS) are provided as examples using Top30 identified prognostic parameters. Risk stratification using four prognostic groups for LUAD (quantiles: Q25, Q50 and Q75 for scores as cut-offs; panel **m**) and six prognostic groups for BRCA (quantiles: Q10, Q25, Q50, Q75, Q90 as cut-offs) (full analysis in Extended Data Fig. 3g–n and Supplementary Figs. 7–9). HR, hazard ratio. Illustrations in **a**,**b**,**d**,**i**,**j** created in BioRender; Tolkach, I. https://biorender.com/bab1y26 (2026).

**Extended Data Fig. 3:**
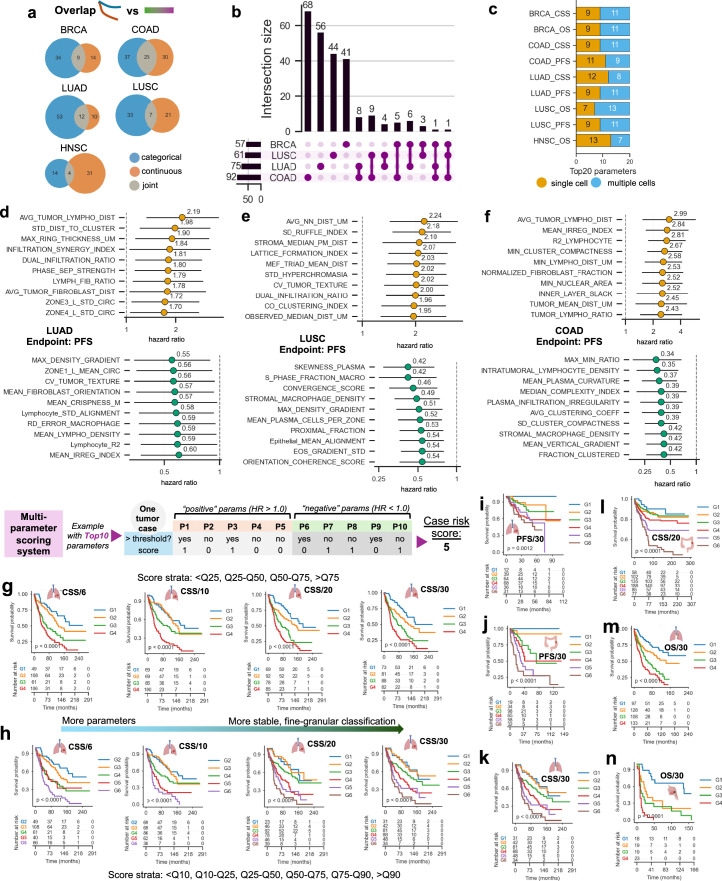
Prognostic value of SPARK parameters. **a**: Overlap between parameters studied as categorical and continuous variables, per tumor type (all results are from multivariate analysis in independent test cohorts). **b**: Analysis of overlaps in significant parameters between different tumor types (FDR-adjusted version of independent prognostic validation in test cohorts). **c**: Analysis of parameter structure (single vs. multiple cell types) for the Top20 parameters for different tumors and clinical endpoints. Roughly equal distribution can be seen for parameters analysing single and multiple cell types. **d-f**: Top parameters with positive (orange dots, hazard ratio/HR > 1.0) and negative (green dots, HR < 1.0) significant prognostic associations in different tumor types (additional results for other tumors and endpoints in Supplementary Fig. 6), from independent validation in test cohorts except COAD where exploration TCGA cohort is used (patients n; LUAD 251, LUSC 163, COAD 306). The central measures (orange and green points) are HR. Error bars are 95% confidence interval for HR. **g-h**: Influence of the number of Top parameters included in multi-parameter prognostic scoring (Top parameters n = 6, 10, 20, 30). Two strata are shown with four and six prognostic groups. Tumor type: Lung adenocarcinoma (LUAD); endpoint: cancer-specific survival (CSS). **i-n**: Further representative examples for different tumor types, prognostic endpoints and number of prognostic groups (Full analysis in Supplementary Fig. 7-9). All p-values in **g-n** are from log rank test. Illustrations in **i**–**n** created in BioRender; Tolkach, I. https://biorender.com/bab1y26 (2026).

In BRCA, models performed well for histologic subtype (AUROC 0.898) and ER status (AUROC 0.863) (Fig. 3f,g). HER2 status prediction revealed more difficult (maximum AUROC 0.725; Extended Data Fig. 2l). HPV/p16 matched state-of-the-art accuracy in head-and-neck squamous cell carcinoma (HNSC; AUROC 0.828; Fig. 3j)^29^. PD-L1 (TPS) was predicted at clinically meaningful levels in LUAD across therapeutic cut-offs (Fig. 4h; full results including mRNA-level validation in Extended Data Fig. 2h–l,m–q), but not in LUSC (maximum AUROC 0.719 at the 10% TPS cut-off; Extended Data Fig. 2j,k). MSI status reached top performance in two independent test cohorts (AUROC up to 0.933; Fig. 3I and Extended Data Fig. 3e). Adding clinicopathologic variables (age, tertiary lymphoid structures, mucin) did not improve models overall but meaningfully boosted accuracy when only the Top10 SPARK parameters were used (Extended Data Fig. 2f,g).

Model interpretation (Shapley additive explanations (SHAP)) highlighted biologically plausible drivers (Fig.4a,b; full analysis in Supplementary Fig. 5). For PD-L1 in LUAD, tumor cell nuclear shape at the tumor–stroma interface, number of lymphocytes in the intraepithelial compartment, as well as macrophage contacts to plasma cells and neutrophils and their spatial orientation were of major significance (Fig. 4a). Notably, a recent study employing advanced molecular profiling identified cell-type signatures predictive of immunotherapy response in non-small-cell lung cancer, involving tumor cell features as well as granulocytes, lymphocytes and macrophages^30^. Strikingly, these signatures show strong similarities to our findings derived from SPARK features, despite being obtained from routine H&E-stained sections.

### Prognostic analysis

Prediction of disease course is one of the important tasks in oncology. We asked whether SPARK-generated tissue parameters predict outcomes across five cancers (LUAD, LUSC, COAD, BRCA, HNSC) using 729 parameters decorrelated at prognostic risk stratification level and excluding parameters vulnerable to batch effects (Methods and Supplementary Table 7). The overall number of patients for all five tumor types was more than 5,400 (Supplementary Table 1). All patients underwent primary surgical treatment (without neoadjuvant therapy). Each parameter was tested two ways—as a continuous score and as a simple high/low marker—using multivariable Cox models in discovery cohorts (The Cancer Genome Atlas (TCGA) for each tumor), with confirmation in full independent test cohorts, and often multiple independent subcohorts per tumor type (Fig. 4c and Supplementary Table 1).

This analysis uncovered many parameters with prognostic value independent of clinical factors (Fig. 4d,e). The most informative cell types varied by tumor (Fig. 4f), with substantial overlaps of significant parameters among tumor types (Fig. 4g). Single-cell-type and multi-cell-type parameters contributed in roughly equal measure (Extended Data Fig. 3c).

Thus, in the LUAD tumor type, parameters identified in the exploratory TCGA cohort and successfully validated in the merged independent test cohort—comprising the international Prostate, Lung, Colorectal and Ovarian Cancer (PLCO), National Lung Screening Trial (NLST), University Hospital Cologne (UKK) and University Hospital Essen (UKE) subcohorts (Fig. 4h)—represent biologically meaningful features. These parameters capture multiple cellular components and dimensions of the TME, ranging from single-cell characteristics (such as nuclear shape at the stromal boundary, identified as part of tumor evasion properties; Fig. 4a) to higher-order TME organization, including polarity and spatial gradients of immune cells and fibroblasts. Notably, lymphocyte nuclear features—potentially reflecting permeative behavior or activation (that is, directed movement; nuclear circularity or eccentricity) or distinguishing between lymphocyte subtypes to some extent based on H&E staining, as suggested by recent studies^31^—emerge as among the important prognostic parameters.

**Extended Data Fig. 4:**
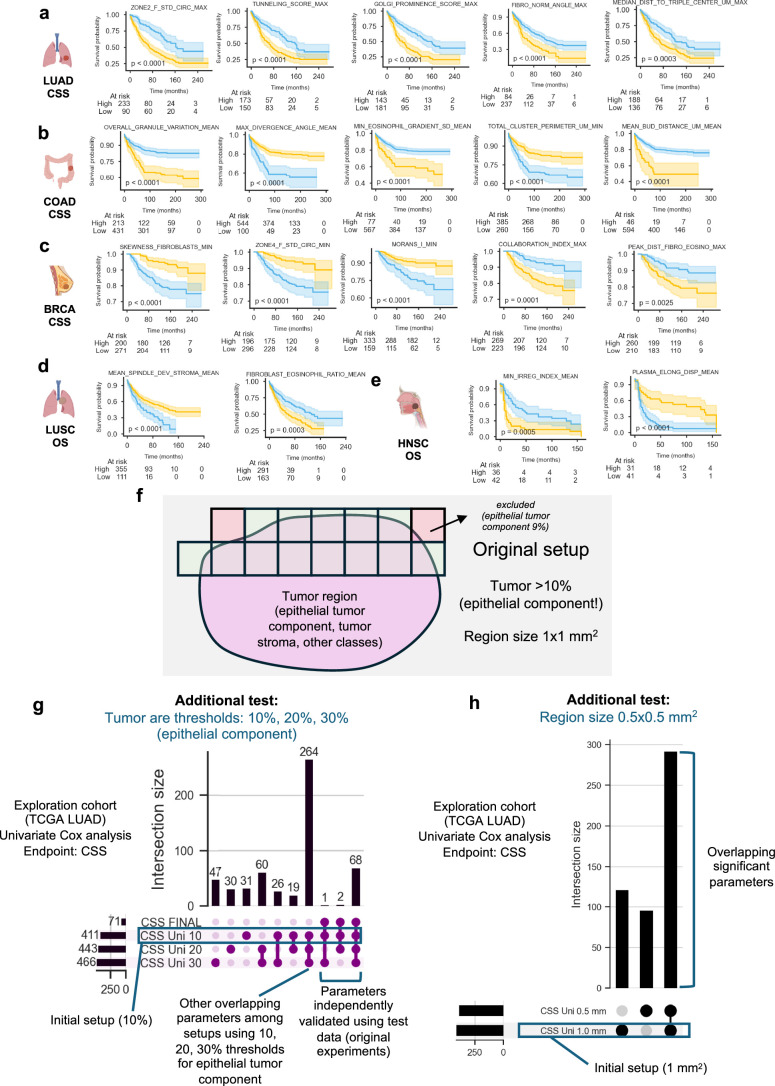
Prognostic analysis of SPARK parameters and validation of whole-slide image pipeline threshold choices. **a-e:** Examples of patient risk stratification using independently validated parameters in different tumors. Kaplan-Meier curves with 95% confidence interval and log-rank test. **f**: Initial analytical setup concerning region size and % of epithelial tumor tissue. **g**: Comparative testing of three different thresholds for % of epithelial tumor tissue in a region showing significant overlaps of detected parameters (exploration cohort) and with the final list of independently validated (test cohort) parameters. **h**: Comparative testing of two different region sizes 1.0×1.0 mm and 0.5×0.5 mm showing substantial overlaps in identified significant parameters (exploration cohort). Illustrations in **a**–**e** created in BioRender; Tolkach, I. https://biorender.com/bab1y26 (2026).

Kaplan–Meier curves illustrate clear separation of patient risk groups (Fig. 4i and Extended Data Fig. 4a–e). Full analysis for other tumor types is presented in Extended Data Fig. 3d–f and Supplementary Fig. 6 (full parameter lists and prognostic results appear in Supplementary Tables 8–17).

**Extended Data Fig. 5:**
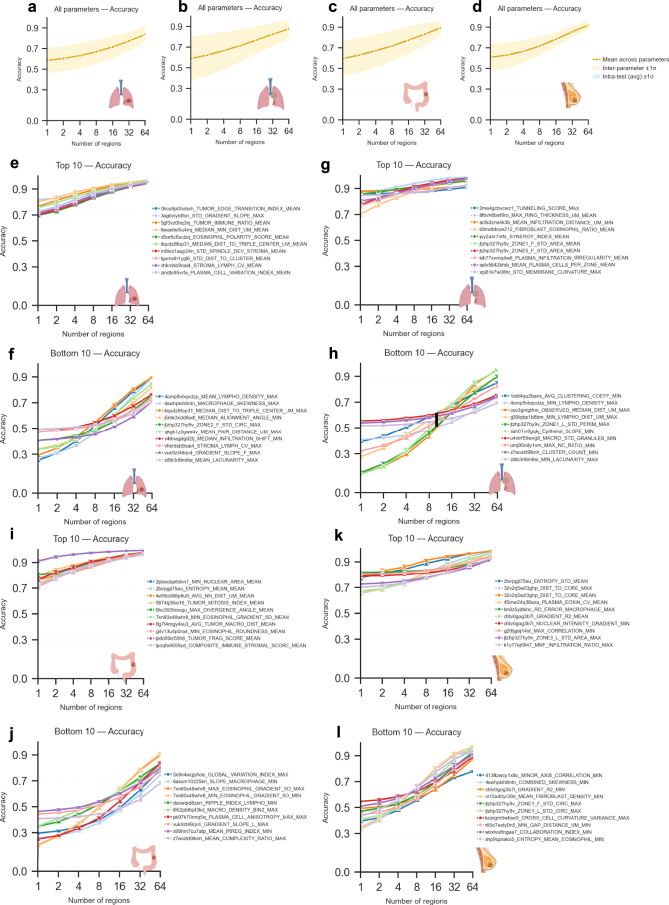
Analysis of performance for SPARK parameters in context of limited/missing data. We provide stability of prognostic risk classification of single patient cases when limited tumor area is analyzed: random sampling of 1, 2, 4, 8, 16, 32, and 64 regions from each tumor (1 region = 1 mm^2^), multiple sampling replicates (10 in each case). Accuracy of risk classification is measured using ground truth of prognostic risk classification for each parameter when the whole tumor was analyzed. The central measures (orange and green points) are mean of accuracy among replicates. All error bars are ± 1 standard deviation of accuracy across replicates for all or single parameters. **a-d**: Accuracy for all parameters (significant parameters in independent test cohort). **e-l**: Top and bottom 10 performing parameters for each tumor type and its most well characterized endpoint: lung adenocarcinoma/cancer specific survival (CSS; **e-f**; patient n = 324), lung squamous cell carcinoma/overall survival (OS, **g-h**, patient n = 154), colorectal adenocarcinoma/CSS (**i-j**; patient n = 645), and breast cancer/OS (**k-l**; patient n = 492). Top performing parameters are parameters showing high stability even if only small parts of tumor are being analyzed. Illustrations in **a**–**l** created in BioRender; Tolkach, I. https://biorender.com/bab1y26 (2026).

We confirm the robustness of our initial tumor region size selection (1.0 mm × 1.0 mm) and threshold for tumor area in the region (10% for epithelial tumor component) (Extended Data Fig. 4f–h) as well as robustness to data missingness (for example, small tumor volume/biopsy setting) (Extended Data Fig. 5).

We identified dozens of validated prognostic parameters per tumor type. To make them clinically useful, we combined them (for example, taking the top 6, 10, 20 and 30 parameters; see Fig. 4h for LUAD) into a transparent scoring system (Fig. 4j): for each patient, we count how many parameters fall into a ‘high-risk’ state; that sum is the prognostic score, which we then group into risk tiers. This approach allows for fine-granular stratification of patients based on risk using up to six prognostic groups (Fig. 4j). Including more parameters produced progressively smoother, monotonic risk gradients, indicating complementary contributions from individual parameters (Extended Data Fig. 3g,h). Full results across all five cancers and multiple endpoints are presented in Extended Data Fig. 3i–n and Supplementary Figs. 7–9.

### Human-initiated idea generation and prognostic testing

**Fig. 5:**
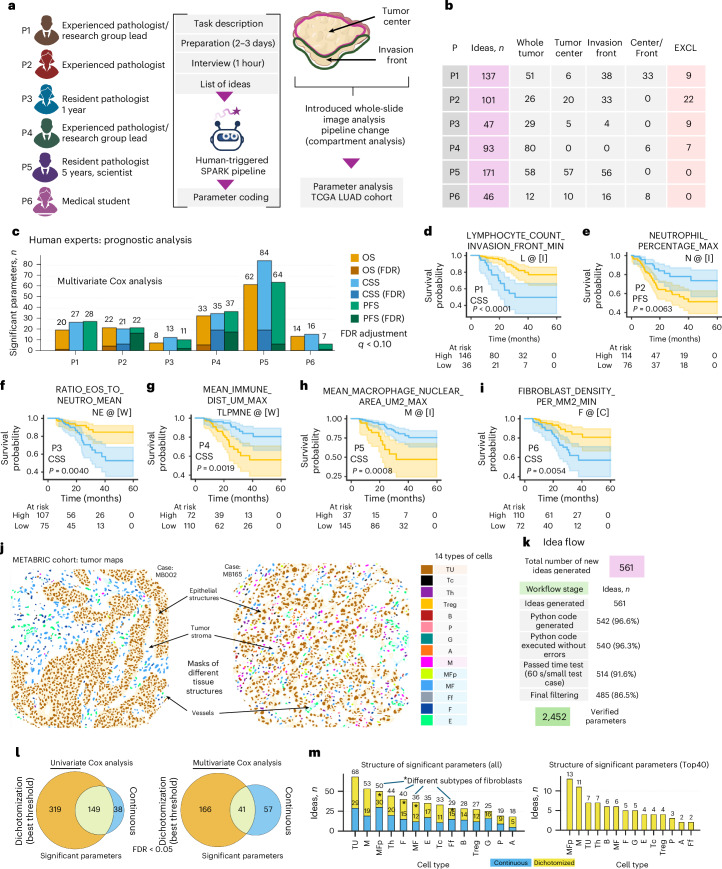
Human-initiated concept exploration and spatial biology exploration (Use Case 3). **a**, Human pathologists/analysts (P1–P6) participating in the experiment, and the principle of the experiment. Based on the pathologists’ request, additional functionality was integrated into the WSI analysis pipeline: differentiation between the tumor core (center) and the invasion front. **b**, Structure of provided ideas. EXCL, ideas excluded due to feasibility issues (for example, analyses involving vessels or the tumor surface). **c**, Validation of ideas and resulting parameters was performed using the TCGA LUAD dataset (independent test in merged LUAD test cohort in Extended Data Fig. 6p). The number of significant parameters (multivariate Cox analysis) is shown before and after FDR correction. **d**–**i**, Kaplan–Meier estimates (with log-rank test; 95% CIs) for representative significant parameters from P1–P6 (more examples in Extended Data Fig. 6a–o). **j**, Use case 3: examples of tissue microarray spots with primary breast cancer from the METABRIC cohort, showing 14 different cell types used as input in SPARK for idea generation (more examples with cell quantifications in Supplementary Fig. 10; cohort characteristics, idea generation outputs and parameter verification/decorrelation in Extended Data Fig. 7a). **k**, Idea generation and coding statistics. **l**, Use case 3: prognostic analysis (exploration cohort) included univariate and multivariate Cox analysis with multiple hypothesis testing correction (FDR < 0.05). Shown are overlaps for univariate and multivariate analyses when all parameters were studied as categorical and continuous variables. **m**, Use case 3: structure of significant parameters (exploration cohort, multivariate analysis with FDR correction) with regard to cell-type participation (left: all significant parameters; right: top 40 significant parameters based on hazard ratios in multivariate analysis). For analysis of top parameters, Kaplan–Meier curves and cross-validation see Extended Data Fig. 7b–h. @ [I], invasion front; @ [C], tumor center; @ [W], whole tumor; TU, tumor cells; Tc, cytotoxic T lymphocytes (CD8); Th, helper T lymphocytes (CD4); Treg, regulatory T lymphocytes; B, B lymphocytes; P, plasma cells; G, granulocytes (Eos + Neutros); A, antigen-presenting cells; M, macrophages; MFp, myofibroblasts PDPN+; MF, myofibroblasts, other; Ff, fibroblasts FSP1+; F, fibroblasts, other; E endothelial cells. Illustrations in **a** created in BioRender; Tolkach, I. https://biorender.com/5bomk3o (2026) and Tolkach, I. https://biorender.com/3pk210h (2026).

We extended SPARK to support clinician-driven concept exploration to see how far SPARK can support pathologists and researchers in targeted exploration of data. An additional module (Fig. 1d) turns free-text ideas and parameters into executable analyses (Methods). We invited five pathologists/researchers and one medical student to submit short, minimally formatted idea lists to test this workflow (Fig. 5a). Based on the pathologists’ request, we introduced a possibility of separate analysis of the invasion front and tumor core (Fig. 5a).

**Extended Data Fig. 6:**
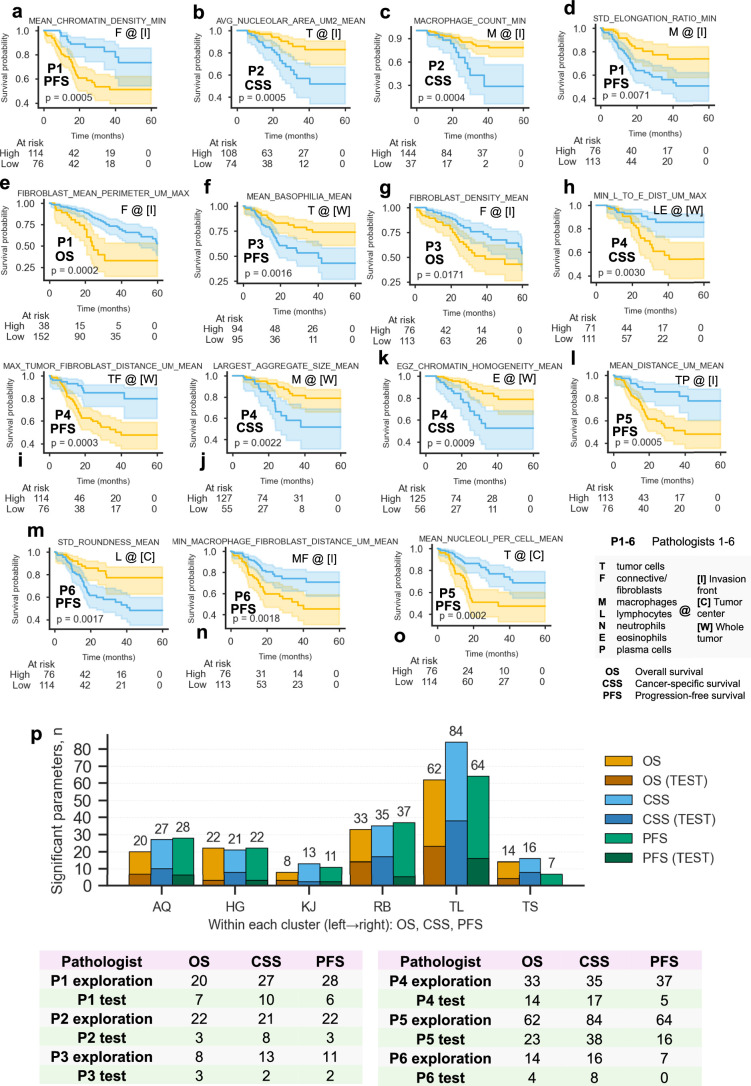
Human-initiated concept exploration. **a-o**: Kaplan-Meier estimates (with log-rank test; 95% confidence intervals) for representative significant parameters from P1–P6. **p**: Validation of significant human-suggested parameters in independent test cohort (Lung adenocarcinoma/LUAD; merged PLCO and NLST cohorts). For comparison shown are features significant in multivariate analysis (exploration cohort, without FDR adjustment) that could be validated at the same thresholds in the test cohort. Exact numbers of ideas are provided below.

We focused on LUAD. Only a few ideas were excluded, mostly because the required data were unavailable (Fig. 5b; for example, vessel detection or tumor surface features akin to COAD). The number of prognostically significant ideas varied across participants (Fig. 5c), likely reflecting differences in domain expertise, yet each contributor produced concepts with significant prognostic value. Representative results are shown in Fig. 5d–i and Extended Data Fig. 6a–o.

For most parameters, optimal thresholds were found in the exploratory TCGA dataset. We performed independent validation of found significant parameters using the merged PLCO/NLST LUAD cohort and confirmed that most parameters retain their prognostic value (Extended Data Fig. 6p); full results are in Supplementary Table 18.

Compared to SPARK-generated ideas, human concepts were typically simpler—often centered on a single cell type (for example, densities of lymphocytes or macrophages, chromatin structure of tumor cells, nuclear area of tumor and immune cells) with less spatial detail (for example, simple distances between two cell types, such tumor cells and macrophages or lymphocytes and fibroblasts). Nevertheless, this module gives researchers a flexible way to rapidly test diverse, user-defined biological hypotheses in malignant tumors.

### Use case 3, spatial biology idea generation and prognostic testing

**Extended Data Fig. 7:**
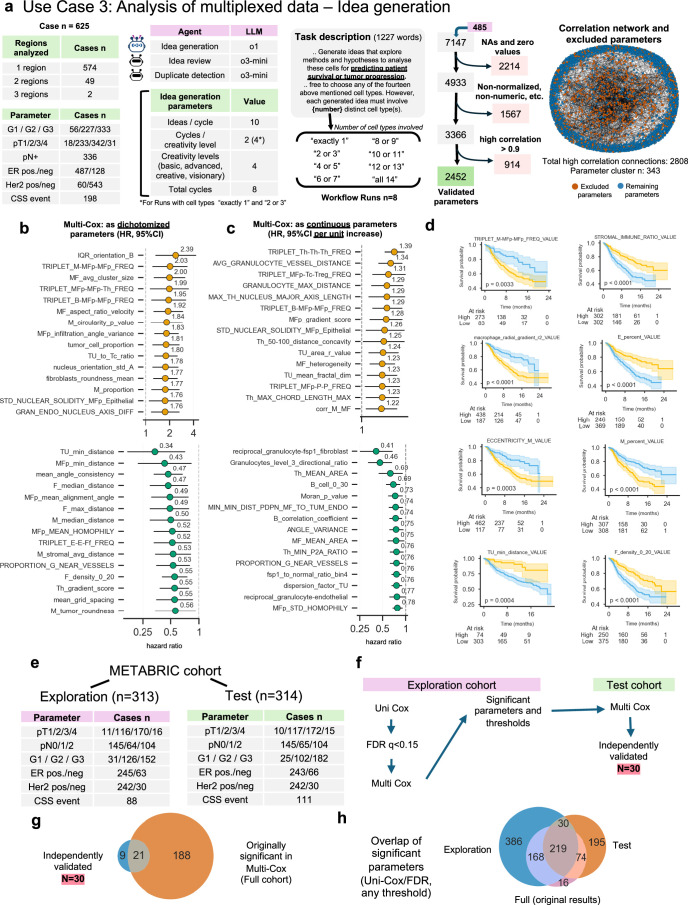
Spatial biology exploration using SPARK (Use Case 3). **a:** From right to left: METABRIC cohort characteristics, idea generation agents, task description, parameters verification and decorrelation statistics. **b**: Overview of significant parameters, as categorical variables (full cohort for exploration, patient n = 625, multivariate analysis). The central measures (orange and green points) are hazard ratio (HR). Error bars are 95% confidence interval for HR. **c:** Overview of significant parameters, as continuous variables (full cohort for exploration, n = 625, multivariate analysis). The central measures (orange and green points) are hazard ratio (HR). Error bars are 95% confidence interval for HR per unit increase. **d**. Kaplan-Meier estimates (with log-rank test; 95% confidence intervals) for representative significant parameters. e-h: Cross-validation approach for parameter exploration: exploration and test cohort characteristics (**e**), principle of cross-validation (**f**), independently validated parameters (test cohort) and comparison to initial list (full cohort) in **g**, overlap of significant parameters in exploration, test cohort, and full cohort (separate exploration in each cohort, same significant parameters shown independent of threshold – threshold can differ in each cohort, **h**).

Spatial biology methods generate valuable data on the TME that can inform the development of novel diagnostic tools and therapeutic strategies; however, the dimensionality of these data is high. We asked whether SPARK scales to spatial proteomic data with many cell types and significantly increased complexity. Using the METABRIC imaging mass cytometry cohort^32^ (14 cell types; Fig. 5j, cohort information Extended Data Fig. 7a, more examples in Supplementary Fig. 9), we made only minor task tweaks; the setup matched use cases 1 and 2 (Extended Data Fig. 7a). Because METABRIC images are whole tissue microarray (TMA) spots, each parameter yields a single value per case; no slide-level aggregation is needed. After routine checks and pruning of redundant parameters (Fig. 5k and Extended Data Fig. 7a), 2,457 parameters remained for Cox regression (full list in Supplementary Table 19 and the searchable HTML reference).

Given the exploratory nature and absence of an external test set, we controlled the false discovery rate (*q* < 0.05) and fit multivariable Cox models adjusting for grade, pT, pN, ER and HER2. This identified many parameters with independent prognostic value (Fig. 5l). Among the strongest signals (Fig. 5m) were parameters enriched for fibroblasts (and subtypes), macrophages and tumor cells—patterns consistent with our H&E analyses. Overall, the top parameters were new yet biologically grounded, emphasizing cross-cell interactions and spatial tissue organization (summarized in Extended Data Fig. 7b–d; full results in Supplementary Table 20, cross-validation confirming prognostic significance in Extended Data Fig. 7e–h).

### Tracing tumor evolution with SPARK

**Fig. 6:**
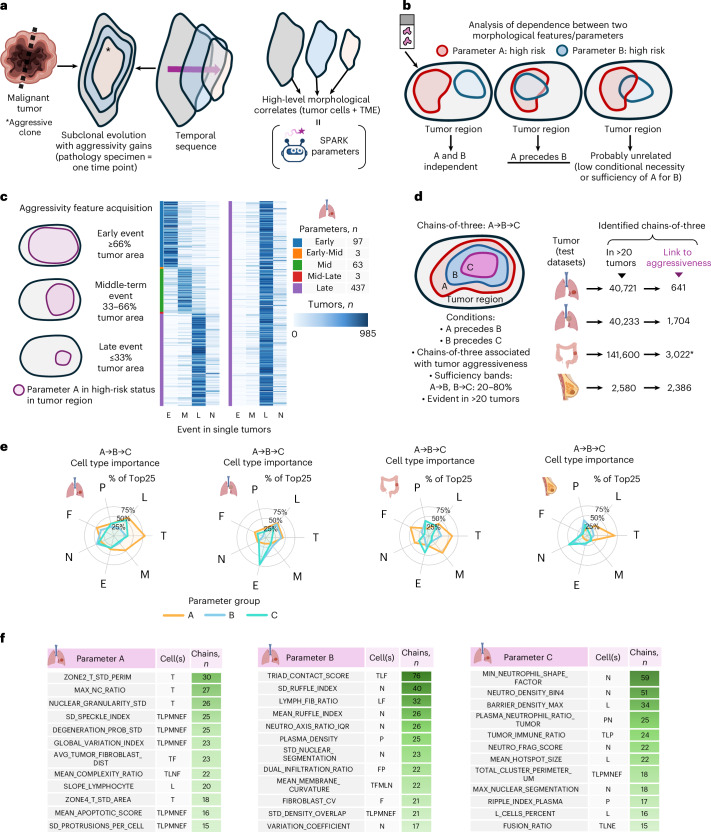
Exploration of temporal evolution of malignant tumors with SPARK. **a**, Molecular–genetic tumor evolution involves the sequential acquisition of driver alterations, giving rise to tumor subclones that seed increasingly aggressive descendants (intratumoral heterogeneity). These clones exhibit recognizable morphological traits. Considering this evolution as a field effect enables the tracing of the temporal sequence through detailed analysis of these traits. SPARK parameters can be regarded as such high-level morphological correlates of underlying molecular changes. **b**, Parameter dependencies. SPARK parameters allow deciphering the spatial relationships between two parameters (when they are transformed into areas with either low- or high-risk status (Methods)). Important are situations where A precedes B (evolutionary). **c**, Global timing of aggressivity features. We assume that early events (that is, parameters in the high-risk state) occupy a large fraction of the tumor. The distribution is presented for LUAD (merged PLCO, NLST and UKK cohorts; multiple WSIs per case, minimal preselection of slides). Results for other tumors are in Extended Data Fig. 9. **d**, Decoding the temporal sequence of events. We search for chains of three interrelated parameters, where A precedes B and B precedes C (A → B → C). Filtering conditions are shown (for details, see Methods) to identify recurring chains of three with robust associations to aggressivity. The number of significant chains identified is presented. *For COAD, given the high-quality follow-up data and the greater number of parameters with statistical significance, we apply stricter rules for links to aggressivity. **e**, Parameters that most frequently appear as A, B and C parts of the chains, focusing on the cells participating in these parameters. Radar plots show the importance of different cell types for early, mid-term and late stages of tumor development (that is, occupying the A, B or C position). Significant differences can be observed in the importance of cell types across different tumors and stages of tumor development, revealing insights into tumor biology. **f**, Review of the top A, B and C parameters and their frequencies within significant chains for LUAD (other tumors and comparison to global timing in Extended Data Fig. 9). E, early; M, middle-term; L, late; N, never. Illustrations created in BioRender: **a**, Tolkach, I. https://biorender.com/cwp82cd (2026); **c**–**f**, Tolkach, I. https://biorender.com/bab1y26 (2026).

We asked whether SPARK parameters—comprehensive descriptors of tissue morphology—can reconstruct how malignant tumors evolve over time (Fig. 6a). Tumor evolution proceeds as driver alterations accumulate, creating subclones with increasingly aggressive descendants. These subclones leave recognizable morphologic ‘footprints’ and contribute to intratumoral heterogeneity^33^.

Treating this as a spatial field effect (Fig. 6a) lets us infer temporal order from high-level morphologic patterns (Fig. 6b). We converted each parameter into high- versus low-risk spatial areas within tumors (Methods; parameters, thresholds and directions in Supplementary Tables 22 and 23).

#### Global timing

**Extended Data Fig. 8:**
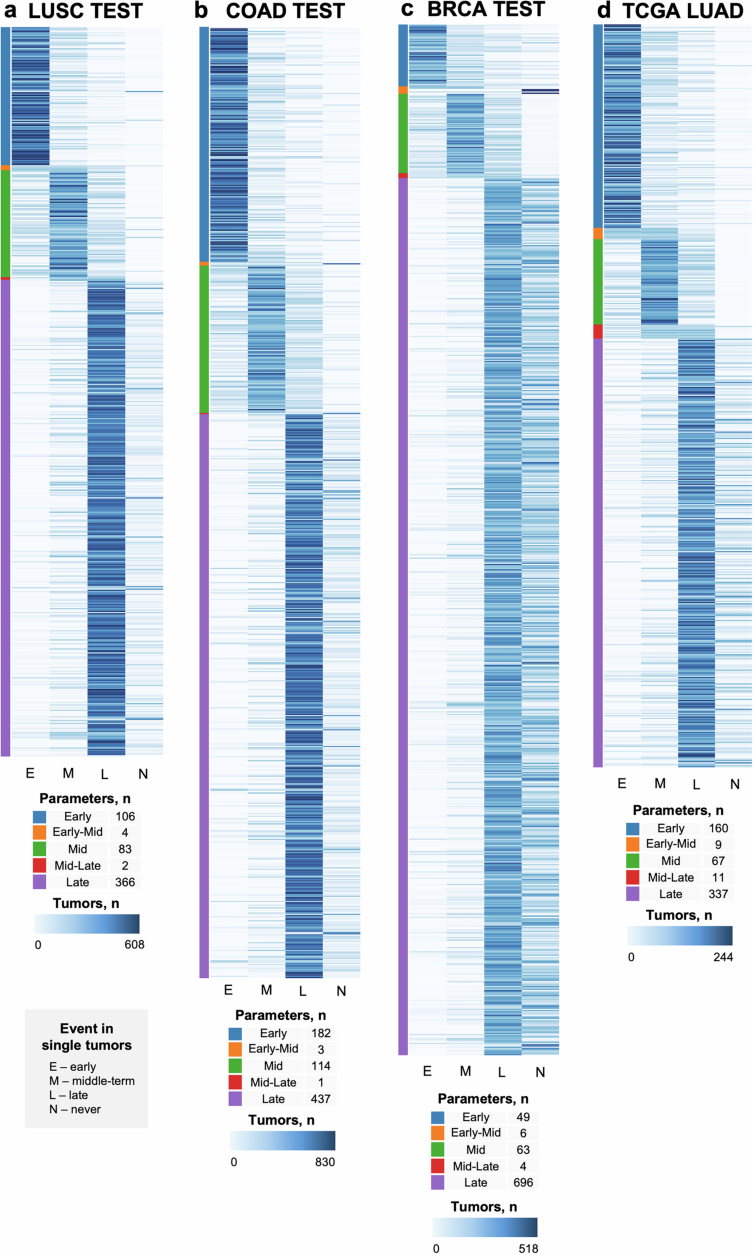
Analysis of global timing of SPARK parameters. Global timing of aggressivity features. We assume that early events (that is, parameters in the high-risk state) occupy a large fraction of the tumor. Based on this assumption, we map all parameters as early, early-mid, mid-term, mid-late, or late events. The distribution is presented for LUSC test cohort: merged PLCO/NLST/UKK (**a**), COAD test cohort: merge PLCO and HAL (**b**), BRCA test cohort: PLCO (**c**), and for comparison - LUAD exploration cohort (TCGA, **d**). We take test cohorts as they are more representative (more than 1 slide/tumor, less selection biases compared to TCGA cohorts).

Assuming early events occupy larger tumor fractions, we first estimated when aggressiveness features (expressed via SPARK parameters) arise (Fig. 6c). Across tumor types, >70% of features appear late (LUAD in Fig. 6c; other tumor types in Extended Data Fig. 8; full information in Supplementary Table 23), consistent with prevailing models. Notably, ‘early’ and ‘mid-term’ features likely mark early bottlenecks in acquiring aggressiveness.

#### Temporal sequences and cell-type roles

We next identified parameter chains (A → B → C) linked to aggressiveness (Fig. 6d). We called within-tumor precedence pairs and applied strict filters for directionality, conditional necessity and sufficiency, retaining only events clearly associated with risk (Methods). This yielded numerous sequences across LUAD, LUSC, COAD and BRCA (Fig. 6d).

**Extended Data Fig. 9:**
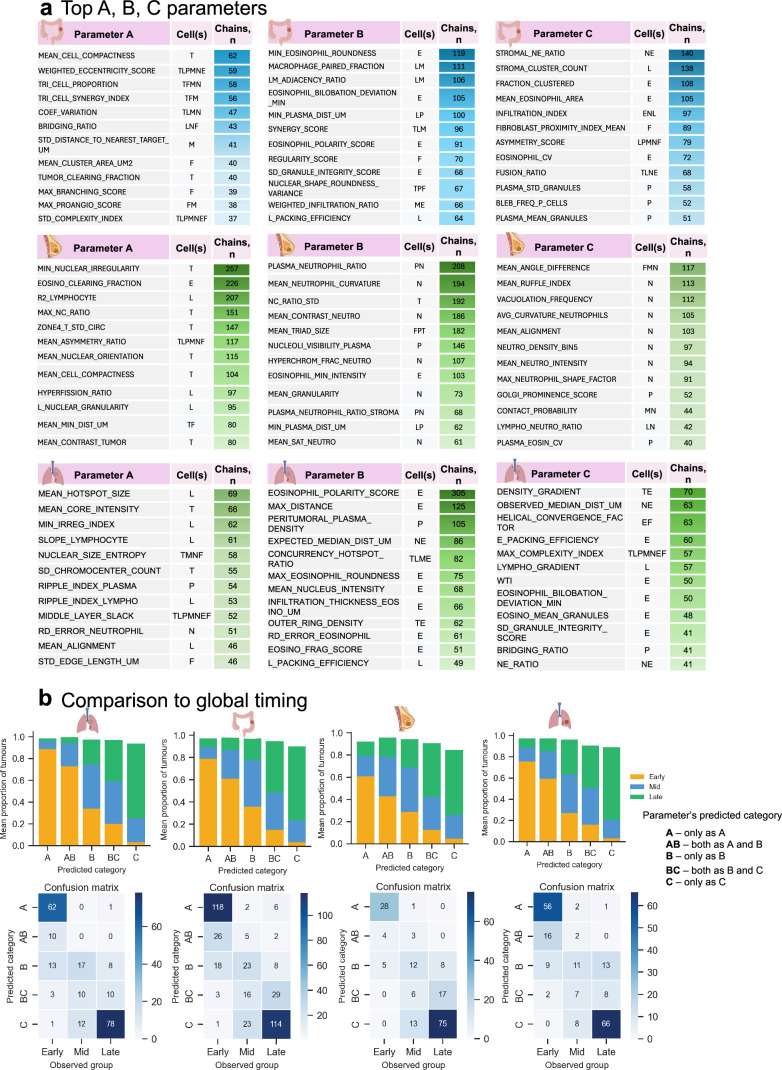
Role of SPARK parameters during temporal evolution of tumor. **a**: Review of the top A, B, and C parameters and their frequencies within significant chains for COAD, BRCA, and LUSC (all data for test cohorts). **b**: Identified A, B, and C parameters, as well as parameters appearing in both A and B or B and C positions within chains, are compared to the global timing of events (Fig. 6, Extended Data Fig. 9), showing high levels of concordance. Tumors: LUSC, COAD, BRCA, and LUAD. Illustrations in **a**,**b** created in BioRender; Tolkach, I. https://biorender.com/bab1y26 (2026).

Recurring positions within A, B or C highlight distinct cell-type dynamics by cancer type (Fig. 6e). In LUAD, early events involve high-risk features in tumor cells as well as fibroblasts and macrophages; neutrophils emerge later, in line with recent works^34^. In COAD (and LUSC), eosinophils and plasma cells are more prominent late, echoing recent reports including our own^7,12^. In BRCA, neutrophils become important late, whereas early phases are dominated by tumor-cell-intrinsic features (Fig. 6e). Parameter-level details are in Fig. 6f (LUAD) and Extended Data Fig. 9a (LUSC, COAD, BRCA). Finally, A/B/C assignments show meaningful, though incomplete, concordance with global timing (Extended Data Fig. 9b), suggesting that some sequences shift in time across tumors.

## Discussion

SPARK introduces a concept-centered framework for AI pathology tackling common limits of single-modality, task-specific models (Fig. 1b,c). It can autonomously propose, combine and manipulate biologically meaningful image concepts, and it outputs parameters that are fully interpretable and explicitly linked to tumor biology. Because it operates without task-specific training, new analytic tools can be prototyped within hours.

In this study, SPARK generated a large, unique and reusable library of fully interpretable biological concepts that significantly advance both grading of tumor aggressiveness and patient prognostic stratification. SPARK parameters characterize tumors with enough nuance to address higher-value questions, such as predicting therapy response, particularly in (but not limited to) immuno-oncology. In our PD-L1 status–prediction experiments across multiple clinically relevant cut-offs, SPARK captured a broader immune-evasion signature that could augment and extend purely PD-L1–based therapeutic strategies. Accordingly, we envision SPARK as a ‘companion’ algorithm in drug development and patient selection.

The SPARK principle is related to earlier attempts to create hand-crafted features for pathology applications. Most explainable or hand-crafted approaches—including our own—have focused on a few simple image features for prognosis^4–12,35^ with few exceptions^36^. Even with powerful foundational models^13–15,20^, prognostic AI still requires extensive supervised training, which is limited by scarce and unrepresentative data, single-tumor-type scope, constrained explainability and challenges in generalization^3,16,37–39^. In contrast, SPARK outputs concept-level parameters that are inherently interpretable and transferable. Although some overlap with classical metrics exists (for example, lymphocyte density or tumor–lymphocyte distance), most parameters are new.

Several other agentic workflows have been developed for pathology applications. Most of them, however, rely on multimodal approaches or VLMs and do not support the kind of unrestricted, transparent and reproducible reasoning that is possible with SPARK^25–28^. VLMs hold great potential for advancing reasoning; however, this class of models has not yet reached that level of sophistication^20,28,40^.

SPARK generated more—and more sophisticated—ideas than human analysts (Fig. 5a–i), especially for spatial analyses where manual reasoning quickly hits its limits. Crucially, analysts using SPARK overcame these limits: the system expands and structures hypotheses, enabling richer, more complex spatial concepts than humans typically produce alone.

Using simple models, SPARK parameters accurately recover estrogen status in BRCA and HPV/p16 status in HNSC, matching the performance of prior approaches^29,41–43^. In LUAD, we show that PD-L1 status can be inferred from H&E images with high accuracy across multiple clinical cut-offs, capturing broader signals of immune evasion with implications for immunotherapy selection. For MSI, SPARK achieves state-of-the-art accuracy, comparable to or exceeding benchmark studies and commercial tools^14,44–47^.

Beyond prediction, SPARK significantly enhances exploration in spatial biology: building on the METABRIC cohort^32^, we move beyond some classical aggregate cell-type abundances to map interactions among specific cell types and uncover new spatial patterns (Fig. 5j–m). SPARK is readily adaptable to diverse multiplexed assays with minor changes to the task and available cell types.

Finally, with SPARK we add a ‘third temporal dimension’ to two-dimensional pathology images (Fig. 6), recovering a disease ‘timeline’ from single H&E slides and indicating when different cell types enter the process. These trajectories can serve as a reference library and generate hypotheses for deeper tumor profiling with methods such as spatial transcriptomics.

SPARK’s design aimed to avoid a common risk of LLMs: fabricated outputs (hallucinations). During development, we saw little evidence of this, and no parameters were rejected for being implausible. We attribute this to a dedicated idea review step, careful task framing and the strength of current models; in our experience, clear task definitions were the single most important driver of performance. However, this might be an issue if less powerful models are to be used and should be carefully controlled for. We did not pursue full autonomy—automatically feeding cohort-level validation back into the system—because such open-ended workflows are costly. For idea generation and coding, we used state-of-the-art models (OpenAI o1 and Anthropic Claude 3.5 Sonnet); total development and implementation cost was ~4,000 €. As stronger open-source models continue to appear, SPARK can be adapted to reduce costs.

This study has limitations. A detailed review showed that some SPARK-generated parameters may need refinement; in most cases, this should be addressable by giving the model clearer instructions and by normalizing color-based measurements of nuclei and cytoplasm to improve generalizability. In our H&E analyses, we grouped connective-tissue-like cells under ‘fibroblasts’ for idea generation—a broad label that likely captured less-represented cell types (for example, endothelial cells). This H&E-only constraint does not apply to our spatial biology use case, where cell identities are more precisely defined. All cases and cohorts were surgical resections; because many clinical decisions rely on biopsies, validating the prognostic metric in biopsy tissue is an important next step. Predictive markers should also be tested in cohorts with therapy-response data. All cohorts were retrospective, so both the overall system and individual parameters require prospective validation in large patient cohorts representative of a broad range of tumor characteristics (for example, molecular subtypes), countries, ethnicities and surgical/oncological practices before they can be used to guide clinical decisions. Finally, in systematic post hoc experiments, we were not able to show the unambiguous effects of creativity levels on idea yields or diversity in saturated idea generation outputs (Supplementary Figs. 3 and 4). This requires further prospective validation.

Additionally, in scenarios—and likely future use cases—where SPARK yields hundreds or thousands of candidate biological concepts, questions of biological and scientific plausibility inevitably arise. Human reviewers may judge many concepts to be biologically plausible and to reflect measurable phenomena, but this is not the same as mechanistic validation (for example, in mouse models), which is naturally difficult (especially at scale) but is necessary.

## Methods

### Ethical aspects

The TCGA, PLCO and NLST cohorts are open-source datasets. Access to PLCO and NLST was applied for via the Cancer Data Access System portal (cdas.cancer.gov). All study steps were performed in accordance with the Declaration of Helsinki. This study was approved by the Ethical committee of the University of Cologne (20-1583), Halle/Cologne/Essen joint 22-1233 (Project FED-PATH/BMBF). Given the retrospective/archive nature of used data, the necessity of obtaining patients’ permission was waived by the ethical committee.

### Patient cohorts, H&E-based analysis

Multiple retrospective patient cohorts of primarily resected malignant tumors (LUAD, LUSC, COAD, BRCA and HNSC) were included in the study. Inclusion criteria were the presence of one or more representative H&E slides per tumor and the absence of neoadjuvant therapy. Detailed clinicopathological characteristics of patients are presented in Supplementary Table 1.

LUAD included five subcohorts: the TCGA cohort (source: >30 departments, mostly in the USA; *n* = 245), the PLCO trial cohort (multi-institutional trial, USA; patient *n* = 215), the NLST cohort (multi-institutional trial, USA; *n* = 206), the UKK cohort (part of the national FEDPATH study cohort; *n* = 561) and the UKE cohort (part of the national FEDPATH study cohort; *n* = 197). All LUAD subcohorts are resection specimens covering broad ranges of Union for International Cancer Control (UICC) pT/pN stages. The TCGA cohort contains information on CD274 (PD-L1) mRNA expression. The UKK/UKE subcohort cases partially contained information on PD-L1 expression on tumor cells (TPS). Overall survival (OS)/CSS clinical endpoint information was available in all subcohorts. PFS endpoint information was available in part of the cases in the TCGA, NLST and UKK subcohorts. In all analyses, when not stated otherwise, the TCGA cohort was used as the exploration cohort and the merged PLCO/NLST/UKK/UKE cohort as an independent test cohort.

LUSC included five cohorts from same institutional sources as LUAD: TCGA (*n* = 463), PLCO (*n* = 103), NLST (*n* = 86), UKK (*n* = 415) and UKE (*n* = 79). All LUSC subcohorts are resection specimens covering broad ranges of UICC pT/pN stages. The TCGA cohort contains information on CD274 (PD-L1) mRNA expression. UKK/UKE subcohort cases partially contained information on PD-L1 expression on tumor cells (TPS). OS/CSS clinical endpoint information was available in all subcohorts. PFS endpoint information was available in part of cases in TCGA, NLST and UKK subcohorts. In all analyses, when not stated otherwise, the TCGA cohort was used as the exploration cohort and the merged PLCO/NLST/UKK/UKE cohort as an independent test cohort.

The COAD collective included four cohorts: TCGA (*n* = 342), PLCO (*n* = 645), University Hospital Halle (HAL; part of the national FEDPATH study cohort; *n* = 183) and UKK (*n* = 257; only for the MSI prediction use case). All COAD subcohorts are resection specimens covering broad ranges of UICC pT/pN stages. The TCGA, HAL and UKK cohorts contained information on MSI status (TCGA: based on DNA analysis, MSI-high cases were considered a correlate to mismatch-repair deficiency in other cohorts; UKK and HAL: using mismatch-repair deficiency and immunohistochemistry and polymerase chain reaction confirmation in unclear cases). OS/CSS clinical endpoint information was available in all subcohorts. PFS endpoint information was available in the TCGA cohort. In all analyses, when not stated otherwise, the TCGA cohort was used as the exploration cohort and the merged PLCO/HAL cohort as an independent test cohort (prognostic analysis).

BRCA included two subcohorts: TCGA (*n* = 807) and PLCO (*n* = 492). All BRCA subcohorts are resection specimens of female patients covering broad ranges of UICC pT/pN stages, hormone receptor status and different histological subtypes. Histological grading was available in the PLCO cohort and not available in the TCGA cohort. OS/CSS clinical endpoint information was available in all subcohorts. PFS endpoint information was available in the TCGA cohort. In all analyses, when not stated otherwise, the TCGA cohort was used as exploration cohort and the PLCO cohort as an independent test cohort.

The HNSC use case included two cohorts: UKK (*n* = 140) and University Hospital Giessen (only for the HPV-status-prediction use case; *n* = 152; HPV/p16-positive *n* = 35 (ref. ^29^)). The UKK cohort is a resection cohort covering broad ranges of UICC pT/pN stages with available information to the OS clinical endpoint. In all prognostic and correlation analyses, the TCGA LUSC cohort (both squamous cell carcinomas) was used as the exploration cohort and the UKK cohort as an independent test cohort.

### Patient cohort, multiplexed tissue data analysis

For the analysis of SPARK using multiplexed tissue characterization data, we used the METABRIC BRCA cohort^32^ with available imaging mass cytometry data. A total of 625 primary tumors were available for analysis, with one TMA region analyzed in most patients (Fig. 5j). For all patients, information on pT (tumor size), pN, histological grade and receptor status was available (see Extended Data Fig. 8a for details).

### WSI analysis pipeline, input data types

We developed a computationally efficient pipeline for preprocessing single WSIs as input objects for SPARK (Fig. 1f). This pipeline integrates multilayered information generated by different algorithms, which is efficiently preprocessed and stored as WSI objects (for preprocessing details, see below). The steps include tissue detection, quality control, multiclass tissue segmentation, tumor region mask filtering and single-cell segmentation and classification.

### WSI analysis pipeline, quality control and foreground/tissue segmentation

We used our previously developed GrandQC tool^48^ for tissue detection in WSIs and segmentation of artificially changed regions (seven types of artifacts; using MPP1.5 version), which were excluded from any downstream analysis.

### WSI analysis pipeline, multiclass tissue segmentation

Previously developed and extensively validated organ-specific semantic segmentation models were implemented to produce detailed multiclass tissue segmentation maps. Briefly, these models are based on UNet++ decoders with EfficientNet encoders and were trained on large, high-quality, manually annotated datasets. They analyze WSIs in 512 px × 512 px regions at a microns-per-pixel resolution of 1.0, roughly corresponding to ×10 objective magnification. In independent validation using external datasets, these models demonstrated excellent generalization, achieving Dice scores above 0.89 for multiclass segmentation. For details on development and validation, we refer to the original studies^4,49^.

For lung cancer, the model detects 12 classes^4^; for colorectal cancer, 11 classes^49^; and for breast cancer, 12 classes. All models identify tumor-relevant classes (epithelial and stromal compartments, necrotic debris, mucin, tertiary lymphoid structures) as well as numerous benign classes. All WSIs from all cohorts were processed with the models to generate multiclass tissue segmentation masks (example in Fig. 1f). From these masks, all classes other than tumor (epithelial tumor compartment) and tumor stroma were excluded. These two classes underwent minimal postprocessing to remove false-positive tumor and stroma detections, retaining only tumor stroma adjacent to tumor. The filtering methodology for tumor regions was adapted from our recent study^12^. For the HNSC cohort, given its shared morphology with LUSC (squamous cell carcinoma), the lung cancer model was used for multiclass segmentation. However, this model does not include all benign classes relevant to HNSC (for example, squamous mucosa, specific submucosal glands), and HNSC slides often exhibit numerous mechanical or electrocauterization artifacts. To address these limitations, an experienced board-certified pathologist (Y.T.) annotated the tumor regions to be analyzed. The lung cancer model was then applied to these annotated regions for automatic segmentation.

### WSI analysis pipeline, single-cell detection and classification

Previously developed single-cell detection and classification for H&E was used to detect and classify all cells within the filtered tumor region (Fig. 1f). Briefly, this algorithm was developed using a large dataset of manual annotations (cell *n* = 1,272,506) for 8 cell types (tumor cells, benign epithelial cells, lymphocytes, plasma cells, macrophages, neutrophilic granulocytes and eosinophilic granulocytes) and 8 different tumor types (including all tumor types studied in this work). Macrophage ground truth was generated using immunohistochemistry stain for CD163, which was transferred to H&E images. The model was trained using the HoverNext algorithm^50^ with a convnextv2_large backbone at microns per pixel 0.5 (approximately ×20 magnification) and demonstrated segmentation/classification performance comparable to or better than recent state-of-the-art algorithms in independent validation^51^. All WSIs from all cohorts were analyzed using the model (example output in Fig. 1f). Additional postprocessing was applied to remove potential false classifications: (1) all cell detections outside tumor and tumor stroma regions in filtered masks were discarded; (2) very small objects (minimum cell-polygon size in µm^2^: tumor cells, 15; connective cells, 6; all other cell types, 4) were removed; (3) all ‘benign’ and ‘connective’ detections in the epithelial tumor compartment were relabeled as tumor cells (given the review showing this type of misclassification and robustness of tumor region segmentation for this filtering); and (4) all ‘tumor’ and ‘benign’ detections in the tumor stroma (mostly single cells in isolated regions) were assumed to be false positives and discarded. All filtered cell detections (nuclei polygons with cell-type classification) were saved as GeoJSON files for SPARK input.

### Preprocessing of multiplexed data

First, we excluded TMA regions containing only benign tissue or fewer than 10 tumor cells. Compared to WSI analysis, we used a slightly different approach for METABRIC. As only small regions (TMA spots, ~1 mm) were available, we did not use tiling and instead applied SPARK to the entire region. Tissue segmentation masks and single-cell detection/classification masks were prepared as input for the SPARK pipeline. Tissue segmentation masks included the epithelial and stromal tumor compartments and were used by SPARK to identify the affiliation of each single cell.

We also extracted blood vessel areas from the mask and binarized this information. These vessel areas were filled and then converted into disjoint polygons. Along with the cell objects and tissue segmentation mask, a list of polygons for vessel objects (as an additional subregion within the stroma) was also used as SPARK input. All epithelial tumor cell classes were merged into a single class, and all single-cell detections (nuclear polygons) for a total of 14 cell classes were transformed into GeoJSON masks for use as SPARK input (Fig. 6j).

### SPARK agentic workflow, modular structure

We developed SPARK as a modular agentic workflow, where the output of each step serves as the input for subsequent steps (Fig. 1c). SPARK comprises four main agentic modules: idea generation, idea refinement, idea/parameter coding and parameter verification. An additional module was implemented to enable human interaction and human-triggered idea generation (Fig. 1d).

### SPARK idea generation pipeline

The principle is outlined in Fig. 1c. Three agents are involved in this task. The main agent (Idea Generation Agent (IGA)) receives a task description that includes the clinical context, goal (ideas with prognostic or other relevant implications; Fig. 2a), available data, output formatting (JSON), restrictions and other pertinent information. The IGA generates ideas in predefined cycles. To make the idea generation process more systematic, we define three parameters to guide these cycles: the number of cells involved in an idea, the ‘quality’ of the idea and the number of ideas generated per cycle (Fig. 2a). After each cycle, the Idea Review Agent evaluates the generated ideas for adherence to the provided guidelines (task description), and all qualified ideas are then checked by the Idea Duplicate Detector Agent (IDDA). The IDDA compares each generated idea against every original idea already stored in the ‘Raw Idea Database’. It uses a composite similarity score, based on predefined metrics (0–10), to determine whether an idea is a duplicate and should be discarded. All ideas with a similarity score >8 against any idea in the database are declined as duplicates. All original ideas saved in the Raw Idea Database receive unique identifiers (idea_uid). Starting from cycle 2, all original idea titles from the Raw Idea Database are provided to the IGA to reduce the number of duplicates among newly generated ideas.

### SPARK idea refinement pipeline

During this step, the Idea Refinement Agent takes one idea at a time from the Raw Idea Database and, using the given task description (including information on available data, coding structure of the WSI pipeline and the structure and coding nomenclature of WSI object features—that is, EvalObj, described in the next section on preprocessing), provides a detailed, step-by-step analytical and technical implementation of the parameter(s) defined in the idea. In other words, it specifies a clearly defined process from the WSI object to the parameter outputs for that WSI (Fig. 1c). All refined ideas, now containing the added technical implementation, are saved in the Refined Idea Database (JSON file) to be sampled for coding in the next step.

### Preprocessing of WSI objects for SPARK

We preprocess and preload the data before passing it to the coding agentic module. These steps produce a precomputed, well-structured backbone of information that (1) reduces computation time per parameter (during coding and deployment at later stages) and (2) improves the quality and reliability of agentic coding. For each WSI, we collect and package information from three sources into a WSI object (Fig. 1f): the WSI itself, a tissue segmentation mask (tumor and tumor stroma compartments) and a GeoJSON file containing cell detection/classification information (as outlined above).

Because the coding agent (and later, parameter deployment for analyzing large cohorts) is designed to operate on smaller tumor regions (for example, 1 mm × 1 mm) and the generated parameters focus on single-cell analysis, regions and cells are the central objects processed as described below. For the standard setup (use cases 1 and 2: autonomous idea generation), in the first step, we read the information from the GeoJSON, producing a list of cell objects. Each cell object represents a single cell, with the following information initially stored in the GeoJSON: the cell polygon (coordinates in the WSI pixel coordinate system) and the cell type. We map each cell’s centroid to the tissue mask, classifying the cell as belonging to the epithelial or stromal compartment. Additionally, we attach a small image—extracted from the WSI and containing the cell nucleus—to each cell. Using the bounding boxes of the cell polygons, we create R-trees for fast spatial lookup.

For each cell, the following attributes are available and accessible for parameter coding by SPARK:

- ‘index’: index in the list of cells
- ‘cell_class’: cell type
- ‘polygon’: shapely polygon of the cell nucleus (pixel coordinates in the original WSI)
- ‘CentroidPix’: centroid of the cell nucleus (pixel coordinates in the WSI)
- ‘in_mask_set’: a set that describes in which tissue type(s) the cell is located (single entry: ‘Tumor’ or ‘Stroma’)
- ‘ImageColor’: small RGB Pillow image that contains the cell nucleus with 2 px padding around the bounding box of the cell
- ‘ImageGrey’: grayscale image (np.ndarray) that contains the cell nucleus (same region as ImageColor)
- ‘ImageMask’: numpy.ndarray with True/False values, cell nucleus mask, corresponding to polygon, ImageColor and ImageMask

In the second step, we split the WSI into square regions (‘patches’) with a side length of 1 mm. This region size was empirically chosen, based on consensus among participating expert pathologists, as the minimal spatial unit within a tumor region that could have prognostic or other clinical implications (roughly corresponding to a subclone definition, where certain features may influence clinical endpoints).

For each patch, we generate a list of cells within it, using the centroids of their polygons for unique mapping. Cells may be assigned to multiple patches if their centroid lies exactly on a boundary—this occurs in ~ 5 out of 100,000 cells and has no practical implications.

Each patch is also assigned a cropped version of the tissue mask, downscaled by a factor of 16 relative to the WSI for computational efficiency. This mask can be used to analyze the size and shape of tumor and stroma areas within the patch. If an additional comparison between the mask and the cell positions is required—such as evaluating tumor cells near the tumor–stroma boundary—the coding agent should use the provided function downscale_point_into_local_area(point_xy, patch_id). This function adjusts for both pixel offsets (entire WSI versus local/patch coordinates of the cropped image) and scaling differences.

Using the mask, we label patches as significant if they contain at least 10% tumor area; only these patches are analyzed. In the final function library, each idea is computed using the function calculate_features_fun(Eval_Obj, sign_indices, cell_ids_in_patches, patchwise_tissue_masks, output_file_NEW).

The Eval_Obj (= collection of patches) contains the Eval_Obj.single_cell_list and the function Eval_Obj.downscale_point_into_local_area(point_xy, patch_id). The three parameters—sign_indices, cell_ids_in_patches and patchwise_tissue_masks—are lists encoding patchwise information. Each patch is assigned a unique index (for reproducibility and for comparing outputs from different functions) that contains a list of cell indices mapping to the cell object list and is linked to a cropped, scaled version of the tissue mask. Our pipeline also supports larger patch sizes, which can be selected by the coding agent (for example, 5 mm or more). The coding agent can choose the patch mode best suited for the task (a fixed choice per feature) by setting patch_mode = ‘standard’ or patch_mode = ‘large’. However, all but a few features operate with 1 mm^2^ patches; we restrict the analysis to these.

### SPARK idea/parameter coding pipeline

A single idea typically contains one or more parameters necessary for its implementation. The Idea Coding Agent (ICA) sequentially samples ideas—one idea per iteration—from the Refined Idea Database. Based on the detailed task description specifying how the Python code snippet for the parameter(s) should be structured (that is, a Python function integrated into the WSI analysis pipeline at a predefined position), the ICA generates a code snippet that executes the idea on a patch using precomputed structures. Each code snippet includes, at the end of the file, a commented section with a textual description of the parameters and the underlying measurements. For each idea, the code snippet is tested for functionality using a prepared test case (10 selected patches from a TCGA slide) as part of the WSI analysis pipeline (Fig. 1c).

If the analysis run fails due to a code error, the initial task description, the generated Python snippet and the stdout output containing the error description are provided to the Code Review Agent (CRA). The CRA may review and optimize the code up to three times (Fig. 1c) before declaring the idea as ‘failed’. If the code is functional but exceeds a runtime threshold of 60 s (for 10 test patches, only execution of idea-snippet measured), a timeout error is raised, and the snippet is passed to the CRA with the explicit task of finding a faster implementation.

### SPARK parameter verification pipeline

After successful coding, parameters are ready to be implemented on the whole cohort using our developed WSI analysis pipeline. Each snippet is converted into a callable function, resulting in one comma-separated values file per WSI and idea, containing regional measurements (analyzed patches = rows) for all parameters coded for that idea. An additional agentic module processes these comma-separated values files post-analysis to perform further verification of parameters before they are used in any downstream analysis concerning clinical endpoints. This review includes both non-agentic and agentic components. The non-agentic component excludes all parameters with >80% (empirical threshold) of measurements equal to 0 or NA (missing). The agentic component reviews the idea description, the produced code snippet and the included comment describing exactly what the parameters measure and excludes parameters that are non-normalized (for example, raw cell counts without area normalization), non-numeric parameters or parameters with any significant deviations. The result of this workflow stage is two lists: excluded parameters and verified parameters. Verified parameters can then be used in further downstream analysis.

### SPARK human-triggered idea generation

We developed an adaptation of SPARK for human-triggered idea generation (Fig. 1d). For this, we created an additional module consisting of two agents. The List Formatting Agent takes notes provided by human pathologists and transforms them into a more structured idea formulation. The Idea Formulation Agent converts these ideas into a structured SPARK-style JSON format, resulting in a Raw Idea Database that can then be passed through the Idea Refinement and Idea/Parameter Coding steps, similar to SPARK-generated ideas. This enables rapid implementation of ideas and deliberate exploration of cohort data by human experts, while skipping the autonomous idea generation stage.

### Automatized invasion front versus tumor center detection

All human pathologists’ inputs are encoded using the above WSI preprocessing framework. The patch size remains the same as previously defined, 1 mm (patch_mode = ‘standard’). The experimental component involving human pathologists (Fig. 5a) revealed a request for separate analysis of the tumor core/center and the invasion front. To accommodate this specific request from the pathologists (expressed by P1), we labeled the patches as belonging either to the tumor core (center) or to the invasion front, using our highly detailed multiclass tissue segmentation masks, which also include benign tissue areas, following the approach described below.

Given the benign area (coherent regions at least 500,000µm^2^ in area, then inflated by 1,000 µ into any unlabeled/background regions) as a reference, tumor within a distance of 800 µ is labeled as an invasion front; the remainder is labeled as core. A patch is then classified as either front or core depending on which tissue label is dominant (that is, has the highest number of mask pixels within the patch). Due to the frequent intermixing of tumor and stroma, using the benign tissue as a reference proved to be the most robust and reliable way of defining these tumor layers. However, this approach naturally depends on the invasion front being captured in the WSI—which is not always the case (sometimes only the tumor core is present). In such cases, analysis of parameters involving invasion-front measurements is not possible, and therefore these cases had to be excluded.

### Agentic framework and task definition

We follow a typical agent–task–crew–flow–tool paradigm within the crewAI framework. All tools are implemented as extra-agentic in the accompanying pipeline code to reduce the token usage required to accomplish the task. Task formulation for agents is crucial for proper execution by the agent. All formulations were iteratively fine-tuned during the development phase until the desired output was achieved, and all edge cases that could lead to potential failures were properly addressed. For agent descriptions and task formulations, we refer to the code (Code Availability; agents, /config/agents.yaml; tasks, /config/tasks.yaml). Empirically, agentic memory within a task or crew in our workflow setup proved to be non-beneficial, generated substantial additional costs and was therefore disabled at all stages.

### LLM selection

Selection of the LLMs as specific agents was motivated by extensive initial tests during development and the following considerations. First, idea generation is the most critical stage, where substantial reasoning efforts are required. Therefore, we selected the best available LLM with strong reasoning capabilities at the time of the experiments (January–February 2025), o1 (full version) from OpenAI. Second, given the significant costs of o1 (15 € per 1 million input tokens, 60 € per 1 million output tokens), we chose a simpler model (o3-mini from OpenAI) for tasks that do not involve reasoning and require high output volumes (in tokens). This includes, for example, the Idea Review Agent and IDDA in the idea generation workflow. This choice proved to be highly effective in terms of both accuracy and cost. Third, the LLM for the ICA needed to be highly capable, given the relative complexity of the task (integration into the existing WSI analysis pipeline, handling multiple objects and their features) and the resulting parameters. For this purpose, we selected Claude Sonnet 3.5 (Anthropic) as the LLM of choice. This decision, consistent with benchmarking results on general coding tasks at the time of the experiments, was based exclusively on performance analysis through direct comparison of GPT-4o, o3-mini, o1 and Claude Sonnet 3.5 in coding 30 ideas, followed by manual review of the generated snippets. The results revealed a significantly better understanding of the task and higher-quality implementation by Claude compared to o1, whereas GPT-4o and o3-mini performed markedly worse.

### Open-source LLM selection and deployment

The critical steps (idea generation and idea coding) were reproduced using open-source, local LLMs. We deploy them using either an AI server (4× NVIDIA A100 80 Gb; ‘S’) or a consumer-grade laptop (Apple MacBook Pro 64 Gb RAM, M1 Max CPU; ‘L’). We choose seven models: four generalist models (model/deployment: gpt-oss-120b/‘S’, gpt-os-20b/‘S’, distilled DeepSeekR1-Llama3-70b/‘S’ and Qwen3-32b/‘L’) and three LLMs fine-tuned on the medical domain (Meditron-70b/‘S’, MedGemma-27b/‘L’ and BioMistral-7b/‘L’). ‘S’ models were deployed using vllm v.0.11.0, and ‘L’ models were deployed using ollama v.0.12.3.

### Parameter decorrelation

We performed decorrelation of SPARK-generated parameters before any downstream analysis. This process resulted in the removal of a subset of parameters that showed strong correlations with others. First, interparameter relationships were quantified at the region level (technical correlation: that is, two parameters effectively measuring the same feature) by computing pairwise Pearson correlation coefficients and associated *P* values for all parameters. For each parameter, the most strongly correlated counterparts were identified and tabulated together with their correlation coefficients. Second, parameters exhibiting high correlation (above a defined threshold; Results) were considered redundant. These were iteratively removed to retain a subset of minimally correlated parameters. The selection process was guided by correlation network visualizations to ensure comprehensive coverage of relevant parameters while reducing multicollinearity. This procedure resulted in highly correlated parameters forming connected clusters. From each such cluster, only one parameter was preserved and the others were discarded.

### Case-level aggregation

Prognostic analysis requires case-level aggregation of single-parameter values. All generated parameters, including SPARK parameters and parameters from experiments involving human pathologists, have multiple regional measurements for a single WSI and (in many cohorts) multiple WSIs per case. For each parameter, we compute three derivatives aggregated over all measurements and all available WSIs: minimal, mean and maximal values. For use case 3 (analysis of multiplexed data), for a limited number of cases, several TMA spots were available. Parameter values were aggregated to the case level using averages.

### Multiple hypothesis testing considerations

As deployment of autonomous idea generation with SPARK results in numerous parameters, we apply false discovery rate (FDR) correction using the Benjamini–Hochberg procedure, accounting for the number of tests performed, in cases where downstream analysis involves simultaneous testing of multiple parameters (for example, correlation analysis, prognostic analysis for all use cases). For Cox models, we apply separate correction for each prognostic endpoint (both categorical and continuous approaches).

### Training of predictive models

An XGBoost classifier (v. 2.0) was employed for a binary classification task using pathomics features and clinical parameters (only for MSI prediction). We selected XGBoost for state-of-the-art performance on tabular data, interpretability (compared to deep learning methods), regularization and excellent handling of imbalanced datasets and error correction capabilities in consecutive decision trees (compared to the Random Forest approach). The model was implemented via the scikit-learn-compatible XGBClassifier interface, using a binary logistic regression objective and the area under the ROC curve (AUROC) as the evaluation metric.

Hyperparameter tuning was performed with Optuna, employing tree-structured Parzen estimator sampling and a fixed random seed to ensure reproducibility. The optimization process maximized fivefold stratified cross-validation ROC-AUC over 60 trials. The hyperparameter search space used reasonable defaults and was consistent across all trained models: n_estimators: 300–2,000, learning_rate: 1 × 10^−3^ to 0.2, max_depth: 3–10, min_child_weight: 1 × 10^−2^ to 10.0, subsample: 0.5–1.0, colsample_bytree: 0.5–1.0, min_split_loss (gamma): 0.0–5.0, reg_alpha: 1 × 10^−8^ to 10.0, reg_lambda: 1 × 10^−8^ to 10.0. Class imbalance was addressed by automatically computing and applying scale_pos_weight as the ratio of negative to positive samples.

Model selection employed fivefold stratified cross-validation with shuffling to ensure balanced representation of target classes across folds. For each fold, early stopping with a patience of 50 rounds was applied to prevent overfitting, monitoring validation AUC. After hyperparameter optimization, the final model was trained on the full training dataset using the optimal parameters. A validation split (20% of the training data) was generated via stratified sampling for early stopping during the final model fitting. Model performance was evaluated using ROC-AUC on independent test datasets.

Model interpretability was enhanced using SHAP values. A background dataset of 100 randomly sampled training instances was used to compute SHAP values for all test samples, and parameter importance was quantified as the mean absolute SHAP value.

### RNA expression data extraction and preprocessing

We use normalized mRNA expression data for CD274 (PD-L1) for LUAD and LUSC. This data was downloaded from the TCGA portal of the Broad Institute (http://gdac.broadinstitute.org).

### Prognostic analysis

During prognostic analysis, we always use one cohort per tumor type for exploration purposes (identifying relevant parameters and thresholds), and all other cohorts serve as independent testing cohorts. We implement two types of analysis for each parameter: treating the parameter as a continuous variable, and dichotomization using the optimal threshold. To determine the optimal threshold, we examine a range of quantiles for parameter values across the entire exploration cohort. We select the quantile range Q20–Q80 to reduce the likelihood of outlier-driven results due to small subgroups. Univariate and multivariate Cox analyses with FDR-adjusted *P* values are performed, as well as Kaplan–Meier estimates with the log-rank test, primarily for visual inspection. We test the proportional hazard assumption for each parameter in univariate (*P* < 0.01) and multivariate (relaxed to *P* < 0.05) models. All parameters failing the proportional hazard assumption were additionally tested using a spline-based Cox proportional hazard model with time-varying covariates for their prognostic significance. However, as we observed that the majority of such significant parameters were already included in classical Cox analysis (as another case-level aggregation derivate), we concentrate only on parameters that passed the proportional hazard assumption test in all experiments.

For dichotomization-based analyses, we perform additional decorrelation of parameters using the following principle: we compare each parameter against every other parameter and compute the Jaccard score for the overlap of low- and high-risk groups. We then apply the same network analysis described above to identify highly correlated clusters and retain only one parameter per cluster (Jaccard score threshold: 0.85).

We also compute the *C*-index for both the baseline models (including only clinicopathological variables) and all multivariate models and report the Δ*C*-index (difference between the two) along with the likelihood ratio test *P* value and calibration slopes.

### Batch effect detection and automatized parameter review

Given multicentric test cohorts, we analyze parameters for potential batch effects in prognostic and temporal analysis. For every (parameter, project) combination, we generate the number of cases with data, number of cases without data, mean, standard deviation, median, unscaled median absolute deviation (MAD), 25th/50th/75th percentiles and range. To test for cross-project distributional shifts, we applied a Kruskal–Wallis test on the case-level proportions (reporting *H* and *P*) and derived the epsilon-squared effect size. Robust dispersion was summarized by the global median and global MAD across all projects, and heterogeneity was captured as (1) the maximum absolute difference between project-specific medians and (2) the same difference normalized by the global MAD. The latter was the most useful parameter. All prognostic parameters were additionally reviewed manually. For temporal evolution parameters, we used automatic filtering based on the MAD-normalized intermedian difference. At that, values 1–2 indicate possible mild batch imbalance (which can be because of cohort-level biases or mild batch effects that are inherent and inevitable to pathology images and must be tolerated well by robust parameters). Values >2 indicate a clear cohort-dependent shift and were excluded from analysis. During manual review, all the parameters excluded analyze non-normalized pixel values (colors, intensities) responsible for batch effects.

To identify all such parameters, we repurpose our parameter verification SPARK module to an additional parameter analysis module for quick parameter review at scale (available as part of the GitHub repository) and produce a list of parameters that are potentially vulnerable to batch effects and should be implemented with care (Supplementary Table 3).

### Temporal evolution of the tumor, binary risk maps of SPARK parameters

Our hypothesis is that the temporal evolution of a tumor can be inferred from static data (WSIs) using SPARK parameters that effectively capture the morphological layers of subclonal evolution occurring at deeper, molecular–genetic levels (Fig. 6a). To work with such layers, we spatially map our parameters as areas of high or low risk (Fig. 6a) using the following approach. We first take all SPARK parameters associated with aggressiveness from the previous analysis (that is, those significant in univariate Cox analysis as either dichotomized or continuous variables, after decorrelation; Fig. 4). For purity, for each tumor type, we exclude all parameters potentially affected by batch effects in at least one cohort for purity (using the statistical approach described above; threshold). For each parameter (and for each tumor type separately), we determine thresholds using quantiles of parameter values for the entire cohort (Q5–Q95, with steps of 10 between Q10 and Q90). For each Q-cut-off, we then binarize the tumor area in all cohort cases into low-risk (0) and high-risk (1) regions. As our parameters include both positively associated (higher values = more aggressive) and negatively associated (higher values = less aggressive) measures of aggressiveness, we incorporate this directional information from the previous analysis (Fig. 4). In all binary tumor maps, a value of ‘1’ consistently corresponds to high risk, regardless of parameter direction. Next, for each Q-threshold, we perform univariate Cox analysis for the proportion of tumor area in the high-risk category to identify the most significant Q-threshold (based on the *P* value of the univariate analysis). This optimal threshold is then used to binarize all tumor cases and generate high-/low-risk maps for temporal evolution analysis (Fig. 6a). If a parameter was not measurable in the region (the reason is always that the target cell(s) are not available), we uniformly mapped it as low risk (that is, effectively leading to filtering such regions from pairwise effect analysis).

### Temporal evolution of the tumor, global timing

Based on the generated binary maps of pathology features, we assume that high-risk features occupying a larger tumor area appear earlier. We classify all features as early, mid-term or late based on the number of tumors in the cohort exhibiting a specific feature in the corresponding global timing state. For finer granularity, we also define two additional categories—early-mid and mid-late—when the number of tumors in the cohort is roughly the same for the two corresponding categories (Δ < 10 tumors).

### Temporal evolution of the tumor, temporal sequence analysis

The aim of this analysis is to identify pairs of parameters where parameter A precedes parameter B—that is, parameter A creates a field effect necessary for the acquisition of parameter B (Fig. 6a). To establish the temporal relationship between two or more feature layers from binary maps, we use the concepts (filters) of support, directionality, conditional necessity and sufficiency, applying them to each tumor case in the cohort. Most filter values are empirical, chosen to retain the most significant temporal associations while avoiding issues related to multiple hypothesis testing, yet without losing biological relevance.

For two parameters A and B in a tumor, there are four different states concerning overlap of low- and high-risk regions: 00, 01, 10 and 11. We set a moderately conservative support level for *n*_11_ of 30 regions within a single tumor necessary to start analysis for AB pairs.

The directionality filter is intended to identify an asymmetric relationship between parameter: that is, if A precedes B, there should be directional asymmetry for A versus B and not vice versa. We calculate directional ratios: dir_AB = *n*_10_/*n*_01_ (A without B versus B without A) and dir_BA = *n*_01_/*n*_10_. A directional relationship was established if (1) the absolute difference |*n*_01_ − *n*_10_| ≥ 12, ensuring meaningful asymmetry, and (2) either dir_AB > 5 or dir_BA > 5, indicating that one parameter occurs substantially more often without the other. The direction with the higher ratio determined the orientation (A → B or B → A) for subsequent analyses.

Conditional necessity estimates whether A is required whenever B appears: that is, when A happens, whether B usually follows. Conditional necessity was calculated as CN = *n*_1__1_/(*n*_01_ + *n*_11_). We set the CN filter threshold at 0.85 (if CN ≤ 0.85 ⇒ A not necessary for B; if CN > 0.85 ⇒ filter pass).

Sufficiency evaluated whether the presence of parameter A is sufficient to predict the presence of parameter B. Sufficiency was calculated as Suff = *n*_1__1_/(*n*_10_ + *n*_1__1_), representing the proportion of regions with both parameters present among all regions where parameter A is present. We exclude pairs with low sufficiency (Suff < 0.2) due to statistical (multiple hypothesis testing) and biological (uncertain effect) considerations and with high sufficiency (Suff > 0.8), suggesting strong correlation rather than temporal connection.

All pairs that survive all four filters and with sufficiency in range 0.2 ≤ Suff ≤ 0.8 are included in the temporal sequence analysis. In this, we identify chains of three parameters (A → B → C) to decipher the evolutionary sequence of events within the tumor. We consider only chains that occur in >20 tumors and those associated with tumor aggressiveness. For the latter, we use the following approach. For each of the identified chains, we calculate two types of metrics for patient subcohorts with and without this chain: (1) frequency ratio of events, pN1/pN0 and of UICC pathological Stage III–IV / Stage I–II; and (2) relative risk ratio at 36 and 60 months of event for OS, CSS and PFS endpoints. We set rather aggressive filtering to leave only the most significant chains for analysis (1) a pathological variables ratio threshold of at least 5.0 and (2) a relative risk ratio threshold of at least 2.0. We include chains in the downstream analysis if at least two of any ratios are over the threshold for all tumors except colorectal cancer, where, similar to prognostic analysis, the number of significant parameters is very high and chains with >4 significant parameters are taken into consideration.

### Statistics and reproducibility

No statistical method was used to predetermine sample size (all available patients were included). No data were excluded from the analyses. The experiments were not randomized. The investigators were not blinded to allocation during experiments and outcome assessment.

### Software

Python 3.9 or later was used for most experiments. For development of agentic workflows, we used an open-source framework (crewai v.0.86.0) and the crewai-tools package (v.0.17.0), with disabled telemetry mode. For operations on GeoJSONs and processing of polygons, the following libraries were used: scipy (v.1.15.2), rasterio (v.1.4.3), shapely (v.2.1.1), rtree (v.1.4.0), py-opencv (v.4.11.0) and numpy (v.2.2.5). Seaborn (v.0.13.2) and matplotlib (v.3.9.2) were used for plotting. Lifelines (v.0.30.0) was used for Kaplan–Meier analysis. Pandas (v.2.2.3) was used for management of databases. Networkx (v.3.4.2) was used for decorrelation. Scikit-learn (v.1.6.1) was used for dataset management and metrics calculation in predictive modeling.

### Hardware

Development and implementation of the SPARK agentic workflow was performed on a consumer-grade MacBook Pro (M1 Ultra, RAM 64 Gb). Analysis of cohorts (quality control, segmentation, single cell analysis, filtering, SPARK parameter generation) was performed in parallelized mode on an AI server equipped with 4× NVIDIA A100 80 Gb and using the RAMSES HPC of University Hospital Cologne (multiple NVIDIA H100 92 Gb). For our large cohort of WSIs, parameters were evaluated on the RAMSES HPC, with 120 GB and 12 CPUs reserved per case. Predictive modeling and temporal evolution analysis were performed using above-mentioned MacBook Pro.

### Reporting summary

Further information on research design is available in the Nature Portfolio Reporting Summary linked to this article.

## Online content

Any methods, additional references, Nature Portfolio reporting summaries, source data, extended data, supplementary information, acknowledgements, peer review information; details of author contributions and competing interests; and statements of data and code availability are available at 10.1038/s41591-026-04357-y.

## Supplementary information

Supplementary Information Supplementary Figs. 1–12

Reporting Summary

Peer Review File

Supplementary Table 1–4 Tables 1–4

## Data Availability

The TCGA cohorts and associated clinical information are freely available from https://portal.gdc.cancer.gov/. The PLCO and NLST cohorts and associated clinical information are freely available per official request from https://cdas.cancer.gov/. The UKK, UKE, HAL and University Hospital Giessen cohorts are not publicly available due to local regulation and terms of ethical approval. Clinical and pathology data are available for these cohorts (subject for institutional approval of data sharing). All inquiries should be sent to the corresponding author (typical timeline for response, 1–2 weeks).

## Code availability

The code of the whole agentic pipeline and all coded parameters are available via Zenodo at https://zenodo.org/records/18047852 (ref. ^52^) and via GitHub at https://github.com/cpath-ukk/SPARK.
