# Supplementary material for: An agentic framework for autonomous scientific discovery in cancer pathology
Source: Nat Med. 2026 Apr 29;32(6):2254–66. doi: 10.1038/s41591-026-04357-y (PMC13278948; doi:10.1038/s41591-026-04357-y)
Supplement: Supplementary file 1 — Supplementary Figs. 1–12 [file 41591_2026_4357_MOESM1_ESM.pdf]

nature medicine

Supplementary information

https://doi.org/10.1038/s41591-026-04357-y

An agentic framework for autonomous scientific discovery in cancer pathology

In the format provided by the authors and unedited

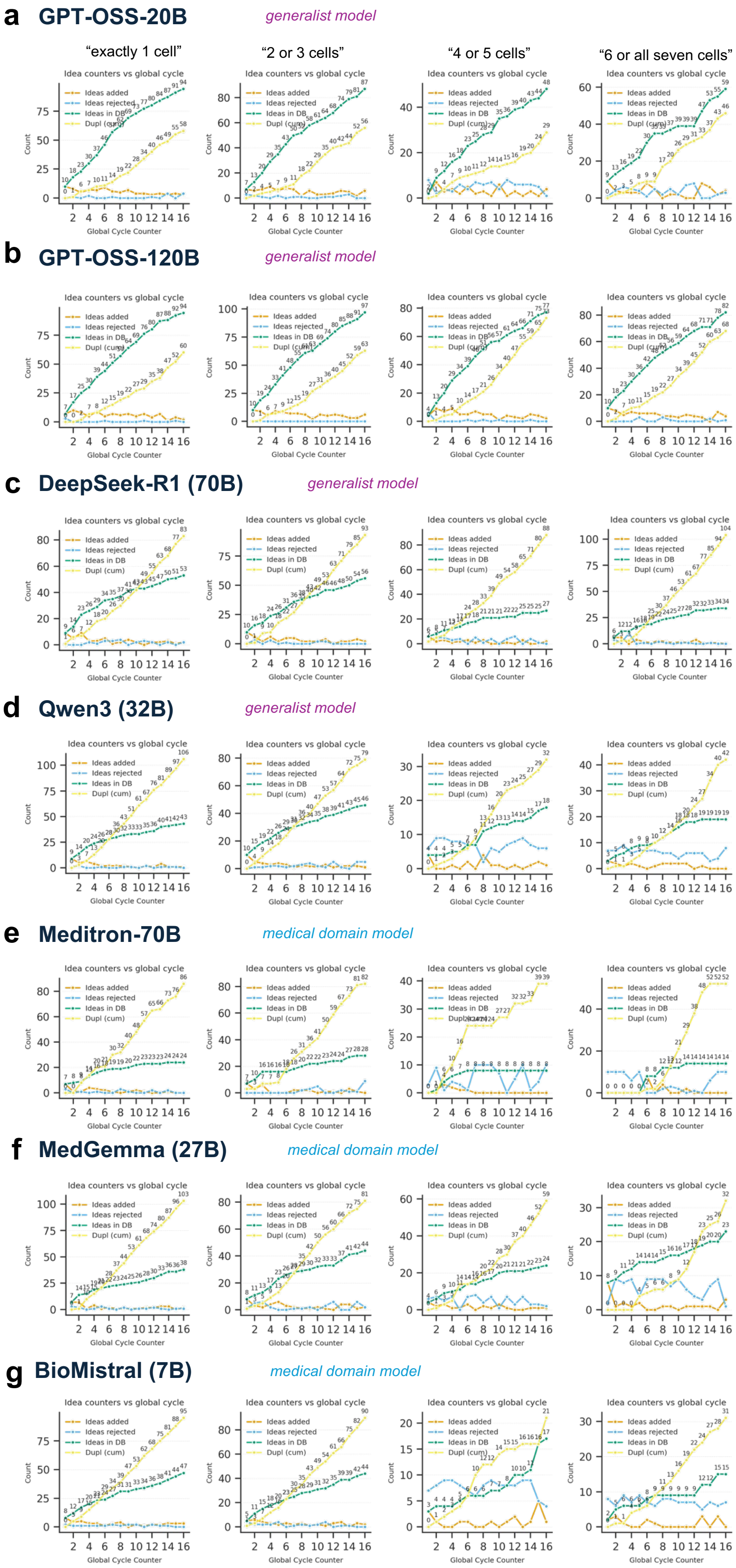

Suppl. Fig. 1 | Using open-source models for idea generation: statistics of idea generation
The Figure presents the results of idea generation pipeline (same setup as in initial experiments) for four generalist (gpt-oss-20b, gpt-oss-120b, distilled DeepSeekR1-LLama3-70B, and Qwen3-32B) and three medical (Meditron-70B, MedGemma-27B, and Biomistral-7B) large-language models.

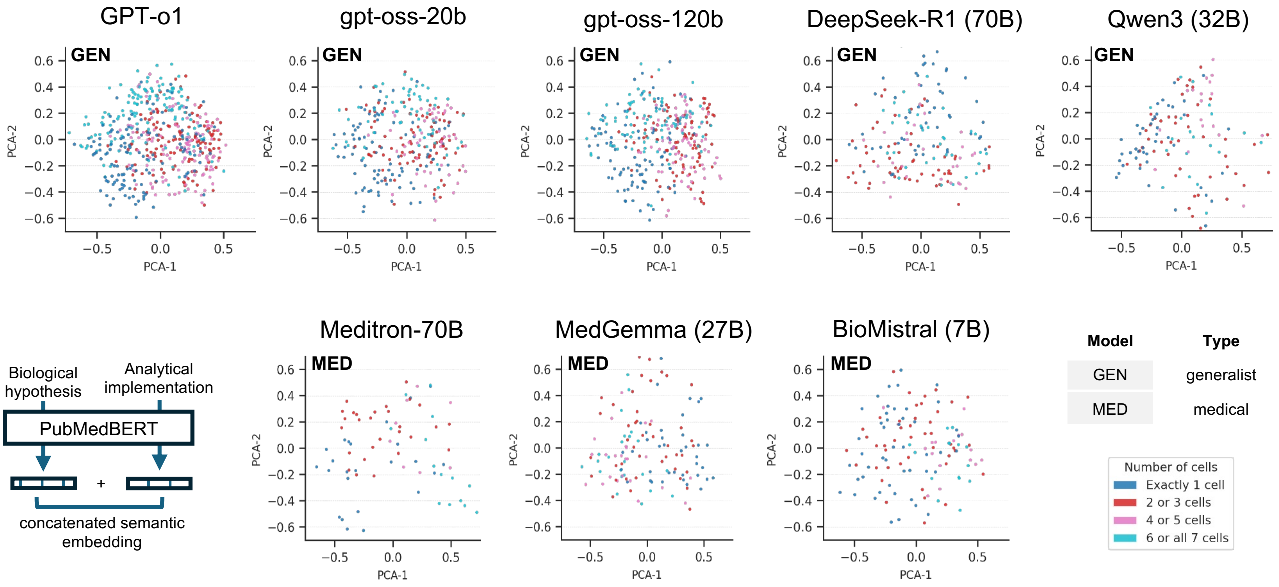

Suppl. Fig. 2 | Using open-source models for idea generation: semantic analysis.
The Figure presents the semantic analysis of ideas (experiments in Suppl. Fig. 1) for four generalist (gpt-oss-20b, gpt-oss-120b, distilled DeepSeekR1-LLama3-70B, and Qwen3-32B) and three medical (Meditron-70B, MedGemma-27B, and Biomistral-7B) large-language models. For comparison also results of o1 model from main experiments are provided.

a DeepSeek-R1 (70B); "2 or 3" cells

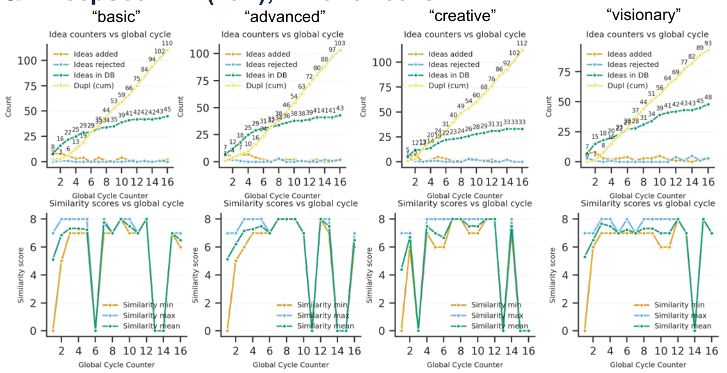

b DeepSeek-R1 (70B); "6 or all seven" cells

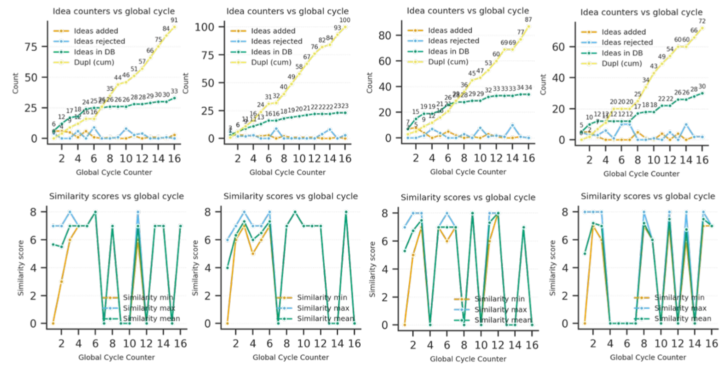

c GPT-OSS-120B; "2 or 3" cells

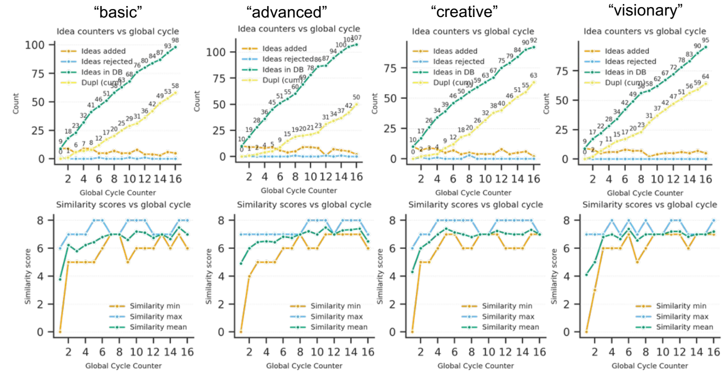

d GPT-OSS-120B; "6 or all seven" cells

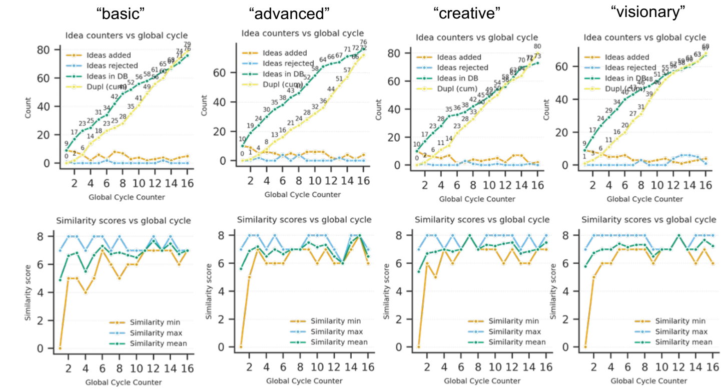

Suppl. Fig. 3 | Using open-source models for idea generation: creativity level
The plots present the idea generation statistics of two different models tested on a selective number of distinct cell types involved into idea ("2 or 3" or "6 or all seven"). The same idea quality/creativity conditions are applied across all 16 cycles of idea generation. From left to right, the four columns represent different idea quality levels: "basic," "advanced," "creative," and "visionary, out-of-the-box". For semantic analysis see Suppl. Fig. 4a-d.

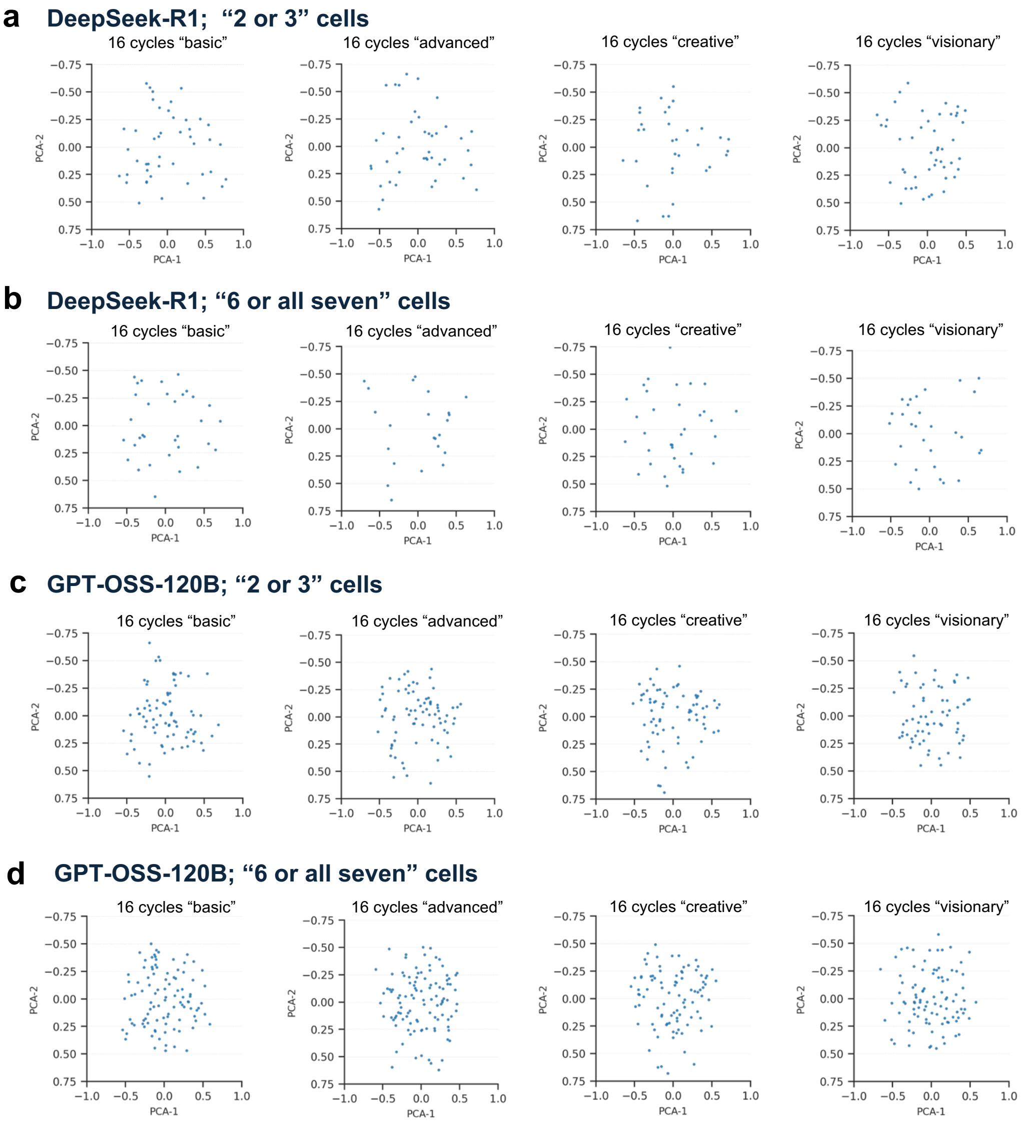

e **Use Case 1: Coding of parameters as functions**

Workflow stage	gpt-oss-120b, n	DeepSeek R1 (70B), n
Ideas generated (original)	500	500
Python code generated	500 (100%)	500 (100%)
Python code executed without errors	495 (99%)	236* (47.2%)
Passed time test (60 s / small test case)	495 (99%)	224 (44.8%)
Final filtering	463 (92.6%)	168 (33.6%)

*Mostly simple errors: syntaxis, missing quotes for comments, unimported packages (can be addressed through tightening of task description)

Suppl. Fig. 4 | Using open-source models for idea generation (creativity level) and coding of parameters
a-d: The plots present semantic similarity scores of two different models tested on a selective number of distinct cell types involved into idea ("2 or 3" or "6 or all seven"). The same idea quality/creativity conditions are applied across all 16 cycles of idea generation. From left to right, the four columns represent different idea quality levels: "basic," "advanced," "creative," and "visionary, out-of-the-box" (data from experiments in Suppl. Fig. 3). **e**: Statistics of parameter coding for two open-source models: gpt-oss-120b and dilated DeepSeekR1-Llama3-70B. The parameters used are from initial experiments (Use case 1 and 2).

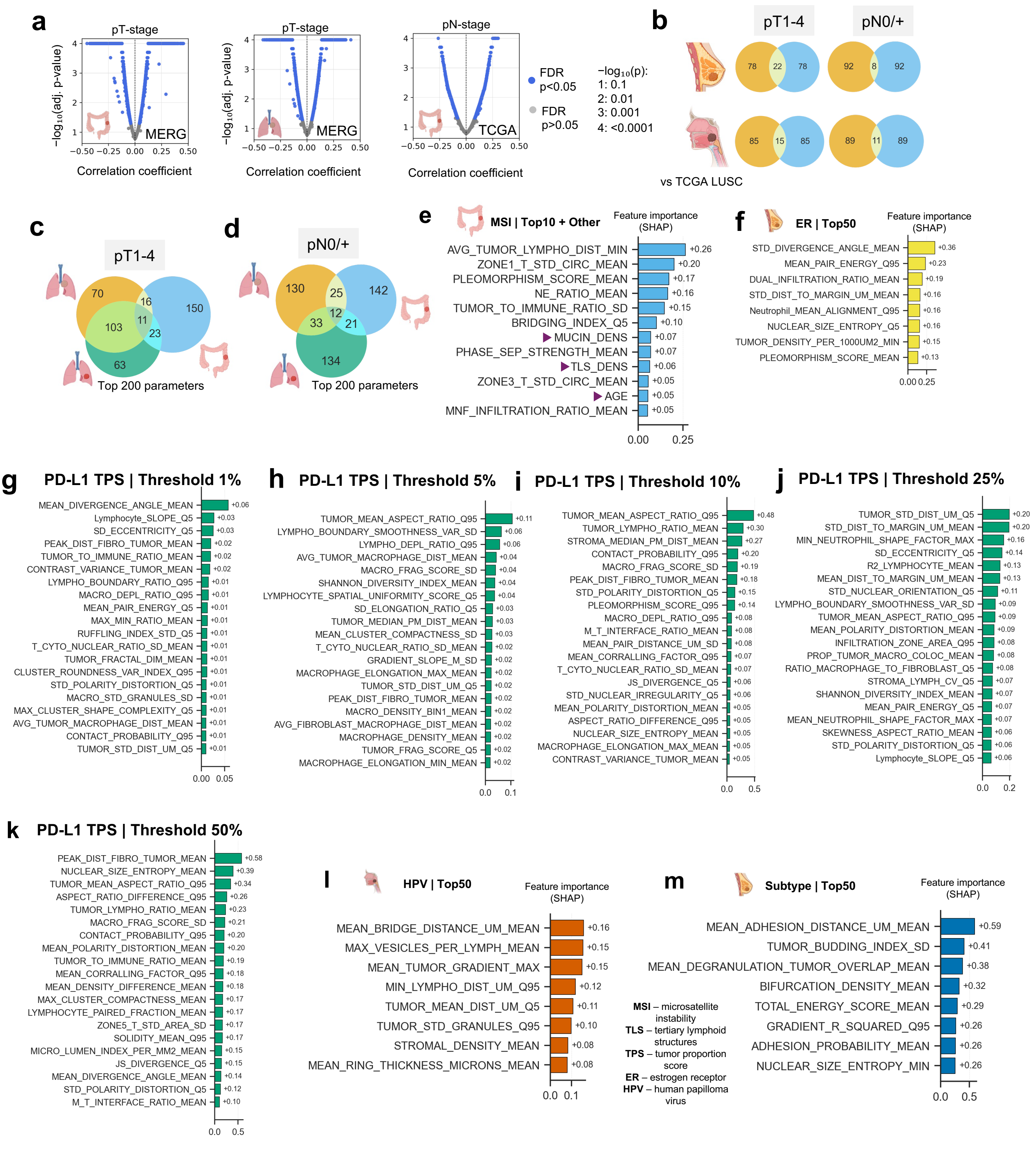

Suppl. Fig. 5 | Correlation analysis and analysis of predictive value of SPARK parameters

a: Correlation of SPARK parameters with pathological variables and predictive biomarkers in single cohorts (Spearman correlation; after FDR correction; two-side). MERG are merged test cohorts analyzed for respective tumor type (details in Table S1). **b:** Analysis of overlaps in parameters with high correlation for exploration and test cohorts (BRCA and HNSC). **c,d:** Analysis of overlaps for three different tumor types - LUAD, LUSC, and COAD (all cohorts: TCGA) - for the top 200 parameters (top 100 with positive correlation and top 100 with negative correlation) correlated to respective clinical parameter. **e-m**: SHAP analysis of feature importance for the models presented in Extended Data Fig. 2k (**e**), Fig. 3g (**f**), Fig. 3h (**g-k**), Fig. 3j (**l**), and Fig. 3f (**m**). **g-k:** SHAP analysis of feature importance for PD-L1 LUAD models trained at different PD-L1 tumor proportion score (TPS) threshold. Partially created in BioRender.

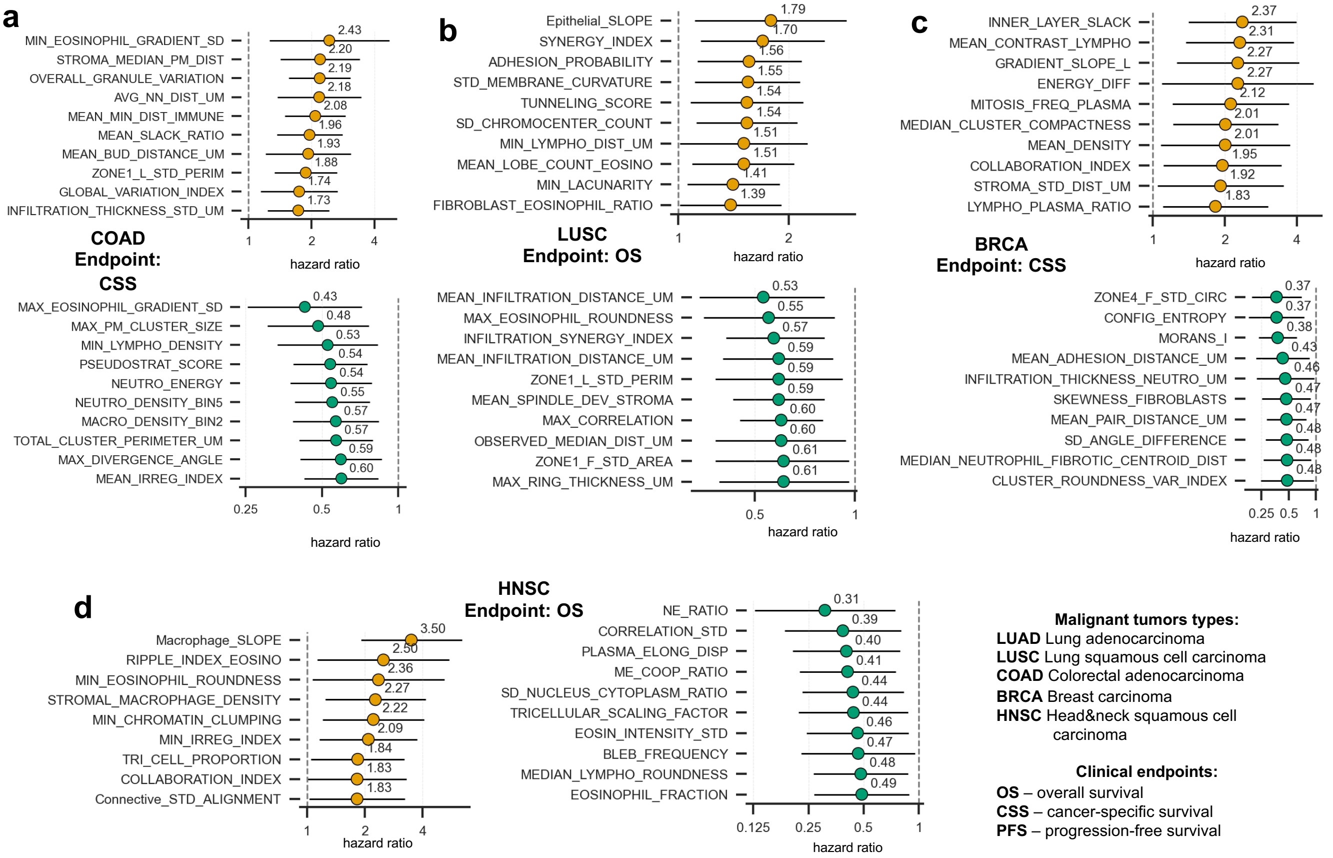

Suppl. Fig. 6 | Prognostic value of SPARK parameters

a-d: Top parameters with positive (orange dots, hazard ratio/HR>1.0) and negative (green dots, HR<1.0) significant prognostic associations in different tumor types, from independent validation in test cohorts (for number of patients/events see Table S1). Error bars are 95% confidence interval for HR.

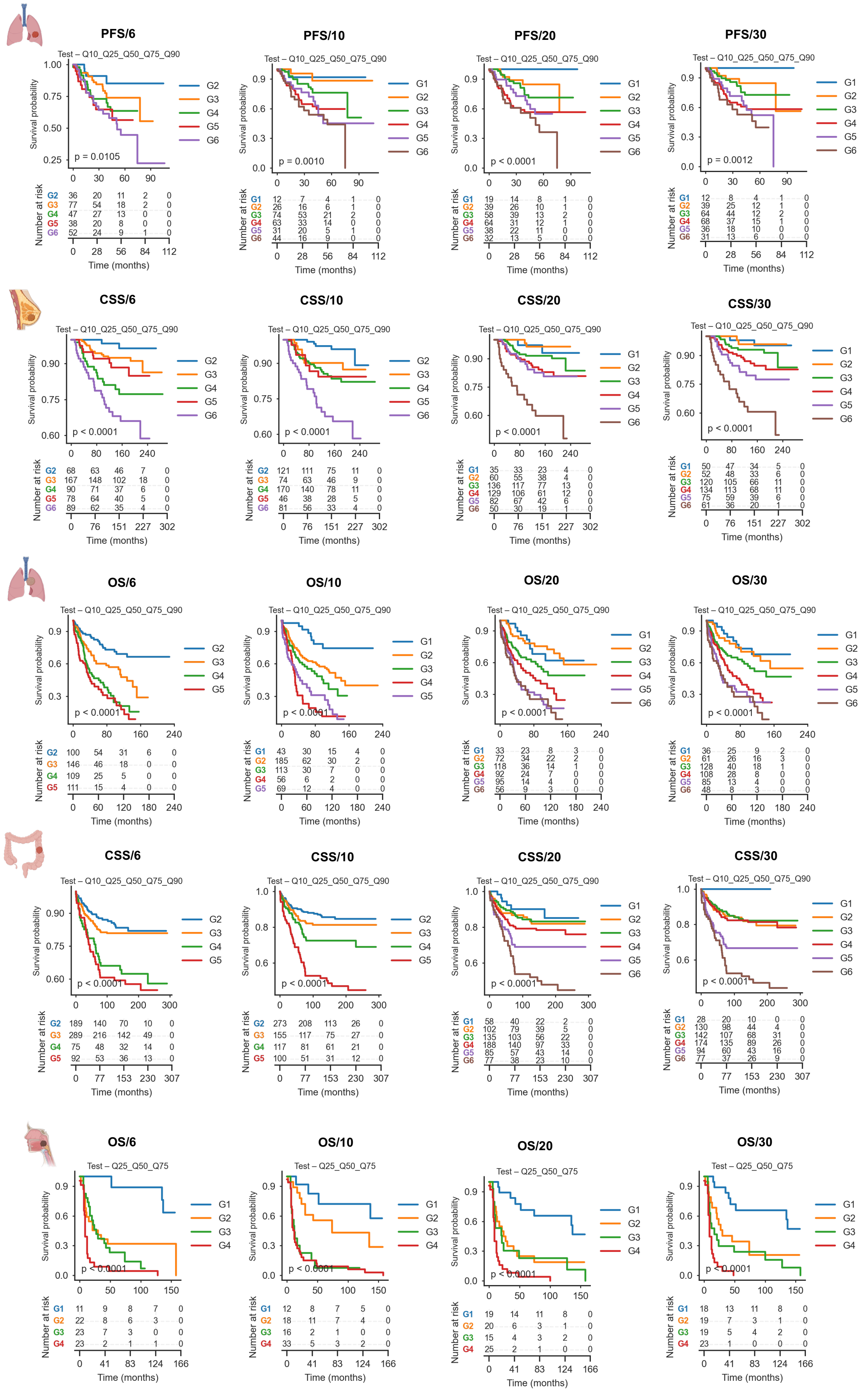

Suppl. Fig. 7 | Multiparameteric prognostic systems.
Further analysis corresponding to Fig. 4j and Extended Data Fig. 4g-n. Two types of risk stratification are used: quantiles: four prognostic groups – quantiles Q25, Q50, and Q75 for prognostic scores as cut-offs, and six prognostic groups - quantiles Q10, Q25, Q50, Q75, Q90 as cut-offs. Clinical endpoints: OS – overall survival, CSS – cancer-specific survival, PFS – progression-free survival. Number after endpoint abbreviation is number of Top SPARK features used for prognostic scoring. Kaplan-Meier Scores with log-rank test. Partially created in BioRender.

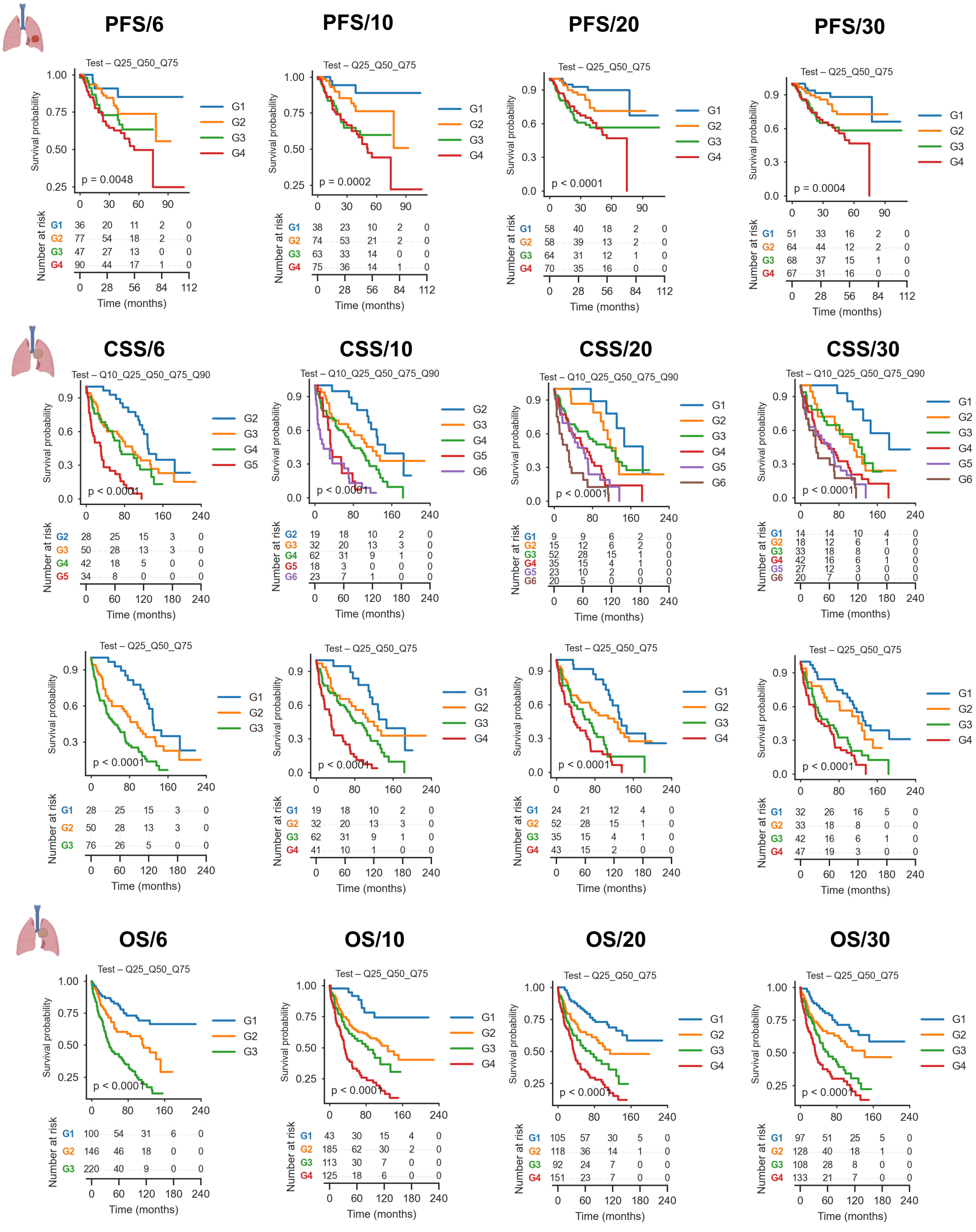

Suppl. Fig. 8 | Multiparameteric prognostic systems.
Extended analysis corresponding to Fig. 4j and Extended Data Fig. 3g-n. Two types of risk stratification are used: quantiles: four prognostic groups – quantiles Q25, Q50, and Q75 for prognostic scores as cut-offs, and six prognostic groups - quantiles Q10, Q25, Q50, Q75, Q90 as cut-offs. Clinical endpoints: OS – overall survival, CSS – cancer-specific survival, PFS – progression-free survival. Number after endpoint abbreviation is number of Top SPARK features used for prognostic scoring. Kaplan-Meier Scores with log-rank test. Partially created in BioRender.

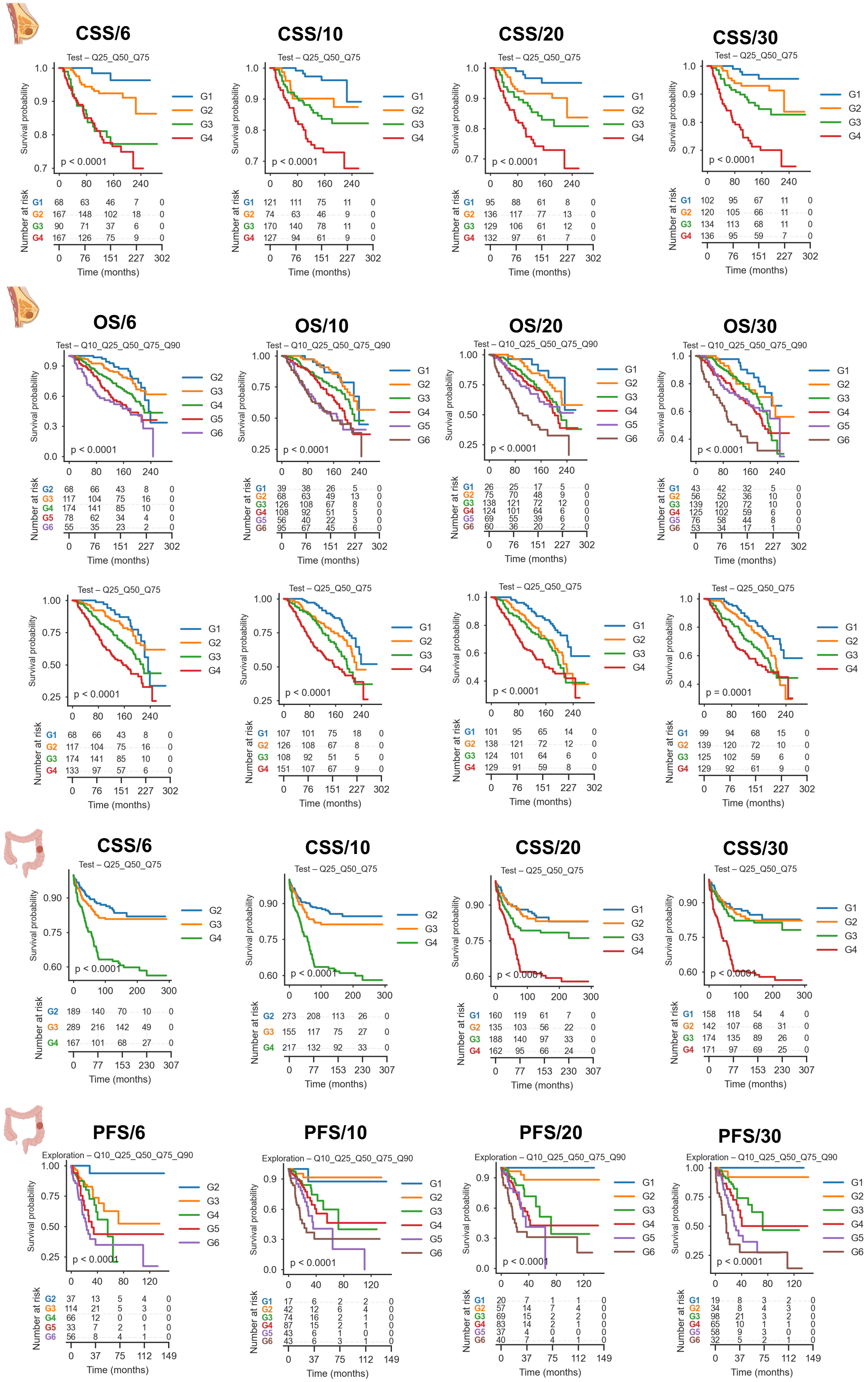

Suppl. Fig. 9 | Multiparameteric prognostic systems.
Extended analysis corresponding to Fig. 4j and Extended Data Fig. 3g-n. Two types of risk stratification are used: quantiles: four prognostic groups – quantiles Q25, Q50, and Q75 for prognostic scores as cut-offs, and six prognostic groups - quantiles Q10, Q25, Q50, Q75, Q90 as cut-offs. Clinical endpoints: OS – overall survival, CSS – cancer-specific survival, PFS – progression-free survival. Number after endpoint abbreviation is number of Top SPARK features used for prognostic scoring. Kaplan-Meier Scores with log-rank test. Partially created in BioRender.

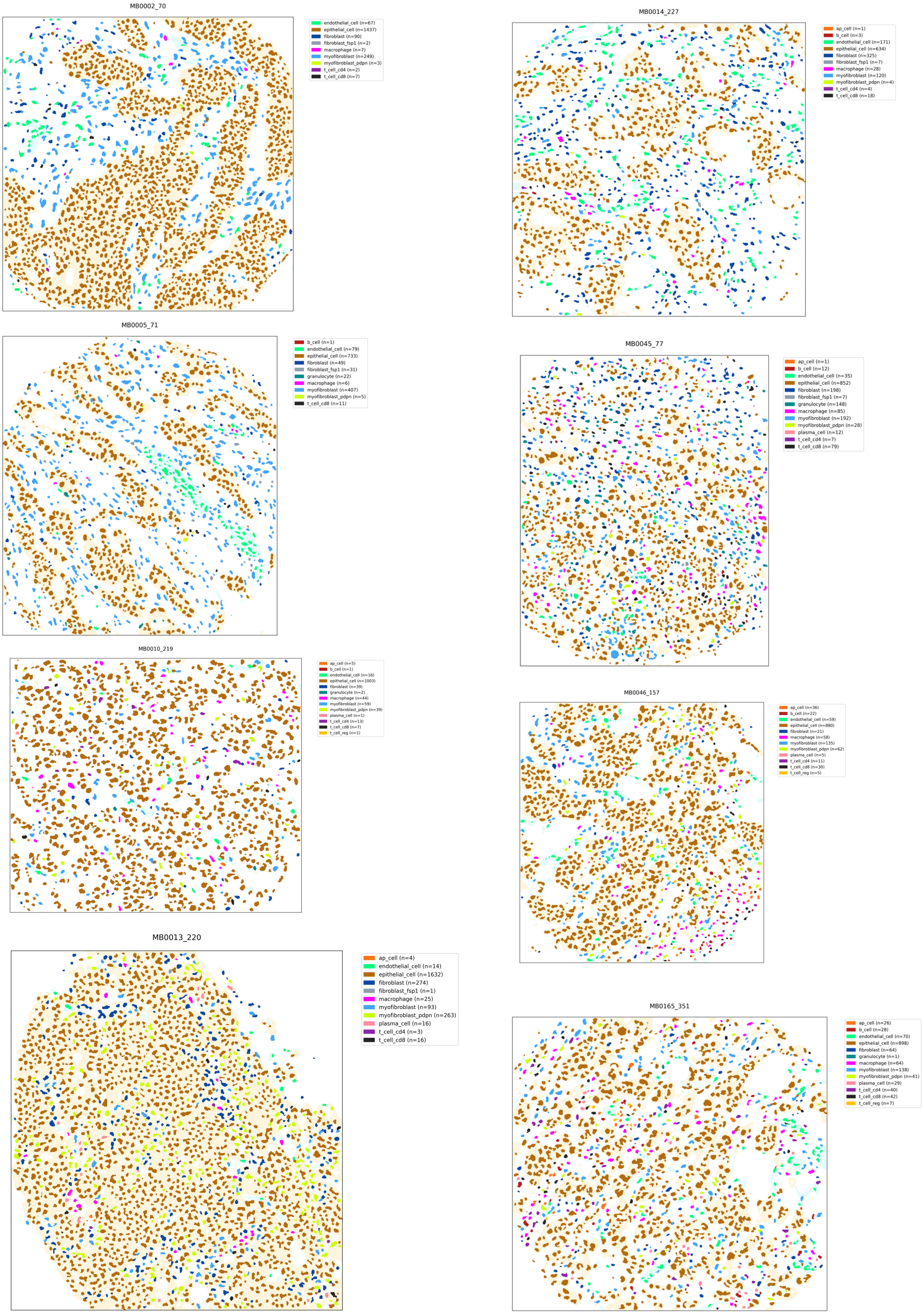

Suppl. Fig. 10 | Examples of tumor samples from METABRIC cohort.

Provided are quantifications of different cell types in single tissue microarray cores.

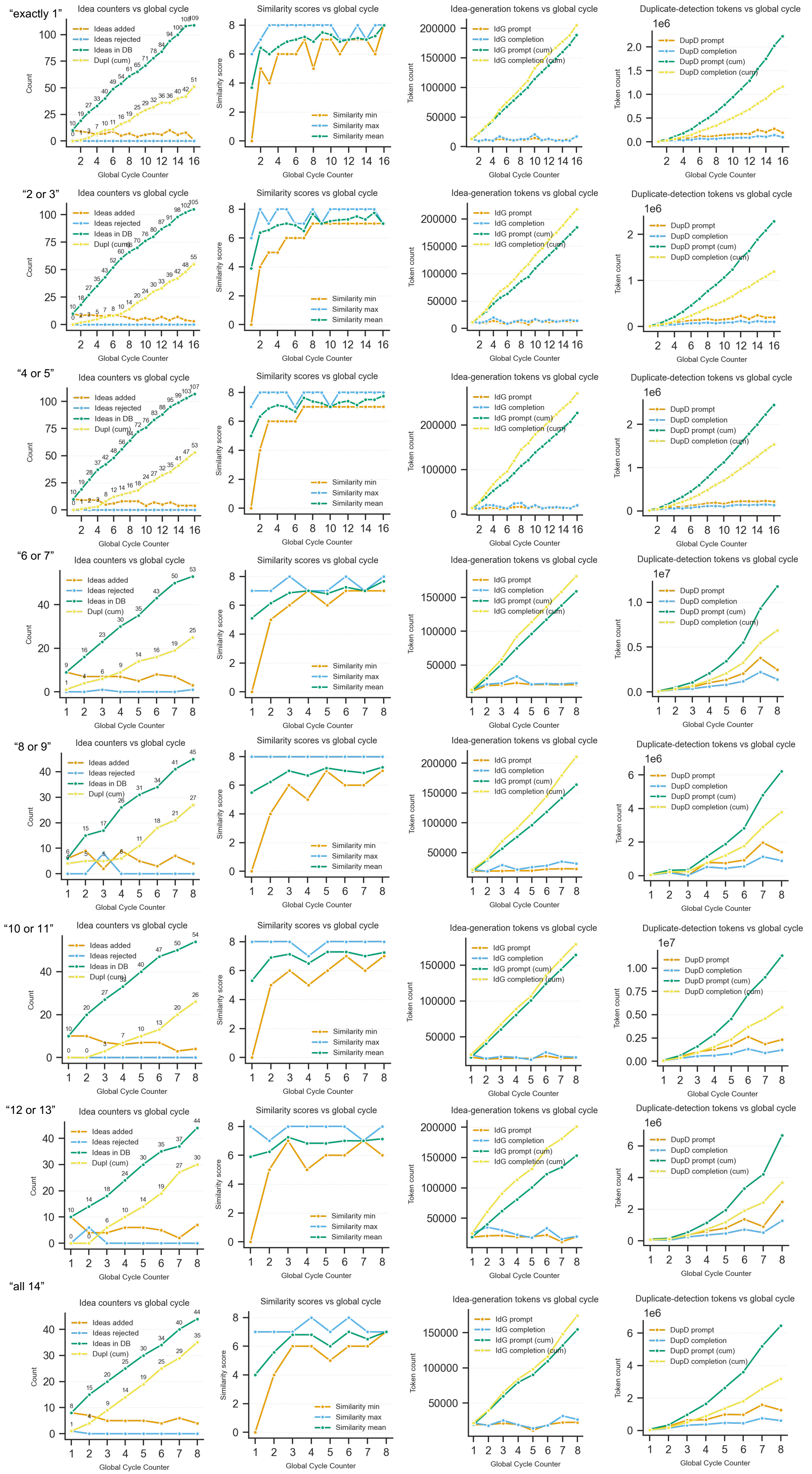

Suppl. Fig. 11 | SPARK: Idea generation for Use case 3.

Metrics of idea generation per run. From left to right: 1) number of original, duplicate, and rejected ideas during the generation process, 2) similarity scores of ideas during generation cycles, showing increasing trends in later cycles, indicating reduced capacity for original ideas, 3) token consumption for different parts of the idea generation steps, 4) token consumption for different parts of the duplicate detection step, 5) time spent on the full idea generation process per run.

a

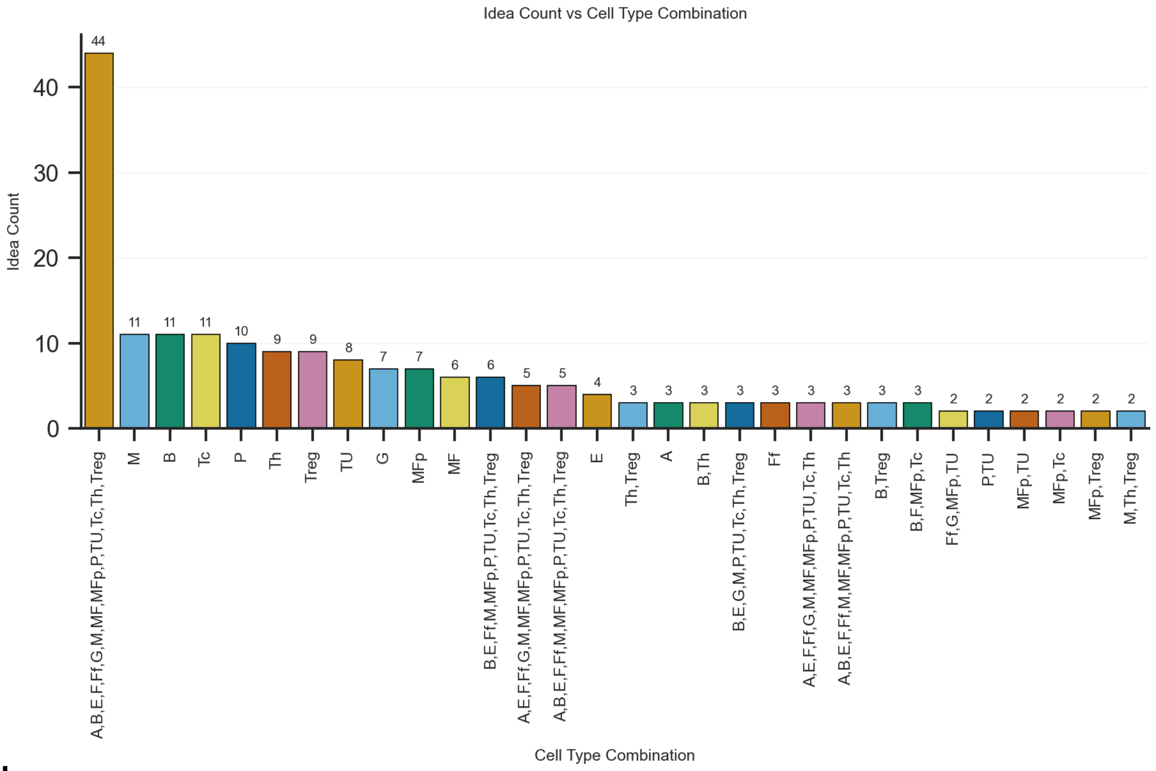

b

Parameter	CSS_ HR	CSS_ P	CSS_CI_ LOW	CSS_CI_ HIGH	CSS_CINDEX_ BASE	CSS_CINDEX _FULL	CSS_CINDEX_ DELTA	CSS_LRT_ P
i3ew4evg5a4d_M_proportion	2.64	0.0004	1.53	4.53	0.50	0.59	0.09	0.0011
9i7j2li19588_TU_norm_boundary_stroma	1.98	0.0023	1.28	3.08	0.50	0.59	0.09	0.0013
td29c04foc15_TU_to_Tc_ratio	1.88	0.0027	1.25	2.85	0.50	0.59	0.09	0.0022
du70lswfg3ei_M_percent	1.79	0.0036	1.21	2.65	0.50	0.57	0.07	0.0030
yldxliwbr70c_weighted_dist_G	1.74	0.0039	1.19	2.53	0.50	0.57	0.07	0.0043
rxnm2a3j57a0_M_MEDIAN_PACKING_FACTOR	1.90	0.0047	1.22	2.96	0.50	0.57	0.07	0.0030
fyyse1b272ey_FIBROBLAST_TO_MACROPHAGE_RATIO	0.60	0.0075	0.41	0.87	0.50	0.57	0.07	0.0081
pvush9sb1jbe_STD_NUCLEAR_SOLIDITY_PDPN+ Myofibroblast_Epithelial	2.50	0.0080	1.27	4.92	0.50	0.61	0.11	0.0064
vkzlzyx082c4_stroma_fibroblast_to_epithelial_cell_ratio	0.57	0.0083	0.38	0.87	0.50	0.58	0.08	0.0064
jl5tb6agvujt_E_aggregate_deformation_score	0.52	0.0108	0.32	0.86	0.50	0.56	0.06	0.0064
crecbc67jue4_corr_M_MF	1.65	0.0113	1.12	2.42	0.50	0.56	0.06	0.0127
s3gkpz5i725j_tumor_cell_proportion	1.92	0.0113	1.16	3.19	0.50	0.56	0.06	0.0066
3lpt42dyr5yq_global_immuno_fibroblastic_infiltration_index	1.69	0.0123	1.12	2.54	0.50	0.57	0.07	0.0099
o37ukl0ly6vh_G_TH_RATIO	1.82	0.0149	1.12	2.93	0.50	0.53	0.03	0.0212
aav60os6mfai_TRIANGULATION_IRREGULARITY_INDEX	0.45	0.0150	0.23	0.85	0.50	0.55	0.05	0.0067
5c2cnux8cz2e_composite_K_50	1.64	0.0154	1.10	2.44	0.50	0.56	0.06	0.0186
hpbm5qpwotvf_TRIPLET_E-E-Ff_FREQ	0.46	0.0165	0.25	0.87	0.50	0.59	0.09	0.0235
2uydkrgg0ths_G_proportion	1.60	0.0174	1.09	2.35	0.50	0.56	0.06	0.0201
bth0xsp46xys_Treg_APC_ratio	1.56	0.0234	1.06	2.30	0.50	0.56	0.06	0.0232
i3ew4evg5a4d_A_proportion	1.79	0.0261	1.07	3.00	0.50	0.57	0.07	0.0303
yldxliwbr70c_rel_abundance_M	1.54	0.0278	1.05	2.25	0.50	0.55	0.05	0.0303
jwpurc1xc2xt_MEAN_COMPACTNESS_MF	0.63	0.0289	0.41	0.95	0.50	0.53	0.03	0.0351
144ijpego00z_overall_interaction_score	0.66	0.0328	0.45	0.97	0.50	0.56	0.06	0.0319
m8693e5z9j3h_Fraction_F_within_50um_of_TU	0.62	0.0344	0.39	0.96	0.50	0.56	0.06	0.0276
9d00z9xtifzo_F_density_180_200	0.63	0.0356	0.41	0.97	0.50	0.56	0.06	0.0299
mc5dli1azo3k_TU_perimeter_velocity	1.59	0.0373	1.03	2.45	0.50	0.55	0.05	0.0308
1v6365tdzyws_myofibroblast_pdpn_adjacency_ratio	1.64	0.0401	1.02	2.64	0.50	0.55	0.05	0.0321
9mp42ayyvnj7_Th_correlation_coefficient	1.71	0.0402	1.02	2.87	0.50	0.55	0.05	0.0321
7a3yg8qpno81_COMBINED_STROMAL_FD_INDEX	1.47	0.0484	1.00	2.16	0.50	0.55	0.05	0.0522
pbyvmdvdrh87_mean_angle_consistency	0.63	0.0486	0.40	1.00	0.50	0.55	0.05	0.0400

Suppl. Fig. 12 | SPARK: Idea generation statistics and validation of Use case 3.

a: Use case 3: statistics of output. Plot shows the number of ideas based on analysis of certain cell type combinations.
b: Thirty independently validated parameters in Use Case 3 (cross-validation, corresponding to Extended Data Fig. e-h)
