# Supplementary material for: An agentic framework for autonomous scientific discovery in cancer pathology
Source: Nat Med. 2026 Apr 29;32(6):2254–66. doi: 10.1038/s41591-026-04357-y (PMC13278948; doi:10.1038/s41591-026-04357-y)
Supplement: Supplementary file 2 — Reporting Summary [file 41591_2026_4357_MOESM2_ESM.pdf]

nature portfolio

Corresponding author(s): Yuri Tolkach

Last updated by author(s): Sep 12, 2025

Reporting Summary

Nature Portfolio wishes to improve the reproducibility of the work that we publish. This form provides structure for consistency and transparency in reporting. For further information on Nature Portfolio policies, see our Editorial Policies and the Editorial Policy Checklist.

Statistics

For all statistical analyses, confirm that the following items are present in the figure legend, table legend, main text, or Methods section.

n/a	Confirmed	
☐	☒	The exact sample size (*n*) for each experimental group/condition, given as a discrete number and unit of measurement
☐	☒	A statement on whether measurements were taken from distinct samples or whether the same sample was measured repeatedly
☐	☒	The statistical test(s) used AND whether they are one- or two-sided<br>*Only common tests should be described solely by name; describe more complex techniques in the Methods section.*
☐	☒	A description of all covariates tested
☐	☒	A description of any assumptions or corrections, such as tests of normality and adjustment for multiple comparisons
☐	☒	A full description of the statistical parameters including central tendency (e.g. means) or other basic estimates (e.g. regression coefficient) AND variation (e.g. standard deviation) or associated estimates of uncertainty (e.g. confidence intervals)
☒	☐	For null hypothesis testing, the test statistic (e.g. *F*, *t*, *r*) with confidence intervals, effect sizes, degrees of freedom and *P* value noted<br>*Give P values as exact values whenever suitable.*
☒	☐	For Bayesian analysis, information on the choice of priors and Markov chain Monte Carlo settings
☒	☐	For hierarchical and complex designs, identification of the appropriate level for tests and full reporting of outcomes
☐	☒	Estimates of effect sizes (e.g. Cohen's *d*, Pearson's *r*), indicating how they were calculated

Our web collection on statistics for biologists contains articles on many of the points above.

Software and code

Policy information about availability of computer code

Data collection	Raw data collection (whole-slide images, clinical data) did not involve any software. For data preparation (e.g., tissue, cell segmentation) and preprocessing steps, customly created analytical pipelines were used using Python 3.9-3.11. The code is provided as supplement. GrandQC tool (https://github.com/cpath-ukk/grandqc) was used for quality control of digital images.
Data analysis	Large language models: OpenAI: o1, o3-mini; Anthropic: Claude Sonnet 3.5. Hovernext algorithm for single cell detection and classification (was used to train the models on own dataset), v. 1.0. Python 3.9 or later was used for most experiments. For development of agentic workflows we used open-source framework (crewai v. 0.86.0) and crewai-tools package (v. 0.17.0), with disabled telemetry mode. For operations on geojsons and processing of polygons, following libraries were used: scipy (v.1.15.2), rasterio (v.1.4.3), shapely (v.2.1.1), rtree (v.1.4.0), py-opencv (v.4.11.0), numpy (v.2.2.5). Seaborn (v. 0.13.2) and matplotlib (v. 3.9.2) were used for plotting. Lifelines (0.30.0) was used for Kaplan-Meier analysis. Pandas (v.2.2.3) was used for management of databases. Networkx (v.3.4.2) was used for decorrelation. Scikit-learn (v.1.6.1) was used for dataset management and metrics calculation in predictive modelling. XGBoost 2.0.0 with Optuna 4.4.0 were used for training of predictive models. The full code is provided as supplement and will be deposited on GitHub upon submission.

For manuscripts utilizing custom algorithms or software that are central to the research but not yet described in published literature, software must be made available to editors and reviewers. We strongly encourage code deposition in a community repository (e.g. GitHub). See the Nature Portfolio guidelines for submitting code & software for further information.

Data

Policy information about availability of data

All manuscripts must include a data availability statement. This statement should provide the following information, where applicable:

- Accession codes, unique identifiers, or web links for publicly available datasets
- A description of any restrictions on data availability
- For clinical datasets or third party data, please ensure that the statement adheres to our policy

TCGA, PLCO, and NLST cohorts are open-source cohorts. The whole-slide images from proprietary cohorts can be acquired from corresponding author on reasonable request. All analysis results, specifically databases with values of generated parameters, list of parameters in prognostic and predictive applications, functions for implementation of parameters, results of temporal evolution analysis will be open-sourced as large library upon publication.

Research involving human participants, their data, or biological material

Policy information about studies with human participants or human data. See also policy information about sex, gender (identity/presentation), and sexual orientation and race, ethnicity and racism.

Reporting on sex and gender	Sex was reported.
Reporting on race, ethnicity, or other socially relevant groupings	N/a
Population characteristics	The study involves 18 different cohorts with all relevant information provided in Table S1.
Recruitment	All cases are retrospective, no active recruitment was involved.
Ethics oversight	TCGA, PLCO and NLST are open-source dataset. Access to PLCO and NLST was applied for via CDAS portal (cdas.cancer.gov). All study steps were performed in accordance with the Declaration of Helsinki. This study was approved by the Ethical committee of the University of Cologne (20-1583), Halle/Cologne/Essen joint 22-1233 (Project FED-PATH/BMBF). Given the retrospective/archive nature of used data, the necessity of obtaining patients' permission was waived by the ethical committee.

Note that full information on the approval of the study protocol must also be provided in the manuscript.

Field-specific reporting

Please select the one below that is the best fit for your research. If you are not sure, read the appropriate sections before making your selection.

☒ Life sciences ☐ Behavioural & social sciences ☐ Ecological, evolutionary & environmental sciences

For a reference copy of the document with all sections, see nature.com/documents/nr-reporting-summary-flat.pdf

Life sciences study design

All studies must disclose on these points even when the disclosure is negative.

Sample size	Given the nature of the study (retrospective cohorts) no special calculation of sample size was performed, all available patient cases were included.
Data exclusions	Exclusion of patient cases was performed only if image data was not available.
Replication	Only bioinformatical methods are involved in this study. All experiments were thoroughly checked and replicated fully at least once to ensure the integrity.
Randomization	N/a
Blinding	N/a

Reporting for specific materials, systems and methods

We require information from authors about some types of materials, experimental systems and methods used in many studies. Here, indicate whether each material, system or method listed is relevant to your study. If you are not sure if a list item applies to your research, read the appropriate section before selecting a response.

Materials & experimental systems

n/a	Involved in the study
☒	☐ Antibodies
☒	☐ Eukaryotic cell lines
☒	☐ Palaeontology and archaeology
☒	☐ Animals and other organisms
☐	☒ Clinical data
☒	☐ Dual use research of concern
☒	☐ Plants

Methods

n/a	Involved in the study
☒	☐ ChIP-seq
☒	☐ Flow cytometry
☒	☐ MRI-based neuroimaging

Clinical data

Policy information about clinical studies

All manuscripts should comply with the ICMJE guidelines for publication of clinical research and a completed CONSORT checklist must be included with all submissions.

Clinical trial registration	N/a
Study protocol	Not prospective/not clinical/interventional study. Exact approach is fully summarized in Materials and Methods.
Data collection	Data collection for proprietary cohorts was performed in a period 2014-2022.
Outcomes	Not directly applicable to the study design. Resulting prognostic and predictive tools are validated using independent data.

Plants

Seed stocks	N/A
Novel plant genotypes	N/A
Authentication	N/A
