# Supplementary material for: An agentic framework for autonomous scientific discovery in cancer pathology
Source: Nat Med. 2026 Apr 29;32(6):2254–66. doi: 10.1038/s41591-026-04357-y (PMC13278948; doi:10.1038/s41591-026-04357-y)
Supplement: Supplementary file 3 — Peer Review File [file 41591_2026_4357_MOESM3_ESM.pdf]

nature portfolio

# Peer Review File

# An agentic framework for autonomous scientific discovery in cancer pathology

Corresponding Author: Dr Yuri Tolkach

**This file contains all reviewer reports in order by version, followed by all author rebuttals in order by version.**

Version 0:

Reviewer comments:

Reviewer #2

(Remarks to the Author)
Major Claims and Novelty
The manuscript introduces SPARK (System of Pathology Agents for Research and Knowledge), an agentic AI framework that leverages language models to autonomously generate, code, and verify biologically meaningful features from histopathology H&E slides and multiplexed spatial-omics images. This represents a novel paradigm, treating natural language as a universal interface for hypothesis generation. Validation across 18 retrospective cohorts and application to spatial biology demonstrate potential breadth. While conceptually original, similar directions have been explored in related works such as CellVoyager, WSI-Agents, MMedAgent-RL, and PathFinder, albeit typically based on foundation models rather than handcrafted features. Thus, the claim is innovative but should be contextualized within this emerging literature.
Strength of the Evidence
The evidence is generally convincing, supported by multi-cohort validation, predictive accuracy for clinical variables, and temporal/spatial analyses. However, there are several limitations:
- The pipeline depends on upstream segmentation and detection models; errors or biases in these models propagate into SPARK.
- Idea evaluation is based on execution speed and non-trivial outputs, potentially discarding biologically valuable but computationally demanding features.
- Novelty is not formally assessed beyond duplicate detection; incremental variations may dominate.
- Prognostic modeling relies heavily on p-values and assumes linear hazard relationships without testing proportional hazards assumptions. Interaction effects, subgroup analyses, and flexible modeling approaches (e.g., splines, time-varying covariates) were not explored.
- Robustness under realistic data noise and missingness was not tested, limiting confidence in real-world applicability.
Impact and Interest
The paper is likely to attract interest across pathology, oncology, and computational biology communities. By bridging human-expert input with AI-driven hypothesis generation, SPARK reduces barriers to testing novel ideas. Until histopathology-specific vision–language models are developed, such agentic workflows will play an important role in scaling exploratory research. The temporal evolution analysis in particular may stimulate new ways of thinking about tumor progression.
Outstanding Features
- Conceptual novelty: using natural language as a universal interface.
- Modular, transparent, and reproducible workflow with commitment to open-source release.
- Demonstrated versatility across prognostic, predictive, spatial, and temporal applications.
- Enables human–AI collaboration by translating free-text ideas into measurable features.
- Competitive predictive performance for biomarkers such as PD-L1 and MSI.
Statistical and Methodological Considerations
Statistical analyses are only partially adequate. The reliance on dichotomization and p-values without effect sizes, C-index, or cross-cohort stability weakens claims of clinical relevance. Proportional hazards assumptions were not tested; Schoenfeld residuals and time-varying covariates should be applied where appropriate. Non-linear effects could be modeled using restricted cubic splines. Error bars and confidence intervals should be clearly defined in figure legends, and discrimination metrics should supplement p-values for feature selection.
Suggestions for Improvement
- Develop or fine-tune domain-specific LLMs to improve biological reasoning and reduce reliance on costly commercial

models.
- Introduce a novelty metric (e.g., semantic distance in embedding space) to prioritize conceptually distinct ideas.
- Allow flexible data structures to accommodate additional modalities and hypotheses beyond the current rigid schema.
- Explore interaction effects and subgroup analyses (e.g., stage-specific or treatment-specific subgroups).
- Assess robustness under noisy or incomplete datasets to approximate real-world conditions.
- Provide clearer narrative descriptions of algorithms in the manuscript and relegate raw variable names to supplementary code repositories.

(Remarks on code availability)

Reviewer #3

(Remarks to the Author)
PREFACE
I am not a pathologist, oncologist, or computer scientist; I likely represent many Nature Medicine readers if this is published. I am a clinician with an interest in AI/LLMs. My comments focus on clarity, clinical relevance, and safeguards (leakage, multiplicity, generalisability). Some of my comments may reflect technical naivety, however this would probably be expected for other readers as well. I defer technical methodology to technical reviewers. I apologize for the length, but it is also an imposing article.

KEY RESULTS
The authors present SPARK (System of Pathology Agents for Research and Knowledge), an agentic framework that: (i) uses language to generate biomarker ideas; (ii) auto-codes them into computable features ("parameters"); and (iii) evaluates them for prognosis/prediction across multiple cohorts/tumour types. They state SPARK requires no training (for parameter generation), scales to hundreds of ideas, and achieves strong—sometimes state-of-the-art—performance. Parts of the system/code will be open-sourced.

VALIDITY
Ambitious scope and impressive scale. Several guardrails should be made explicit for non-specialist readers to assess the claims.

DATA & METHODOLOGY
Leakage guardrails (major point).
Please confirm that threshold choices for dichotomising features, feature pruning/"decorrelation," and any other selection steps were made only in the exploration/training data (or within cross-validation) and then kept fixed for all independent tests. Please state this explicitly for the human-initiated module as well, where it seems ad-hoc changes were made.
FDR
Please specify the set of tests over which FDR was applied (e.g., per tumour type and endpoint, and whether continuous and dichotomised variants were included).
Redundancy at the patient level.
Decorrelation appears to be done at the patch/region level, while outcomes are patient level after aggregation. Please also check and report redundancy after patient-level aggregation, to ensure retained features are not effectively duplicates for prognosis.
Spatial biology section
Given there is no independent test cohort here, please include an internal, leakage-free validation where all tuning/selection is kept separate from evaluation (e.g., a form of cross-validation that isolates tuning from testing) and report how stable features are.
Temporal ordering from static images.
For the temporal claims, please (i) define precisely how "A precedes B" is determined, and (ii) clarify how multiple testing was controlled across the full set of A–B pairs and A–B–C chains. As written. I am unsure if this section is hypothesis generating or meant to test.
"No training."
Please temper phrasing: SPARK may not require additional training to generate parameters, but MSI with XGBoost is trained, and upstream QC/segmentation/cell-typing models are pre-trained which influence results.
Calibration and clinical utility.
Where you present multivariable Cox/predictors, if available please report calibration (e.g., calibration slope/Brier) and decision-curve analyses on external/LOCO cohorts, and quantify improvement over clinical baselines.
Batch / stain / site effects.
I assume slides vary by scanner/stain across centres (TCGA, PLCO, NLST, UKK, UKE, HAL). Please clarify any harmonisation or, if none, provide brief sensitivity analyses to show findings are not driven by site/batch. (Similarly for IMC/TMA batches.)
Segmentation / cell-typing dependencies.
Because downstream features rely on upstream segmentation and cell-typing and the article mentions relabeling to avoid misclassification, is there a check to show the hinge on the relabeling rules?
Patch size & inclusion threshold.
Please briefly justify the 1 $mm^2$ patch and the ≥10% tumour-area rule, and show results are robust to reasonable alternatives.
Creativity levels in idea generation.

It is unclear whether “basic → visionary” materially affects outcomes. Please explain the measurable effect of these levels (e.g., diversity or downstream yield).

ORIGINALITY & SIGNIFICANCE
Elements of the concept seem to exist exist (assistant/agent systems, feature-based pathology, multi-agent slide analysis), but the combination and scale appear novel. Related items for positioning:
Pathology assistants/foundation-model pipelines and multi-agent approaches (e.g., conceptually related systems): https://pmc.ncbi.nlm.nih.gov/articles/PMC11799676/ , https://www.nature.com/articles/s41586-024-07618-3 , https://arxiv.org/html/2506.20964v1
“Autonomous scientist”-style loops with a GPT interface in other domain (protein/materials): https://arxiv.org/abs/2504.19017 (Side note, this is named “Sparks”. Maybe consider renaming to avoid confusion?
The claim “single-cell detection is limited to single parameters by humans” seems incorrect (albeit smaller scale than SPARK): https://pmc.ncbi.nlm.nih.gov/articles/PMC12234899/

Discussion tone
Several phrases read as over-selling for a medical journal audience (“unlimited potential,” “new paradigm,” “exhaustive descriptors,” “fully explainable,” “requires no training”). Please moderate language, especially given reliance on pre-trained perception models and at least one trained downstream predictor.

CLARITY & TERMINOLOGY
For a non-pathologist, non-computer scientist, this article is hard to read for several reasons.
Abbreviations & cohort mapping.
Define abbreviations like BRCA, LUAD, LUSC, COAD, HNSC, TCGA, PLCO, NLST, UKK, UKE, HAL at first mention. Maybe provide a concise cohort legend. There are several undefined abbreviations and general terms that many readers of Nature Medicine may not now, which should be clarified. The review is already long so I ask you to check this. The above is just examples.
“Parameter.”
Example of a term; define parameter explicitly on first use (a computable, numeric image feature derived from an LLM-generated idea; measured on patches/regions; aggregated to the case level).
Data density.
The manuscript is extremely dense (8 main figures but >110 sub-figures; 57 supplementary tables), which makes it hard for non-specialists to navigate. Not sure how to solve this, but maybe consider a hyperlinked parameter book, moving non-essential variants to Supplement—or splitting into focused papers.

CLINICAL UTILITY (editor’s query)
If the safeguards above hold and results generalise with calibration and decision-analytic gain beyond clinical baselines, I can see potential utility for explainable risk stratification and feature discovery across imaging tasks. Scope may be limited by the upstream perception models’ domains, e.g. imaging, but if it can indeed be generalized beyond pathology I could see it being used for exploration of biomarkers in a lot of complex diseases (such as Alzheimers). However, I temper my expectations for this as my understanding is that it will not be generalizable due to being dependent on unstream pre-training. Maybe a technical reviewer can help clarify.

Thank you for the opportunity to review.

(Remarks on code availability)
I think it is more appropriate for a technical reviewer.

Reviewer #4

(Remarks to the Author)
The submitted manuscript proposes SPARK, a foundational and agentic AI framework for autonomous tumor analysis through reasoning, concept generation, implementation, and data manipulation. It aims to address existing limitations in feature extraction, explainability, and fragmented analysis. The study includes 18 patient cohorts across five malignant tumor types.

In my view, the key features of SPARK are:

1.It helps us understand how tumors grow and become more aggressive over time by analyzing pathology images.
2.It autonomously generates concepts with fully explainable parameters.

I recommend the publication of this work, but I have some concerns that should be addressed before acceptance.

Points to address:

• Explain how the generated ideas depend on the model’s embedded medical and healthcare knowledge.
• Discuss how the system handles hallucinations during the idea generation phase
• Clarify whether expert human validation is always required for newly generated concepts, and how this impacts the system’s autonomy.
• Elaborate on how the performance of the entire system is assessed. Is there a need to evaluate each phase separately?

• If an error is detected, is the system capable of identifying the specific module responsible?
• Please expand on the role of XGBoost in the system. As it appears to use both autonomously and human-generated parameters to predict biomarker status, its role is critical. Also, why was XGBoost chosen over other classifiers such as Random Forest or deep neural networks? A comparative discussion would add value.

(Remarks on code availability)
I have checked the code. It is well documented and commented. It includes README file with enough instructions for installing and running the application. However, I did not run it.

Version 1:

Reviewer comments:

Reviewer #2

(Remarks to the Author)
The authors have satisfactorily addressed most of my main concerns. In particular, I appreciate the new comparison between generalist models and dedicated medical models, as well as the authors' rationale for the observed inferiority of the latter. Given the broad and diverse set of experiments, some increase in complexity of the manuscript is understandable. I encourage the authors to make full use of the Supplementary Information to present methodological details and additional analyses so that the main text remains clear and focused while retaining academic rigor.

(Remarks on code availability)

Reviewer #3

(Remarks to the Author)
General Comments First, I thank the authors for their comprehensive response. As a clinical reviewer, I cannot verify the accuracy of the purely technical adjustments; however, I appreciate the thoroughness of the revisions. My previous concerns are mostly alleviated, though I have a few remaining observations:

A. Clarity and Readability The text is significantly improved. I believe my experience reflects that of the average reader, and I could follow the narrative much better this time. The added definitions and the revisions to the text (and black text updates) are helpful. Regarding abbreviations in the figures: while I appreciate the effort, the abbreviations within the figures are somewhat obscured by the sheer volume of subfigures. I appreciate the authors' diligence in addressing clarity, though I remain slightly cautious that my improved understanding might be partly due to my previous deep-dive into the material rather than the text alone. I hope the editors are aware of this limitation in my revision.

B. Formatting Errors: I cannot fully evaluate the manuscript due to rendering errors in the PDF. Page 15 appears to be cut off/missing half the content, and the reference list seems to suffer from the same issue.

C. Organization of Supplementary Data: With 57 Excel sheets and a supplementary PDF containing 20 complex figures, the data is difficult to navigate. I strongly recommend adding a dedicated Table of Contents as the first sheet in the Excel file and the first page of the supplementary PDF.

D. Final Recommendation: The volume of data remains overwhelming, though I acknowledge this may be unavoidable given the scope of the work. I have not read all 57 tables nor all the subfigures in the supplementary - and neither will the average viewer. That's probably okay (as I suppose it is for those interested), but it is very hard to navigate in its current form, which makes it inaccesible, and there's too much to delve into the data whilst you read the article the first time.
I appreciate the data in the main manuscript has been drastically reduced.

Assuming the technical reviewers are satisfied with the system's validation, I recommend publication. Whether this fits Nature Medicine or a domain-specific journal is up to the editors, but I believe the quality and scope are worthy of Nature Medicine. The work is impressive, and its open-source nature makes it a valuable tool. I also see significant potential for expanding this approach to other domains (e.g., radiology, biochemistry) if upstream tools allow. A major differentiator between this work and other AI manuscripts I have reviewed is the robust utilization of agentic AI. Of course - this is all hinging on the evaluation of more technical reviewers.

Thank you to both Nature and the authors.

(Remarks on code availability)

Reviewer #4

(Remarks to the Author)
The authors have addressed my concerns in the revised manuscript, and I recommend the publication of this work.

(Remarks on code availability)

**Open Access** This Peer Review File is licensed under a Creative Commons Attribution 4.0 International License, which permits use, sharing, adaptation, distribution and reproduction in any medium or format, as long as you give appropriate credit to the original author(s) and the source, provide a link to the Creative Commons license, and indicate if changes were made.
In cases where reviewers are anonymous, credit should be given to 'Anonymous Referee' and the source.
The images or other third party material in this Peer Review File are included in the article's Creative Commons license, unless indicated otherwise in a credit line to the material. If material is not included in the article's Creative Commons license and your intended use is not permitted by statutory regulation or exceeds the permitted use, you will need to obtain permission directly from the copyright holder.
To view a copy of this license, visit https://creativecommons.org/licenses/by/4.0/

Dear Editors and Reviewers,

We thank you for the large number of valuable comments that allowed improving our work in many relevant aspects.

We addressed all comments in the revised version of the manuscript and performed many additional experiments and analysis to substantiate the validity and impact of our results.

Please see below our point-by-point responses. We now hope that you will find our manuscript acceptable.

Two additional points:
1. Since most of the revision experiments were performed by the second author (Bide Zhang), we would like to slightly adjust the authorship order: Florian Trost and Bide Zhang should be listed as co–first authors. All co-authors have confirmed this decision.
2. We have also prepared an alpha version of the GitHub repository, which we will release upon final decision (see attached PDF).

Please don't hesitate to contact me if you have any further questions.

Kind regards,

Yuri Tolkach, MD

**Comments from Editors:**

- The study is currently written in a way that is not accessible to a general audience; please revise the text, organization of the data and overall flow of presentation of the work to ensure readability.

**Response:**

**We performed extensive language edits to make the manuscript more accessible to a general audience and reorganized the presentation to improve readability. Because most sections underwent substantial revision, we highlighted the changes made in response to the Editor's and Reviewers' comments in the manuscript additionally in blue.**

- It is unclear in the main text how the model was tested and validated, particularly with regards to the biological and scientific plausibility of the ideas generated by SPARK, please address this point in revision.

**Response:**

**Two aspects relate to this issue: 1) LLM hallucination issue (fully unrealistic suggestions during idea generation) – this is addressed in the discussion in the Page 14. 2) Biological/mechanistic validity of ideas that are seemingly reasonable/plausible. We address this point in the last paragraph of Discussion.**

- Rational needs to be provided for each of the case studies presented including an explanation of the clinical value of each exercise.

**Response:**

**We have provided this information at the end of the first Results subsection, “Overview of SPARK” (page 5).**

- There needs to be more clarity about what are the novel findings generated by use of the model.

**Response:**

**We address this now in paragraphs 1 and 2 of Discussion.**

- Cosidering the clinical readership of Nature Medicine, we feel it is important that the manuscript clarifies how you envision the model being useful from a translational and clinical perspective.

**Response:**

**We address this now in paragraph 3 of Discussion.**

- Figures need to be revised, curated and have their complexity reduced - some figures present dozens of panels, and are nearly impossible to parse. Please re-organize the flow of the data, making use of main figures (max 6, each presenting a limited number of panels), extended data, and if needed additional supplemental materials.

**Response:**

**We moved approximately 50% of information from the main Figures (now reduced to six Figures) to Extended Data Figures.**

## Comments from Reviewers:

## Reviewer #2 (Remarks to the Author):

Major Claims and Novelty
The manuscript introduces SPARK (System of Pathology Agents for Research and Knowledge), an agentic AI framework that leverages language models to autonomously generate, code, and verify biologically meaningful features from histopathology H&E slides and multiplexed spatial-omics images. This represents a novel paradigm, treating natural language as a universal interface for hypothesis generation. Validation across 18 retrospective cohorts and application to spatial biology demonstrate potential breadth. While conceptually original, similar directions have been explored in related works such as CellVoyager, WSI-Agents, MMedAgent-RL, and PathFinder, albeit typically based on foundation models rather than handcrafted features. Thus, the claim is innovative but should be contextualized within this emerging literature.

**Response:**

**We mention these works in the Introduction (Page 4); however, we agree with the Reviewer that they either operate in completely different domains (e.g., CellVoyager—single-cell RNA-seq data) or focus on foundational models (e.g., WSI-Agents, MmedAgent-RL, PathFinder) that are currently limited in terms of reasoning capabilities and are not comparable with SPARK in analytical depth and breadth.**

**We believe that a multimodal approach based on vision–language modeling holds great potential for advancing reasoning; however, this class of models has not yet reached that level of sophistication. Importantly, we consider SPARK's features to provide a universal framework for automatized generation of highly diverse, image- and outcome-grounded reasoning ground truth for vision–language models - a key bottleneck at present. In this regard, SPARK can facilitate rapid progress in the development of vision–language models. We have added this point to the Discussion section.**

Strength of the Evidence
The evidence is generally convincing, supported by multi-cohort validation, predictive accuracy for clinical variables, and temporal/spatial analyses. However, there are several limitations:
- The pipeline depends on upstream segmentation and detection models; errors or biases in these models propagate into SPARK.

**Response:**

**We agree that the quality of the outputs may depend on the quality of the upstream algorithms. However, there are several important considerations in this regard:**

**1) The precision of the algorithms used in our study is very high and has been validated in rigorously designed studies involving both formal/clinical validation and independent real-world datasets - for example, for tissue and artifact detection (GrandQC tool; Weng Z. et al., Nat. Commun. 2024, [doi:10.1038/s41467-024-54769-y]), lung adenocarcinoma and lung squamous cell carcinoma classification (Kludt C. et al., Cell Rep. Med. 2024, [doi:10.1016/j.xcrm.2024.101697]), and colorectal cancer analysis (Griem J. et al., Mod. Pathol. 2023, [doi:10.1016/j.modpat.2023.100327]).**

**We have made the widely used GrandQC tool - an essential preprocessing component - fully open source, along with several datasets containing precise manual annotations for training segmentation models in lung and colorectal cancer. Moreover, new publicly available datasets for model training continue to appear each month, further enhancing the robustness and reproducibility of this research field.**

**2. Many research groups and industry stakeholders (e.g., pharmaceutical and biotechnological companies) are actively developing and using highly capable segmentation tools and cell detection algorithms. Several high-quality datasets and pretrained models for cell detection and classification are publicly available, including Lizard, PanNuke, CellViT, HoverNeXt, and HistoPlus, as well as proprietary solutions from commercial entities such as PathAI and Aignostics.**

**We regard SPARK as a foundational principle for data exploration - not only within academia but also in the broader context of biomedical and pharmaceutical research, where high-precision upstream algorithms are already established. We see particularly strong potential in immuno-oncology, where SPARK could facilitate the discovery of explainable and interpretable biomarkers to support the personalization of patient therapy.**

**3. Recently, research in pathology has clearly shifted toward foundational models, which have attracted significant attention due to the relative ease of building tools based on them. Although these models play an important role as components within more complex algorithms, they still lack key capabilities such as explainability and reasoning, and their limitations—such as training biases that restrict generalization—are likely underestimated (e.g., de Jong E.D. et al., 2025; arXiv:2501.18055). As this becomes increasingly recognized (a point we aim to emphasize through SPARK), greater effort can be directed toward developing segmentation and classification backbones that first parse tissue into interpretable objects, characterize these objects, and subsequently build more complex models upon this structured information. This approach, rather than relying on raw H&E images with their inherently high complexity and information density - even for the most advanced foundational models - may enable the creation of more sophisticated tools and algorithms that can serve as upstream components within the SPARK framework.**

- Idea evaluation is based on execution speed and non-trivial outputs, potentially discarding biologically valuable but computationally demanding features.

**Response:**

**We performed a detailed review of the ideas that did not pass our time-efficiency filter (i.e., those deemed computationally too "expensive"). While we agree with the reviewer that strictly applying such a filter could, in principle, exclude biologically meaningful concepts, our review did not reveal any particularly promising biological ideas among these excluded cases. Most of them followed a common pattern of computationally inefficient manipulations involving cell polygons or clustering approaches.**

**Naturally, the simplest way to mitigate the risk of missing valuable ideas is to either relax the time threshold or to manually or automatically review excluded concepts for potential biological relevance - decisions that should be tailored to the specific needs of SPARK's end users.**

**During the revision process, we also developed an additional SPARK module (in response to Reviewer 3's comment) for the automated identification of features potentially prone to batch effects - particularly those involving color-based analyses without adequate normalization. This module enables a fully automated, LLM-based review of parameters according to user-defined queries, thus minimizing the need for extensive manual inspection.**

**The accompanying GitHub repository and user documentation will include a detailed strategy outlining how to navigate such situations and how to automate parameter review using LLMs.**

- Novelty is not formally assessed beyond duplicate detection; incremental variations may dominate.

**Response:**

**SPARK's modus operandi is an exhaustive search for new ideas and parameters guided by the task definition (e.g., identifying prognostic parameters or biological concepts associated with a specific clinical endpoint). Within this search, incremental variations - provided they are not exact duplicates - are not only acceptable but also desirable, as they enrich the exploration space.**

**The risk of small incremental improvements is mitigated through careful task specification and by reintroducing previously generated ideas from the database to inform subsequent generation cycles. Our review of generated ideas demonstrates substantial diversity, rather than repetitive minor variations of a single concept.**

**Multiple guardrails ensure additional protection against redundant, minimally distinct variations at later stages. Specifically, within each exploration cohort, we apply several filtering steps to identify the most important and diverse ideas:**

**1. Regional-level decorrelation: parameters with a "technical correlation" greater than 0.70, i.e., those effectively measuring the same or highly similar features, are filtered out.**

**2. Case/risk stratification-level decorrelation: parameters that yield highly similar prognostic risk group stratifications (Jaccard score < 0.85) are removed.**

**We consider these thresholds to be relatively stringent, retaining only the most valuable parameters. For prognostic use cases, we then select top parameters to construct multi-parametric prognostic systems, confirming that they are complementary - each capturing distinct aspects of disease aggressiveness. As expected, this approach performs robustly across all tumor types (Fig. 4l-t).**

**However, we agree that a formal evaluation of idea novelty and breadth could be beneficial. To this end, we have introduced a new module within SPARK that enables systematic assessment of idea generation outcomes.**

**In brief, we utilize two fields from the idea description - "biological hypothesis" and "analytical/methodological implementation" - to construct a single semantic feature vector. Each field is embedded using a robust biomedical language model (PubMedBERT; output: 768-dimensional embedding vector). Typically, the biological hypothesis field consists of a single sentence, while the analytical/methodological implementation field comprises one, occasionally two, sentences. Empirically, and in line with findings from Retrieval-Augmented Generation (RAG) studies, one densely informative sentence appears to represent the optimal unit for comparing ideas.**

**Accordingly, we generate two embedding vectors - one for each field - and concatenate them for downstream semantic analysis. Technically, this step is straightforward and can be easily adapted to other encoders or semantic analysis frameworks.**

**Our initial experiments (idea generation using the o1 model) demonstrate a substantial semantic spread among generated ideas, supporting the method's ability to produce diverse outputs (new Extended Data Fig. 1l and presented below).**

**Comparisons with four open-source generalist models and three open-source models fine-tuned for medical domains, are presented and discussed in response to your another comment below.**

# Use Case 1: semantic diversity

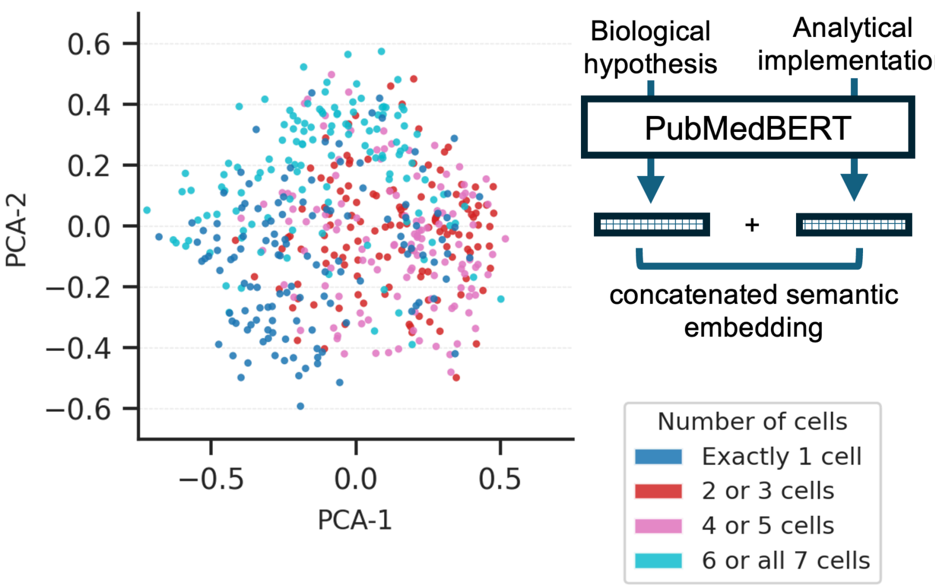


**Extended Data Fig. 1 (<u>new</u>)**

**We also closely examine points that are located near each other, both within the same run (same color, i.e., ideas involving the same number of participating cell types as defined by the task) and across different runs (different colors, corresponding to varying numbers of participating cell types).**

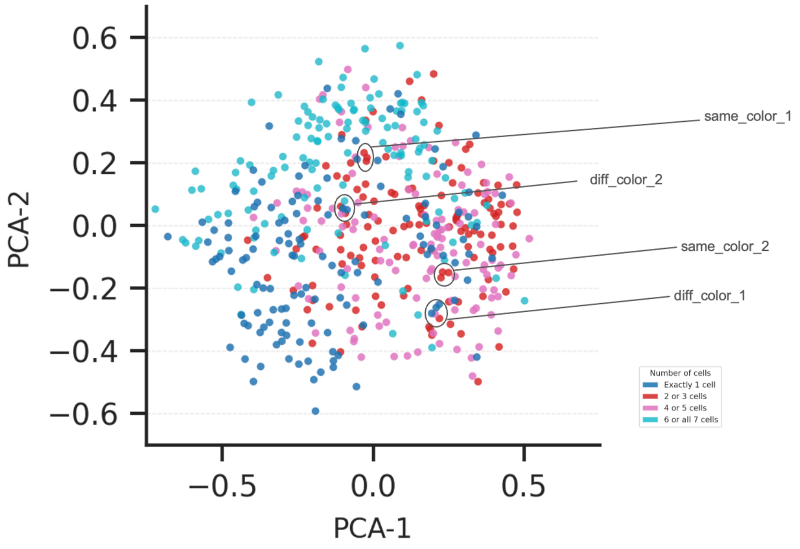


**Although we observe some similarities in the formulation of methodological implementation ideas, none of the closely located points represent virtually identical ideas.**

**#**

**For example, *“same_color_1”* cluster, two most similar ideas:**

**1) "idea_title": "Correlation of tumor and fibroblast nuclear perimeters in stroma-dominated regions",**

**"cancer_type": "multiple cancer types",**
**"cell_type_code": "TF",**
**"biological_hypothesis": "Coupled changes in tumor and fibroblast nuclear perimeter may reflect stroma-driven tumor phenotypes, influencing prognosis.",**
**"analytical_methodological_implementation": "In highly stromal regions, measure tumor and fibroblast nuclear perimeters, compute correlation as the singular morphological parameter."**

**2) "idea_title": "Tricellular infiltration morphological transition measure among tumor cells, lymphocytes, and fibroblasts from central to peripheral tumor compartments",**
**"cancer_type": "multiple cancer types",**
**"cell_type_code": "TLF",**
**"biological_hypothesis": "Gradual morphological transitions among T, L, and F from central to peripheral compartments may mirror evolutionary pathways shaping survival outcomes.",**
**"analytical_methodological_implementation": "Divide tumor into concentric zones, quantify morphological changes in T, L, F infiltration patterns across zones, and correlate transitions with clinical endpoints.",**

**Conclusion: similar cell types, similar formulations, at the end – different parameters.**
**#**

**We observed a moderate correlation (o1: Pearson r 0.575, p-value <0.0001) between semantic distances and our initial duplicate score (range: 0–10), which was assigned by an LLM based on several predefined criteria. However, since we still identify original ideas even within closely clustered regions in the semantic space, we conclude that our duplicate detection approach - based on natural language comparison - performs effectively. That said, this method is less efficient in terms of computational sustainability, as it relies on an LLM-based "one-vs-all" comparison for individual ideas, resulting in increased GPU runtime requirements.**

**<u>This information is summarized now in Extended Data Fig. 1l (new). We also mention this in Results (Page 6).</u>**

- Prognostic modeling relies heavily on p-values and assumes linear hazard relationships without testing proportional hazards assumptions. Interaction effects, subgroup analyses, and flexible modeling approaches (e.g., splines, time-varying covariates) were not explored.

**Response:**

**A. Testing proportional hazard assumption was obligatory in our initial analysis.**

**In our initial analysis, testing proportional hazard assumption (Schoenfeld residuals test) was an obligatory step performed in all experiments for both univariate and multivariate analysis.**

**Because in multivariate analyses the proportional hazards assumption can occasionally fail statistically due to subgroup composition, limited event numbers, or sample size - even when the hazard relationship appears approximately linear - we applied a slightly more lenient significance threshold for the Schoenfeld residuals test (p < 0.05) compared with univariate analyses (p < 0.01).**

**We additionally reviewed how many parameters in the multivariate analysis showed a borderline p-value (0.01–0.05) for the proportional hazards assumption test and found only single parameters (summarized below), which we consider acceptable.**

**Parameters in the multivariate analysis with borderline p-values (0.01–0.05) for the proportional hazards assumption test**

| Cohort | Number of parameters* |
|---|---|
| LUAD | OS: 12, CSS: 11, PFS: 14 |
| LUSC | OS: 37, CSS: 24, PFS: 16 |
| COAD | OS: 7, CSS: 9, PFS: 24 |
| BRCA | OS: 7, CSS: 9, PFS: 24 |
| HNSC | N/A (LUSC = exploration cohort) |

**Comment: *substantial overlaps among different survival endpoints.**

**Therefore, we consider all parameters included in the initial analysis to have successfully passed the proportional hazards assumption test.**

**We update our supplementary tables to include relevant metrics (including p-value, Shoenfeld residuals, etc.)**

**B. Spline-based Cox proportional hazards model with time-varying covariates (additional experiments).**

**Until now, all parameters (including derived variables) that did not satisfy the proportional hazards assumption had been excluded from the analysis. We have now re-evaluated these parameters using a spline-based time-varying covariate Cox model (Table 2 below).**

**Prognostic value of parameters that violated the proportional hazards assumption (spline-based time-varying covariate Cox model)**

| Cohort | Significant univariate*, Parameters n | Significant multivariate*, Parameters n | Already in the list (passed PH test as other derivative) |
|---|---|---|---|
| LUAD | 230 | 110 | 97/110 |
| LUSC | 171 | 103 | 103/149 |
| COAD | 430 | 395 | 345/395 |
| BRCA | 137 | 74 | 65/74 |

**Comment: results presented are without FDR correction.**
***For all prognostic endpoints together**

**During our review, we also noted that most parameters identified as significant in the univariate analysis were derivatives of parameters that had already satisfied the proportional hazards assumption, often in combination with another derivative (Table 2).**

**Given the minimal additional significance of these findings (considering pending FDR adjustment, multi-stage decorrelation, and independent validation), and in line with requests from reviewers and editors to reduce the complexity of the manuscript, figures, and supplementary materials, we have decided to focus on the parameters that met the proportional hazards assumption. We clearly state this in the Methods sections (“Prognostic analysis”, Page 28)**

The newly obtained results for parameters that violated the proportional hazards assumption can be made available as a structured dataset in an open-access repository (e.g., Zenodo) upon request or as part of supplementary data.

C. Reliance on p-values.

We not only relied on p-values, but also always considered the prognostic effect size of significant parameters (hazard ratios) and provided stratifications based on the most influential parameters. As expected, the hazard ratios were highly correlated with the corresponding p-values.

D. Interaction.

With regard to parameter interactions, we acknowledge that this is an important aspect of any prognostic analysis and expect a broad range of interaction strengths (from minimal to substantial). However, we believe this would not materially affect our selection pipeline, which— as outlined above— incorporates multiple decorrelation steps at several levels, including technical decorrelation and prognostic risk–level decorrelation with a stringent Jaccard index cutoff (< 0.85; i.e. between two parameters, of 100 patients, 15 must have different prognostic risk classification) to retain only parameters providing clearly divergent classifications. Furthermore, our multiparametric systems consistently demonstrate complementary effects among different parameters, indicating that potential interactions do not have detrimental effects on the results.

Given the strong requests to reduce the complexity of the manuscript, we have decided to omit this analysis. As all databases are being made available as accompanying open-source resources, users can perform targeted exploration of parameter interactions if desired.

E. Subgroup analysis.

We now provide subgroup analyses for all relevant clinical subgroups and cross-cohort comparisons, separately for each tumor type (Table 3).

Clinical subgroups for extended prognostic analysis

| Cohort | Number of parameters |
|---|---|
| LUAD | pT1, pT1-2, pN0 |
| LUSC | pT1, pT1-2, pN0 |
| COAD* | pT1, pT2-3, pT3, pN0, pN0-1 |
| BRCA | pT1, pT1-2, pN0, pN0-1, ER, histological subtype |
| HNSC | Not possible (low number of cases/events) |

Comment: *MSI – not enough cases (only relatively small HAL cohort has MSI status/survival information)

We include all sub-cohorts for which prognostic data are available, including data for specific prognostic endpoints where the parameters were validated, provided that the number of patients/events is adequate. The same setup as in the initial experiments is applied — multivariate analysis is performed only when at least 10 events are

**observed in each subgroup to ensure sufficient statistical power. We also report the number of parameters with p < 0.1 in the multivariate analysis to indicate statistical trends that may be affected by reduced power due to a low number of cases or events.**

**Subgroup and cross-cohort analyses are always conducted for the independent test cohort of each tumor type, using only parameters and thresholds established in the exploratory cohorts, and validated on the full independent cohort.**

**The number of significant parameters is then compared with those identified in the full test cohort (main results).**

**Legend: Full test cohort | Cross-cohort comparison | Subgroups**
**N_non_na – analysis was possible**
**N_missing – analysis not possible (due to power limitations)**
**N p<0.05 – number of parameters with multivariate Cox p<0.05**
**N p<0.1 – number of parameters with multivariate Cox p<0.1 (trend)**

**BRCA: summary of subgroup validation**

| Cohort_Endpoint | N p<0.05 | N p<0.1 | N_non_na | N_missing |
|---|---|---|---|---|
| FULL_OS_P | 34 | - | 34 | - |
| ERneg_OS_P | 5 | 11 | 33 | 1 |
| ERpos_OS_P | 21 | 26 | 34 | 0 |
| NOS_OS_P | 19 | 28 | 34 | 0 |
| Lobular_OS_P | 9 | 13 | 22 | 12 |
| pN0_OS_P | 14 | 17 | 29 | 5 |
| pN0-1_OS_P | 18 | 30 | 34 | 0 |
| pT1_OS_P | 6 | 9 | 33 | 1 |
| pT1-2_OS_P | 22 | 29 | 34 | 0 |

**Comment: Only one test cohort (no cross-cohort comparison)**

**LUAD: summary of subgroup validation**

| Cohort_Endpoint | N p<0.05 | N p<0.1 | N_non_na | N_missing |
|---|---|---|---|---|
| FULL_CSS_P | 74 | 74 | 74 | |
| NLST_CSS_P | 26 | 39 | 74 | 0 |
| PLCO_CSS_P | 41 | 52 | 73 | 1 |
| pN0_CSS_P | 64 | 69 | 74 | 0 |
| pN0-1_CSS_P | 69 | 73 | 74 | 0 |
| pT1_CSS_P | 46 | 53 | 74 | 0 |
| pT1-2_CSS_P | 49 | 64 | 74 | 0 |

**LUSC: summary of subgroup validation**

| Cohort_Endpoint | N p<0.05 | N p<0.1 | N_non_na | N_missing |
|---|---|---|---|---|
| FULL_OS_P | 33 | | 33 | |
| NLST_OS_P* | 4 | 5 | 30 | 3 |
| NLST_PLCO_OS_P | 18 | 24 | 33 | 0 |

| | | | | |
|---|---|---|---|---|
| PLCO_OS_P | 12 | 13 | 19 | 14 |
| UKE_UKK_OS_P | 12 | 16 | 24 | 9 |
| UKK_OS_P* | 7 | 12 | 24 | 9 |
| pN0_OS_P | 26 | 30 | 33 | 0 |
| pN0-1_OS_P | 26 | 32 | 33 | 0 |
| pT1_OS_P | 14 | 15 | 33 | 0 |
| pT1-2_OS_P | 21 | 27 | 33 | 0 |

**Comment: *Low event number**

**COAD: summary of subgroup validation**

| Cohort_Endpoint | N p<0.05 | N p<0.1 | N_non_na | N_missing |
|---|---|---|---|---|
| FULL_OS_P | 18 | | 18 | |
| FULL_CSS_P | 66 | | 66 | |
| PLCO_OS_P | 17 | 18 | 18 | 0 |
| pN0_OS_P | 6 | 10 | 17 | 1 |
| pN0-1_OS_P | 13 | 15 | 18 | 0 |
| pT1_OS_P* | 1 | 1 | 5 | 13 |
| pT2-3_OS_P | 13 | 17 | 18 | 0 |
| pT3_OS_P | 12 | 14 | 18 | 0 |
| PLCO_CSS_P | 58 | 62 | 66 | 0 |
| pN0_CSS_P | 27 | 32 | 48 | 18 |
| pN0-1_CSS_P | 49 | 57 | 64 | 4 |
| pT2-3_CSS_P | 49 | 56 | 66 | 0 |
| pT3_CSS_P | 38 | 46 | 65 | 1 |

**Comment: *Low event number**

**With this approach, we demonstrate that a substantial number of parameters can be validated in clinically relevant subcohorts as well as in cross-cohort settings. Comprehensive results, including parameter names and full statistical outputs (hazard ratios, C-indices, confidence intervals, etc.), will be provided as archive via Zenodo repositorium (given strong request to reduce the number of supplementary materials). We attach the results as zip archive for peer-review.**

**Although the number of parameters showing statistical significance is lower than in the full cohort, we wish to specifically highlight the following considerations that should be taken into account:**

**1) Stringent parameter filtering: All parameters underwent rigorous multi-step filtering prior to validation, which may have excluded some potentially important variables. Specifically, we applied a sequential pipeline (exploration cohort: risk stratification-based decorrelation → univariate → FDR correction → multivariate Cox → test cohort: multivariate Cox analysis). Many decorrelated parameters were eliminated already during the univariate stage in the exploration cohort.**
**2) Reduced statistical power in subcohort analyses: Naturally, subgroup analyses are affected by smaller sample sizes and event numbers, which reduce statistical power.**
**3) Biological insight beyond stringent validation: Even parameters that do not pass the refined independent validation steps (often due to failure of reaching significance using the same threshold as in exploration) may still carry valuable biological**

**information. In addition to their prognostic potential, our analyses reveal emerging biological patterns that can guide future spatial biology experiments. Such insights can be extracted directly from the univariate analyses.**
**4) SPARK is a foundational step toward broader application: This study establishes a strong conceptual framework that can be extended and refined into a state-of-the-art approach for scientific exploration. The presented principles should now be further developed, implemented, and prospectively validated across specific tumor types and niche research areas.**
**5) Cohort-specific biases: Potential biases inherent to the cohorts used (e.g., TCGA: usually one slide/patient, some preselection for tumor parts with maximal tumor cell content for sequencing studies) should be acknowledged when interpreting the results – this specifically affects the selected thresholds that might require additional, prospective validation.**

- Robustness under realistic data noise and missingness was not tested, limiting confidence in real-world applicability.

**Response:**

**Many thanks for this important comment, which has two key implications for clinical practice. First, the issue of data missingness is particularly relevant to digital pathology compared with other diagnostic specialties. While analyses of resection specimens may seem to offer a robust starting point, several types of artifacts can still contribute to missing data. The most notable are out-of-focus regions resulting from the digitization process, which must be excluded from analysis. In our study, we address this by implementing a rigorous quality control procedure using the GrandQC tool (Weng Z. et al., Nature Communications, 2024). Additionally, variations in grossing and embedding techniques — which may result in differing amounts and parts of tumor tissue available for evaluation — can further introduce missingness into the dataset.**

**Second, although the biopsy setting lies outside the primary focus of our study, it nonetheless represents an important clinical scenario that inherently involves realistic levels of noise and data missingness. We address both aspects through a comprehensive experiment in which we test our parameters across all tumor types by analyzing randomly selected regions of the tumor, ranging from 1×1 mm² (representative of a typical small biopsy sample) up to 64 mm². This includes incremental region sizes of 1, 2, 4, 8, 16, 32, and 64 mm².**

**The endpoint of this analysis is the concordance of prognostic classification for each significant parameter (i.e., the list of independently validated parameters identified in the test cohorts) relative to the ground truth classification obtained from the analysis of the entire tumor. For each tumor type, we use its most well-characterized prognostic endpoint: LUAD - CSS, LUSC - OS, COAD - CSS, and BRCA - OS.**

**In the new Extended Data Fig. 6 (provided below; Referred from Results sections, Page 15), we present how the classification accuracy of relevant parameters changes with increasing tumor area analyzed (panels A–D). The results demonstrate reasonable levels of accuracy across tumor types. We further use this approach as a selection tool to identify parameters with the highest and lowest performance under restricted tumor area analysis. Parameters illustrated in panels E, G, I, and K exhibit greater stability and homogeneity, achieving high prognostic classification accuracy even when only small, biopsy-sized regions of the tumor are analyzed. Based on**

these findings, we generated a list of parameters that may be preferentially implemented in biopsy-based diagnostic or prognostic settings.

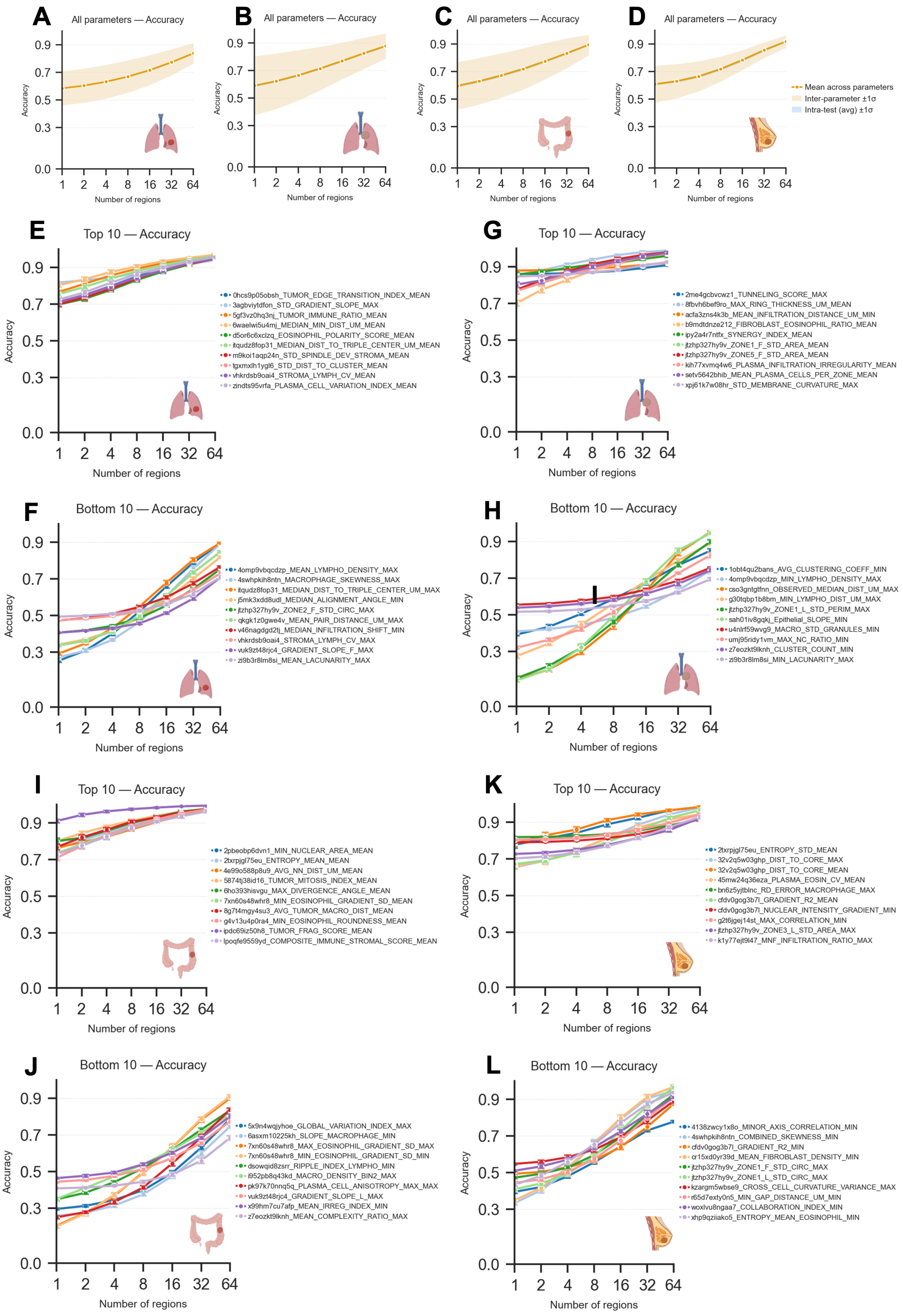

**Extended Data Fig. 6 (new)**

Impact and Interest
The paper is likely to attract interest across pathology, oncology, and computational biology communities. By bridging human-expert input with AI-driven hypothesis generation, SPARK reduces barriers to testing novel ideas. Until histopathology-specific vision–language models are developed, such agentic workflows will play an important role in scaling exploratory research. The temporal evolution analysis in particular may stimulate new ways of thinking about tumor progression.
Outstanding Features
- Conceptual novelty: using natural language as a universal interface.
- Modular, transparent, and reproducible workflow with commitment to open-source release.
- Demonstrated versatility across prognostic, predictive, spatial, and temporal applications.
- Enables human–AI collaboration by translating free-text ideas into measurable features.
- Competitive predictive performance for biomarkers such as PD-L1 and MSI.

**Response:**

**Thank you for your positive assessment of the merit of our work and for summarizing several important aspects that we have addressed in the Discussion section of the manuscript.**

Statistical and Methodological Considerations
Statistical analyses are only partially adequate. The reliance on dichotomization and p-values without effect sizes, C-index, or cross-cohort stability weakens claims of clinical relevance.

**Response:**

**This comment has already been addressed above (specifically regarding the reliance on p-values, assumptions, cross-cohort, and subgroup analyses).**

**Additionally, we would like to note that a significant portion of the exploration and testing in independent cohorts involves using the parameters as quantitative variables (Figure 4e-f; Extended Data Fig. 3a – for overlap between same parameters as categorical and continuos variables; Suppl. Tables 31,33,35,37,39). However, dichotomization or categorization into multiple groups (Figure 4l-t) is often more appealing from a clinical perspective (limited number of clearly defined groups).**

**Moreover, to further address this reviewer´s comment, we have extended our analysis to include a C-index–based comparison against clinical baselines in each tumor type. In the updated Suppl. Tables to prognostic analysis (Suppl. Tables 30,32,34,36,38), we now present the C-index for multivariate models including each parameter, alongside the C-index of the corresponding clinical baseline, the delta between the two indices, and the p-value from the Likelihood Ratio Test (LRT) comparing them. In the vast majority of models, we observe concordance between the significant p-values in the multivariate Cox analysis and those from the comparison of the two C-indices (with only a few exceptions showing borderline significance).**

Proportional hazards assumptions were not tested;

**Response:**

**As noted above, these tests were applied across all analyses performed for the initial submission (with further details provided above).**

Schoenfeld residuals and time-varying covariates should be applied where appropriate. Non-linear effects could be modeled using restricted cubic splines.

**Response:**

**Schoenfeld residuals were used for proportional hazard assumption test in all initial results. Spline-based Cox proportional hazards model with time-varying covariates was applied to parameters that violated proportional hazard assumption test (details of analysis are provided above).**

Error bars and confidence intervals should be clearly defined in figure legends, and discrimination metrics should supplement p-values for feature selection.

**Response:**
**We added this information, where applicable.**

Suggestions for Improvement
- Develop or fine-tune domain-specific LLMs to improve biological reasoning and reduce reliance on costly commercial models.

**Response:**

**Thank you for this important comment, which has direct implications for the broader adoption of SPARK.**

**At this stage, we are conducting large-scale experiments involving multiple state-of-the-art large language models (LLMs), both general-purpose and medical domain–specific, to evaluate their suitability for the idea-generation step. Importantly, we selected models from different classes, including some that can be executed on low-resource, consumer-grade hardware.**

**A summary of the LLMs used is provided below. Although we do not include very large open-source models (e.g., DeepSeek V3 with 671B parameters or Llama 4 Maverick with 400B parameters), which require HPC clusters for deployment, we acknowledge that some users may have access to such infrastructure. As shown below, scaling laws still apply, and even greater performance can be expected. Our focus, however, is on more realistic deployment scenarios.**

**Open-source, locally deployed models used to evaluate reasoning capabilities in the context of idea generation**

| Model | Type | Number of parameters, | Hardware used | Comment |
|---|---|---|---|---|
| **Generalist LLMs** | | | | |
| DeepSeekR1 | Generalist | 70 b | Server<br>4x A100 80Gb | Llama3-distilled version |
| gpt-oss-120b | Generalist | 120 b | Server<br>4x A100 80Gb | - |

| | | | | |
|---|---|---|---|---|
| **gpt-oss-20b** | **Generalist** | **20 b** | **Server<br>2x L40S 48Gb** | **Can be deployed on MacBook Pro or similar** |
| **Qwen3:32b** | **Generalist** | **32 b** | **MacBook Pro<br>M1 Ultra 64Gb RAM** | **-** |
| **Medical domain LLMs** | | | | |
| **Meditron-70B** | **Medical** | **70 b** | **Server<br>4x A100 80Gb** | **-** |
| **MedGemma** | **Medical** | **27 b** | **MacBook Pro<br>M1 Ultra 64Gb RAM** | **-** |
| **BioMistral** | **Medical** | **7 b** | **MacBook Pro<br>M1 Ultra 64Gb RAM** | **-** |

**For each open-source model in the idea generation task, we used exactly the same setup as in the initial experiments. Although we could have freely used non-commercial models for the idea review and idea duplicate detection subtasks (which are less demanding than idea generation), we chose to use the commercial o3-mini model (OpenAI), as in our initial experiments, to ensure full comparability of the experimental results.**

**The results are summarized in Extended Data Figure 1j (best-performing open-source model), with the complete results provided in Supplementary Figures 1 and 2 (referred to from the Result section to idea generation), also presented below:**

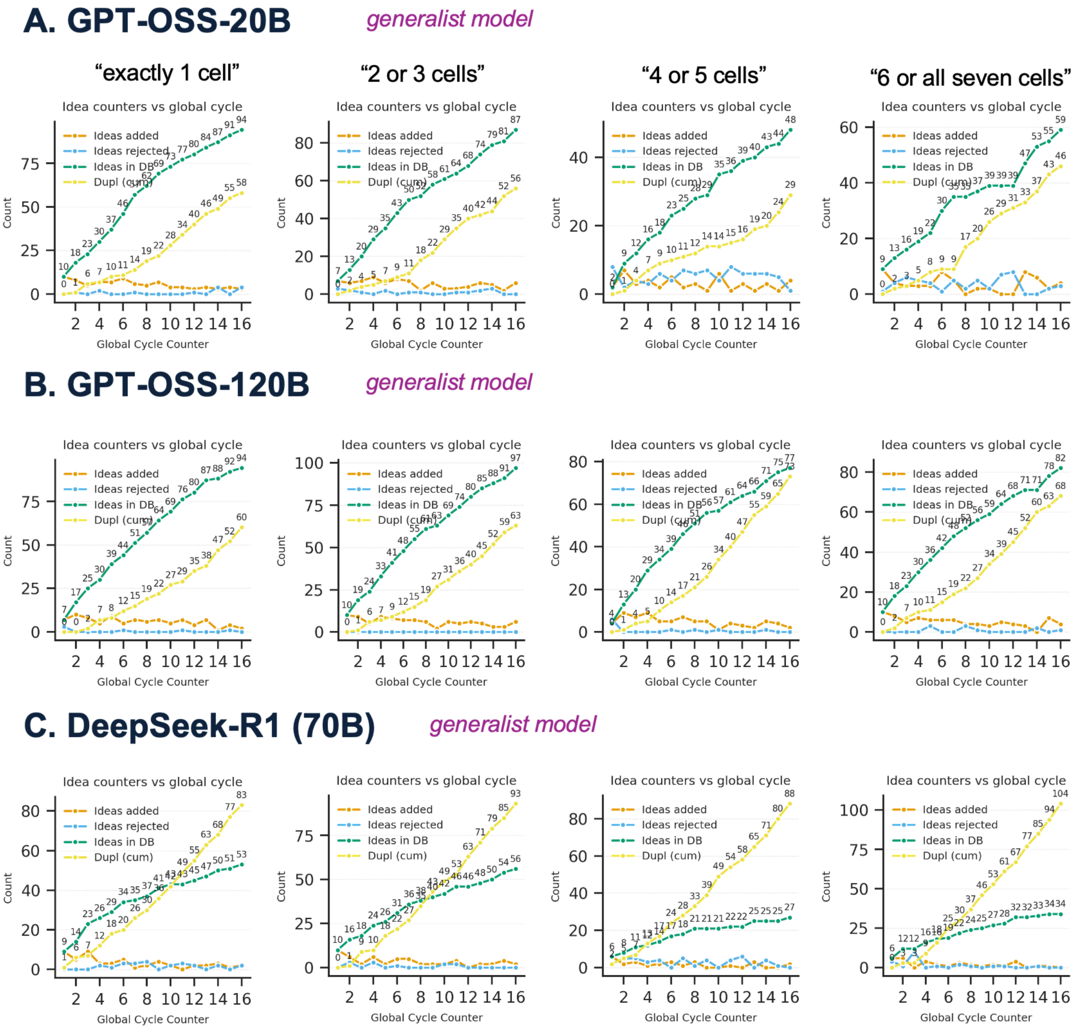
A. GPT-OSS-20B
generalist model
"exactly 1 cell"
"2 or 3 cells"
"4 or 5 cells"
"6 or all seven cells"
B. GPT-OSS-120B
generalist model
C. DeepSeek-R1 (70B)
generalist model
Idea counters vs global cycle
Ideas added
Ideas rejected
Ideas in DB
Dupl (cum)
Count
Global Cycle Counter

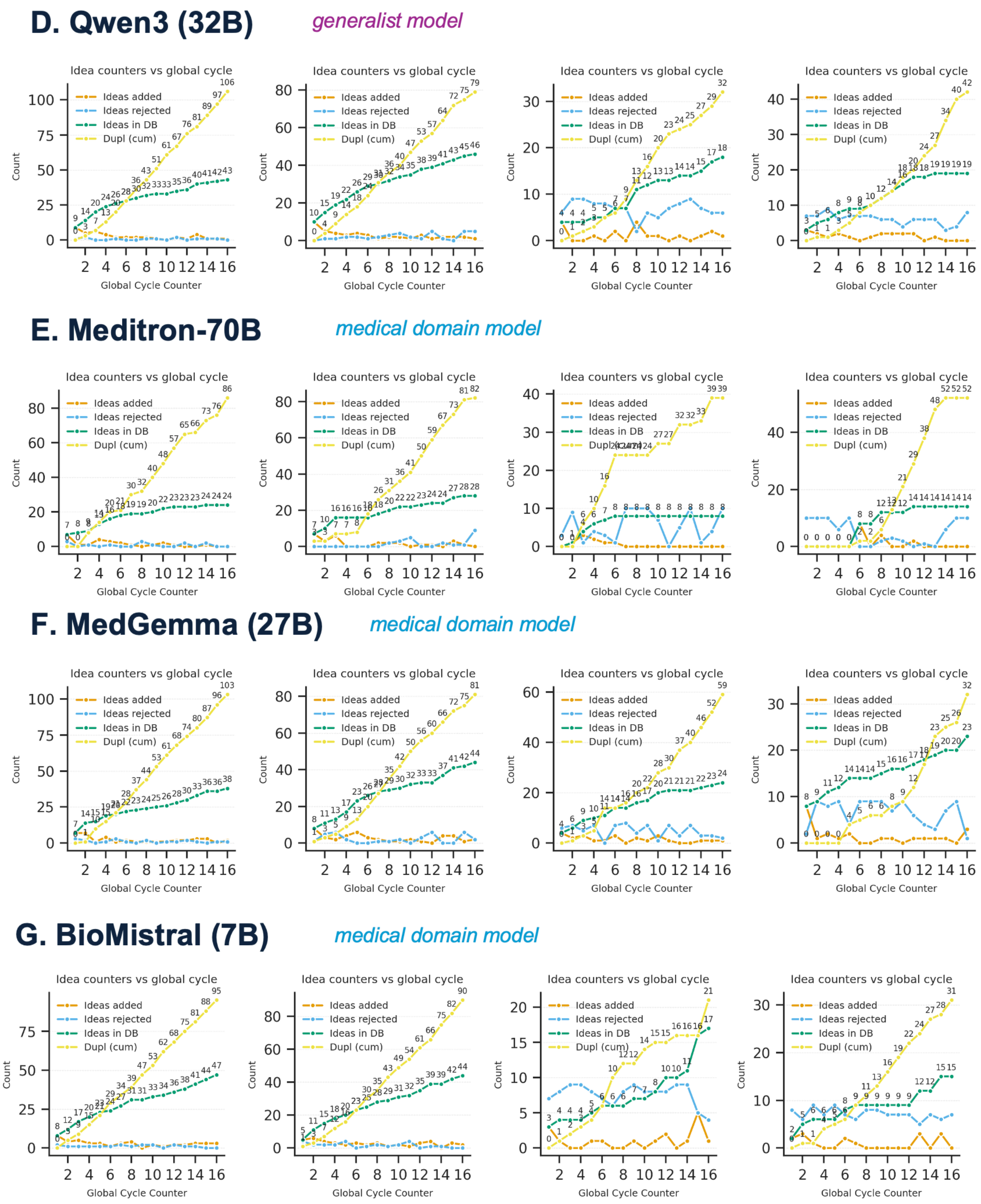


**Suppl. Figure 1.**

**First, we observe notably better results—with more ideas generated, fewer duplicates, and higher compliance with guidelines (i.e., fewer idea rejections due to violations)—for larger models, particularly gpt-oss-120b. This trend also holds for reasoning-oriented models (gpt-oss-120b, gpt-oss-20b, DeepSeekR1 70b with Llama3 distillation) and generalist models. Larger models clearly demonstrate a greater capacity to process and manipulate information, leading to improved performance in dense informational contexts (e.g., when a large number of ideas have already been generated).**

**Second, we did not observe any advantage for models specialized in the medical domain. On the contrary, we had to tighten the task descriptions due to multiple**

additional failures resulting from guideline violations. The most frequent issue was as follows: while medical models were generally able to identify one or a few (2–3) relevant cell types, they consistently struggled to generate more complex ideas involving 4–7 cell types, repeatedly violating the cell number constraint (see the number of rejected ideas in the plots above). These models also produced a substantial number of duplicate ideas with in general low overall yields. Overall, we found no evidence of a competitive advantage in the complexity or diversity of the generated ideas.

We believe that three main factors contribute to this lower performance: (1) most medical models are not reasoning-oriented for now, (2) they are typically smaller in scale (as larger versions are difficult to fine-tune), and (3) their training data are primarily clinically oriented, whereas our tasks focus on tumor microenvironment, tumor biology, and single-cell contexts.

In general, the inferiority of the medical models to generalist models even on medical tasks is a known fact that we confirm in our particular use case. For example, MedGemma (https://arxiv.org/pdf/2507.05201): significantly inferior to o3 in 10/11 medical benchmarks  and in 7/11 medical benchmarks to GPT-4o; BioMistral: (https://arxiv.org/abs/2402.10373) is significantly inferior on 10/10 cinical benchmarks to GPT3.5.

From the perspective of semantic analysis, we observe patterns similar to those found in our initial experiments (Supplementary Figure 2 and below). We conclude that larger and more generalist models are better able to bridge gaps between existing ideas, continuing to identify novel and interesting approaches even in saturated idea spaces.

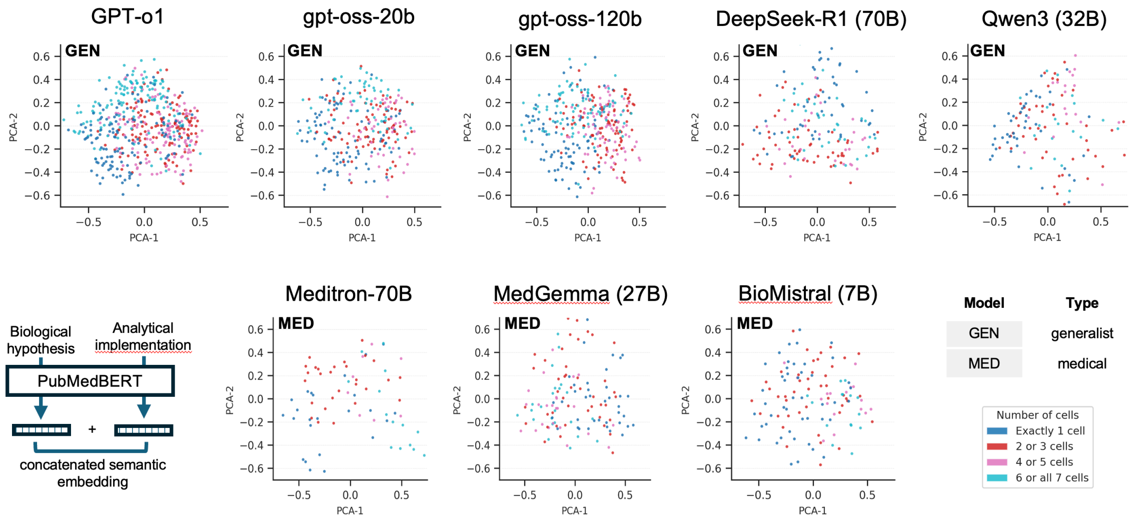


**Suppl. Figure 2**

Overall, we find that open-source models can serve as a viable alternative to commercial models.

We also evaluated another critical component of the SPARK pipeline - parameter coding - to assess whether comparable quality can be achieved using open-source models. For this purpose, we employed two larger reasoning models: DeepSeekR1 model (Llama3-distilled, 70B) and the gpt-oss-120b model.

We obtained encouraging results (summarized in Supplementary Figure 6 and below), confirming that open-source models can fully meet the requirements of the coding step. In particular, gpt-oss-120b demonstrated coding performance indistinguishable from that of Claude 3.5, which was used in the initial experiments. DeepSeekR1 showed slightly lower performance; however, this can likely be improved by refining the coding task definitions, as most of the observed errors were minor.

### E. Use Case 1: Coding of parameters as functions

| Workflow stage | gpt-oss-120b, n | DeepSeek R1 (70B), n |
|---|---|---|
| Ideas generated (original) | 500 | 500 |
| Python code generated | 500 (100%) | 500 (100%) |
| Python code executed without errors | 495 (99%) | 236* (47.2%) |
| Passed time test (60 s / small test case) | 495 (99%) | 224 (44.8%) |
| Final filtering | 463 (92.6%) | 168 (33.6%) |

*Mostly simple errors: syntaxis, missing quotes for comments, unimported packages (can be addressed through tightening of task description)

**Suppl. Figure 6**

In conclusion, our results provide evidence that SPARK can be implemented with high performance using fully open-source, locally running models. However, the best results are achieved with the largest, most capable, generalist models. At this stage, medical domain models do not appear to be the models of choice, primarily due to their smaller size, limited and clinically oriented pre-training, and largely absent reasoning capabilities.

We will provide detailed end-user guidelines in our GitHub repository accompanying these benchmarks for idea generation and coding.

- Introduce a novelty metric (e.g., semantic distance in embedding space) to prioritize conceptually distinct ideas.

**Response:**

As mentioned above (in response to another reviewer's comment), we developed a new module that, as recommended, maps ideas based on their semantic composition. This module enables prioritization of ideas that occupy outlier positions in the semantic space - i.e., those with distinct formulations - by measuring their relative distance.

Although this approach improves interpretability, it also has implications for computational sustainability, since generating embeddings requires running LLMs once per model. Nevertheless, we do not necessarily believe that embedding-based

**models offer superior value when it comes to comparing two or multiple ideas. Because most ideas are formulated concisely, modern generalist LLMs are capable of performing direct, detailed comparisons without relying on vector embeddings (typically of dimension <1024). However, this direct comparison approach requires a systematic setup and performs best when applied to a limited number of ideas within a single prompt.**

- Allow flexible data structures to accommodate additional modalities and hypotheses beyond the current rigid schema.

**Response:**

**Although the task formulation may appear rigid, its structure is highly flexible and can be readily adapted to other use cases without requiring significant modifications to the underlying code. We acknowledge that certain modalities, such as radiology, represent particularly promising extensions where SPARK could deliver comparable clinical or diagnostic value. However, such an adaptation would require substantial domain-specific adjustments and falls outside the scope of the present work, especially given the absence of radiology experts among the authors.**

**Nevertheless, we have introduced - and will make available to end users - an additional SPARK extension designed to enhance performance and facilitate scalability across diverse use cases: the integration of literature-derived evidence to enrich the idea generation context.**

**This extension parses publication PDFs - for example, a review article on the role of the tumor microenvironment in malignant tumors - into a small, dense narrative relevant to the idea generation task. The narrative size is defined by the operator, taking into account the context length supported by the selected idea generation LLM. This extracted information can then be optionally incorporated as an additional input during the idea generation process.**

**We did not evaluate this extension in detail in the present study; however, we consider it a useful enhancement for SPARK end users. The extension is included in the revised codebase and will be released in our open-source GitHub repository.**

- Explore interaction effects and subgroup analyses (e.g., stage-specific or treatment-specific subgroups).

**Response:**

**The response to this comment was provided above.**

- Assess robustness under noisy or incomplete datasets to approximate real-world conditions.

**Response:**

**The response to this comment was provided above.**

- Provide clearer narrative descriptions of algorithms in the manuscript and relegate raw variable names to supplementary code repositories.

**Response:**

**We appreciate the reviewer's suggestion to provide more narrative descriptions of the algorithms. However, due to the manifold of different analysis and the large number of parameters involved (over hundred significant variables, each with established abbreviated identifiers), providing expanded narrative forms for all variables within the manuscript would exceed space limitations and significantly hinder readability.**

**Additionally, these abbreviated variable names are consistently used throughout the implementation and supplementary materials, and altering them to more descriptive forms would risk inconsistencies and reduce reproducibility.**

**We believe that providing an accompanying HTML reference document (included as supplementary material), which contains a comprehensive and searchable list of all parameter names along with their detailed explanations, offers an effective means of navigating and understanding the numerous variables used in the study. This solution ensures both transparency and accessibility while keeping the manuscript concise and within space limitations.**

**Reviewer #3 (Remarks to the Author):**

PREFACE
I am not a pathologist, oncologist, or computer scientist; I likely represent many Nature Medicine readers if this is published. I am a clinician with an interest in AI/LLMs. My comments focus on clarity, clinical relevance, and safeguards (leakage, multiplicity, generalisability). Some of my comments may reflect technical naivety, however this would probably be expected for other readers as well. I defer technical methodology to technical reviewers. I apologize for the length, but it is also an imposing article.

**Response:**

**We sincerely thank the reviewer for their thoughtful and constructive comments. As most of the manuscript´s authors are clinically practicing pathologists, we appreciate this reviewer´s clinical perspective and careful attention to clarity, clinical relevance, and safeguards. Their insights, particularly from the standpoint of a non-technical clinician reader, are invaluable in improving the accessibility and impact of the manuscript.**

KEY RESULTS
The authors present SPARK (System of Pathology Agents for Research and Knowledge), an agentic framework that: (i) uses language to generate biomarker ideas; (ii) auto-codes them into computable features ("parameters"); and (iii) evaluates them for prognosis/prediction across multiple cohorts/tumour types. They state SPARK requires no training (for parameter generation), scales to hundreds of ideas, and achieves strong—sometimes state-of-the-art—performance. Parts of the system/code will be open-sourced.

VALIDITY
Ambitious scope and impressive scale. Several guardrails should be made explicit for non-specialist readers to assess the claims.

DATA & METHODOLOGY
Leakage guardrails (major point).
Please confirm that threshold choices for dichotomising features, feature pruning/"decorrelation," and any other selection steps were made only in the exploration/training data (or within cross-validation) and then kept fixed for all independent

tests. Please state this explicitly for the human-initiated module as well, where it seems ad-hoc changes were made.

**Response:**

**A) Main Experiments.**

**Yes, the threshold and all other model choices were determined exclusively using the exploratory cohorts for each tumor type, ensuring there was no data leakage.**

**In brief, the analysis steps for the exploratory cohort were as follows:**
**1. Technical decorrelation of parameters at the regional measurement level.**
**2. Univariate analysis with optimal threshold selection.**
**3. False Discovery Rate (FDR) correction applied to the univariate results.**
**4. Decorrelation at the prognostic stratification level, assessed via the Jaccard score on dichotomized groups for each parameter.**
**5. Multivariate analysis performed using the same thresholds as established in the univariate stage.**

**This process yielded a set of significant parameters, which were subsequently validated in the multivariate analysis on the independent test cohort, using the same thresholds established in the exploratory phase. The exploratory and test cohorts were entirely independent.**

**B) Human features.**

**Our main objective here is not to conduct an advanced validation, but rather to demonstrate the feasibility of SPARK in effectively translating human ideas into action and substantially amplifying human exploratory capabilities. We prefer to keep the presentation of analyses as they are, following the strong recommendation to simplify the complexity of the result presentation.**

**Nevertheless, we performed the requested validation analysis and present the results as Supplementary Data. These include tests of human-suggested parameters (significant parameters from multivariate analysis in exploration cohort) on an independent patient cohort (merged NLST and PLCO LUAD cohort; same methodology as in the main experiments). The results confirm that many features retain their prognostic significance in this independent validation at exactly same thresholds that were established in the exploration (presented below and in Extended Data Figure 7).**

P

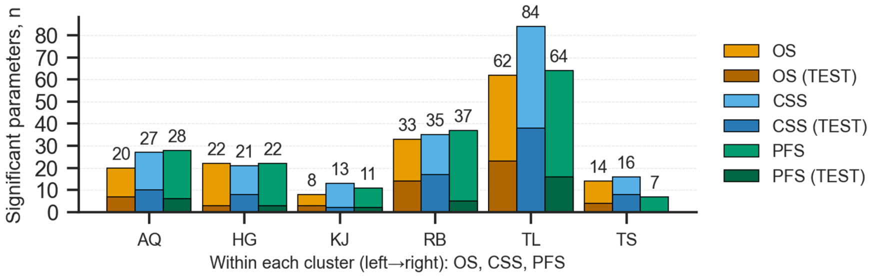


| Pathologist | OS | CSS | PFS |
|---|---|---|---|
| **P1 exploration** | 20 | 27 | 28 |
| **P1 test** | 7 | 10 | 6 |
| **P2 exploration** | 22 | 21 | 22 |
| **P2 test** | 3 | 8 | 3 |
| **P3 exploration** | 8 | 13 | 11 |
| **P3 test** | 3 | 2 | 2 |

| Pathologist | OS | CSS | PFS |
|---|---|---|---|
| **P4 exploration** | 33 | 35 | 37 |
| **P4 test** | 14 | 17 | 5 |
| **P5 exploration** | 62 | 84 | 64 |
| **P5 test** | 23 | 38 | 16 |
| **P6 exploration** | 14 | 16 | 7 |
| **P6 test** | 4 | 8 | 0 |

Extended Data Figure 7 (new)

**Importantly, we tested parameters that showed significance in the exploratory multivariate analysis without FDR correction. The rationale was that the participating human experts did not wish to dismiss any of the ideas they had contributed simply because they failed to meet strict FDR criteria—an approach that likely mirrors real-world exploratory reasoning.**

**We will deposit the full results of these analyses in a Zenodo repository upon publication, to avoid overloading the Supplementary Tables.**

C) Analysis of multiplexed data (METABRIC cohort).

**Similarly, our primary objective here is to demonstrate the feasibility of SPARK for this advanced use case. We do not aim to overcomplicate the analysis and therefore prefer to keep the presentation of results unchanged, based on the following consideration: in this specific context, we believe that the features derived from the full cohort - leveraging the complete statistical power of the exploratory phase - are more relevant for potential downstream applications, detailed biological interpretation, and implementation by end users of SPARK, compared to those obtained through cross-validation or cohort splitting.**

**Nevertheless, we performed the requested validation analysis and present the results as Extended Data Figure 8e-h (also presented below). Briefly, the cohort was randomly divided into exploration and test subsets (each comprising approximately 50% of the original cohort). The split was stratified to ensure comparable distributions of pT, pN, estrogen receptor status, and histological grade, thereby maintaining representativeness (Panel e).**

**E**

METABRIC cohort

Exploration (n=313)

| Parameter | Cases n |
|---|---|
| pT1/2/3/4 | 11/116/170/16 |
| pN0/1/2 | 145/64/104 |
| G1 / G2 / G3 | 31/126/152 |
| ER pos./neg | 245/63 |
| Her2 pos/neg | 242/30 |
| CSS event | 88 |

Test (n=314)

| Parameter | Cases n |
|---|---|
| pT1/2/3/4 | 10/117/172/15 |
| pN0/1/2 | 145/65/104 |
| G1 / G2 / G3 | 25/102/182 |
| ER pos./neg | 243/66 |
| Her2 pos/neg | 242/30 |
| CSS event | 111 |

**F**

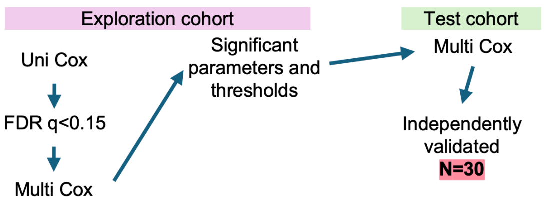


**G**

**H**

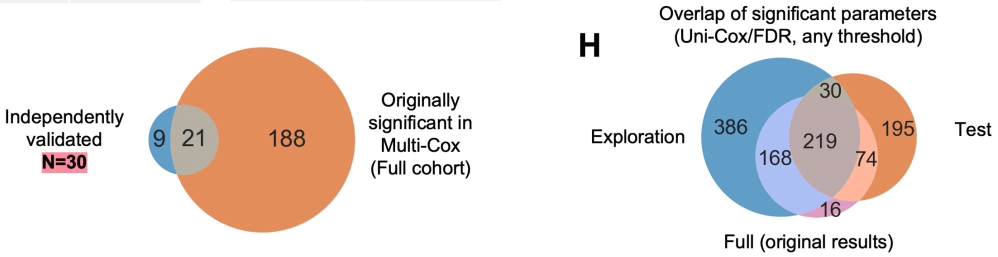


**Extended Data Figure 8 (new)**

**We performed two types of analyses:**

**1. Fully independent parameter selection (similar to the main experiments using H&E-slides).**

**Parameters were independently selected on the exploration cohort (<u>Panel f</u>), following the same procedure as in the main experiments. The only difference was the use of a slightly relaxed FDR threshold (q = 0.15) in the univariate analysis to compensate for the reduced statistical power compared to the initial full cohort. The parameters and thresholds identified in the exploration cohort were then applied to the test cohort, resulting in 30 parameters validated in multivariate analysis - 21 of which overlapped with those identified in the initial full-cohort multivariate analysis (Panel g).**

**2. Assessment of univariate significance and threshold robustness.**

**Given that the data are derived from small tissue microarray spots containing tumor tissue and thus may be subject to substantial sampling bias, optimal thresholds can vary, potentially leading to loss of prognostic significance even for biologically relevant features. To address this, we additionally generated lists of parameters significant in univariate analysis after FDR correction (serving as correlates of biological relevance). Thresholds may differ here between the exploration and test cohorts. Nonetheless, we observed substantial overlap between the exploration, test, and initial full-cohort results, further supporting the robustness and validity of the identified parameters (<u>Panel h</u>).**

**The significant parameters from the independent validation are presented in detail in Suppl. Fig. 10, including comparison vs baseline (C-indeces, delta, and likelihood-ratio test).**

| Parameter | CSS_HR | CSS_P | CSS_CI_LOW | CSS_CI_HIGH | CSS_CINDEX_BASE | CSS_CINDEX_FULL | CSS_CINDEX_DELTA | CSS_LRT_P |
|---|---|---|---|---|---|---|---|---|
| i3ew4evg5a4d_M_proportion | 2.64 | 0.0004 | 1.53 | 4.53 | 0.50 | 0.59 | 0.09 | 0.0011 |
| 9i7j2li19588_TU_norm_boundary_stroma | 1.98 | 0.0023 | 1.28 | 3.08 | 0.50 | 0.59 | 0.09 | 0.0013 |
| td29c04foc15_TU_to_Tc_ratio | 1.88 | 0.0027 | 1.25 | 2.85 | 0.50 | 0.59 | 0.09 | 0.0022 |
| du70lswfg3ei_M_percent | 1.79 | 0.0036 | 1.21 | 2.65 | 0.50 | 0.57 | 0.07 | 0.0030 |
| yldxliwbr70c_weighted_dist_G | 1.74 | 0.0039 | 1.19 | 2.53 | 0.50 | 0.57 | 0.07 | 0.0043 |
| rxnm2a3j57a0_M_MEDIAN_PACKING_FACTOR | 1.90 | 0.0047 | 1.22 | 2.96 | 0.50 | 0.57 | 0.07 | 0.0030 |
| fyyse1b272ey_FIBROBLAST_TO_MACROPHAGE_RATIO | 0.60 | 0.0075 | 0.41 | 0.87 | 0.50 | 0.57 | 0.07 | 0.0081 |
| pvush9sb1jbe_STD_NUCLEAR_SOLIDITY_PDPN+ Myofibroblast_Epithelial | 2.50 | 0.0080 | 1.27 | 4.92 | 0.50 | 0.61 | 0.11 | 0.0064 |
| vkzlzyx082c4_stroma_fibroblast_to_epithelial_cell_ratio | 0.57 | 0.0083 | 0.38 | 0.87 | 0.50 | 0.58 | 0.08 | 0.0064 |
| jl5tb6agvujt_E_aggregate_deformation_score | 0.52 | 0.0108 | 0.32 | 0.86 | 0.50 | 0.56 | 0.06 | 0.0064 |
| crecbc67jue4_corr_M_MF | 1.65 | 0.0113 | 1.12 | 2.42 | 0.50 | 0.56 | 0.06 | 0.0127 |
| s3gkpz5i725j_tumor_cell_proportion | 1.92 | 0.0113 | 1.16 | 3.19 | 0.50 | 0.56 | 0.06 | 0.0066 |
| 3lpt42dyr5yq_global_immuno_fibroblastic_infiltration_index | 1.69 | 0.0123 | 1.12 | 2.54 | 0.50 | 0.57 | 0.07 | 0.0099 |
| o37ukl0ly6vh_G_TH_RATIO | 1.82 | 0.0149 | 1.12 | 2.93 | 0.50 | 0.53 | 0.03 | 0.0212 |
| aav60os6mfai_TRIANGULATION_IRREGULARITY_INDEX | 0.45 | 0.0150 | 0.23 | 0.85 | 0.50 | 0.55 | 0.05 | 0.0067 |
| 5c2cnux8cz2e_composite_K_50 | 1.64 | 0.0154 | 1.10 | 2.44 | 0.50 | 0.56 | 0.06 | 0.0186 |
| hpbm5qpwotvf_TRIPLET_E-E-Ff_FREQ | 0.46 | 0.0165 | 0.25 | 0.87 | 0.50 | 0.59 | 0.09 | 0.0235 |
| 2uydkrgg0ths_G_proportion | 1.60 | 0.0174 | 1.09 | 2.35 | 0.50 | 0.56 | 0.06 | 0.0201 |
| bth0xsp46xys_Treg_APC_ratio | 1.56 | 0.0234 | 1.06 | 2.30 | 0.50 | 0.56 | 0.06 | 0.0232 |
| i3ew4evg5a4d_A_proportion | 1.79 | 0.0261 | 1.07 | 3.00 | 0.50 | 0.57 | 0.07 | 0.0303 |
| yldxliwbr70c_rel_abundance_M | 1.54 | 0.0278 | 1.05 | 2.25 | 0.50 | 0.55 | 0.05 | 0.0303 |
| jwpurc1xc2xt_MEAN_COMPACTNESS_MF | 0.63 | 0.0289 | 0.41 | 0.95 | 0.50 | 0.53 | 0.03 | 0.0351 |
| 144ijpego00z_overall_interaction_score | 0.66 | 0.0328 | 0.45 | 0.97 | 0.50 | 0.56 | 0.06 | 0.0319 |
| m8693e5z9j3h_Fraction_F_within_50um_of_TU | 0.62 | 0.0344 | 0.39 | 0.96 | 0.50 | 0.56 | 0.06 | 0.0276 |
| 9d00z9xtifzo_F_density_180_200 | 0.63 | 0.0356 | 0.41 | 0.97 | 0.50 | 0.56 | 0.06 | 0.0299 |
| mc5dli1azo3k_TU_perimeter_velocity | 1.59 | 0.0373 | 1.03 | 2.45 | 0.50 | 0.55 | 0.05 | 0.0308 |
| 1v6365tdzyws_myofibroblast_pdpn_adjacency_ratio | 1.64 | 0.0401 | 1.02 | 2.64 | 0.50 | 0.55 | 0.05 | 0.0321 |
| 9mp42ayyvnj7_Th_correlation_coefficient | 1.71 | 0.0402 | 1.02 | 2.87 | 0.50 | 0.55 | 0.05 | 0.0321 |
| 7a3yg8qpno81_COMBINED_STROMAL_FD_INDEX | 1.47 | 0.0484 | 1.00 | 2.16 | 0.50 | 0.55 | 0.05 | 0.0522 |
| pbyvmdvdrh87_mean_angle_consistency | 0.63 | 0.0486 | 0.40 | 1.00 | 0.50 | 0.55 | 0.05 | 0.0400 |

**Suppl. Figure 10B**

FDR
Please specify the set of tests over which FDR was applied (e.g., per tumour type and endpoint, and whether continuous and dichotomised variables were included).

**Response:**

**This information is described and now additionally clarified in more detail in the Materials and Methods section (Page 27). Briefly, we applied FDR correction to both categorical and continuous variables during the univariate analysis. Parameters that did not pass the FDR correction step were excluded from subsequent multivariate analysis in the exploration cohort, even if they were nominally significant in the univariate analysis. All tests were performed separately for each endpoint and for each individual parameter.**

Redundancy at the patient level.
Decorrelation appears to be done at the patch/region level, while outcomes are patient level after aggregation. Please also check and report redundancy after patient-level aggregation, to ensure retained features are not effectively duplicates for prognosis.

**Response:**

**We applied rigorous measures during initial experiments to prevent redundancy at the patient level.**

**Two steps of decorrelation were performed:**

**1. “Technical” decorrelation. This was applied at the level of direct, regional measurements, excluding parameters that effectively measure the same features or**

**yield highly similar, dependent values. We used a correlation threshold of > 0.7 for exclusion, which is relatively stringent, as our goal was to retain only the most important parameters.**

**2. Prognostic-level decorrelation. In all prognostic analyses, an additional step was applied at the level of prognostic group stratification. After selecting the optimal thresholds, each pair of parameters was compared based on the similarity of the prognostic stratification they produced in the exploration cohort. The Jaccard score was used for this comparison, and parameters yielding a Jaccard score > 0.85 were considered redundant and filtered out. This effectively means that, for a cohort of 100 patients, the prognostic group assignments produced by the two parameters should differ in approximately 15 patients. We consider this a reasonable threshold to remove any clinically meaningful redundancy.**

**This information is provided in the Materials and Methods (technical decorrelation Page 26, prognostic decorrelation Page 28) section.**

Spatial biology section
Given there is no independent test cohort here, please include an internal, leakage-free validation where all tuning/selection is kept separate from evaluation (e.g., a form of cross-validation that isolates tuning from testing) and report how stable features are.

**Response:**

**As mentioned above, we have now performed cross-validation to demonstrate the validity of the parameters. However, in the main figures, we would like to retain the initial presentation of results, as these primarily aim to illustrate the feasibility and exploratory potential of the approach, and may have greater downstream relevance for researchers interested in applying this information (due to higher statistical power).**

Temporal ordering from static images.
For the temporal claims, please (i) define precisely how “A precedes B” is determined, and (ii) clarify how multiple testing was controlled across the full set of A–B pairs and A–B–C chains. As written. I am unsure if this section is hypothesis generating or meant to test.

**Response:**

**This section demonstrates how SPARK can be applied for advanced hypothesis generation. We consider experimental validation to be beyond the scope of this study, as it would require methods such as spatial biology (e.g., multiplexed imaging), spatial or single-cell RNA sequencing, and multi-regional DNA sequencing. Researchers in each disease domain (e.g., breast cancer) can use this resource as a library to surface and test biologically relevant concepts.**

**We define the concept of “A precedes B” precisely in the Materials and Methods section. More broadly, this corresponds to the well-known biological and pathological concept of a field effect, where an initial event creates a permissive “field” that enables subsequent events to occur. In our statistical framework, this implies that B depends on A and tends to emerge in regions where A is already present. This relationship is enforced through several simple filters - directionality, necessity, and sufficiency - all described in detail in the Materials and Methods section.**

**We apply a form of false discovery rate (FDR) control indirectly in this analysis. Specifically, we require that A–B and A–B–C event chains show a clear association with tumor aggressiveness or prognosis. We then implement a stringent FDR-like filtering procedure that considers both p-values and effect sizes, retaining only the most significant sequences.**
**Specifically, for each identified chain, we compute two categories of metrics comparing patient sub-cohorts with and without the chain:**
**1. Event frequency ratios — e.g., p_{N1} / p_{N0} and the ratio of UICC pathological Stage III–IV to Stage I–II;**
**2. Relative risk ratios at 36 and 60 months for overall survival (OS), cancer-specific survival (CSS), and progression-free survival (PFS).**

**We apply stringent inclusion thresholds to retain only highly significant chains:**

- **A minimum ratio of 5.0 for pathological variables, and**
- **A minimum relative risk ratio of 2.0.**

**We relax this to 3.0 and 1.5 for LUSC as having only one prognostic endpoint well characterized.**

**These thresholds are heuristic yet stringent in our opinion, intended to yield a comparable number of top-significant parameters across tumor types rather than to serve as robust cutoffs.**

**Chains are included in downstream analyses if at least two of these ratios exceed the threshold, except in colorectal cancer, where - similar to our prognostic analysis - the number of significant parameters is high. In this case, only chains with more than four significant parameters are retained for further consideration.**

**Although this is not a classical FDR correction, we believe that the measures applied effectively control for false discoveries by prioritizing the most robust and biologically meaningful associations.**

“No training.”
Please temper phrasing: SPARK may not require additional training to generate parameters, but MSI with XGBoost is trained, and upstream QC/segmentation/cell-typing models are pre-trained which influence results.

**Response:**

**We agree that the quality of the outputs may depend on the quality of the upstream algorithms. However, there are several important considerations in this regard:**

**1) The precision of the algorithms used in our study is very high and has been validated in rigorously designed studies involving both formal/clinical validation and independent real-world datasets - for example, for tissue and artifact detection (GrandQC tool; Weng Z. et al., Nat. Commun. 2024, [doi:10.1038/s41467-024-54769-y]), lung adenocarcinoma and lung squamous cell carcinoma classification (Kludt C. et al., Cell Rep. Med. 2024, [doi:10.1016/j.xcrm.2024.101697]), and colorectal cancer analysis (Griem J. et al., Mod. Pathol. 2023, [doi:10.1016/j.modpat.2023.100327]).**

**We have made the widely used GrandQC tool - an essential preprocessing component - fully open source, along with several datasets containing precise manual annotations for training segmentation models in lung and colorectal cancer. Moreover, new publicly available datasets for model training continue to appear each month, further enhancing the robustness and reproducibility of this research field.**

**2. Many research groups and industry stakeholders (e.g., pharmaceutical and biotechnological companies) are actively developing and using highly capable segmentation tools and cell detection algorithms. Several high-quality datasets and pretrained models for cell detection and classification are publicly available, including Lizard, PanNuke, CellViT, HoverNeXt, and HistoPlus, as well as proprietary solutions from commercial entities such as PathAI and Aignostics.**

**We regard SPARK as a foundational principle for data exploration - not only within academia but also in the broader context of biomedical and pharmaceutical research, where high-precision upstream algorithms are already established. We see particularly strong potential in immuno-oncology, where SPARK could facilitate the discovery of explainable and interpretable biomarkers to support the personalization of patient therapy.**

**3. Recently, research in pathology has clearly shifted toward foundational models, which have attracted significant attention due to the relative ease of building tools based on them. Although these models play an important role as components within more complex algorithms, they still lack key capabilities such as explainability and reasoning, and their limitations—such as training biases that restrict generalization—are likely underestimated (e.g., de Jong E.D. et al., 2025; arXiv:2501.18055). As this becomes increasingly recognized (a point we aim to emphasize through SPARK), greater effort can be directed toward developing segmentation and classification backbones that first parse tissue into interpretable objects, characterize these objects, and subsequently build more complex models upon this structured information. This approach, rather than relying on raw H&E images with their inherently high complexity and information density - even for the most advanced foundational models - may enable the creation of more sophisticated tools and algorithms that can serve as upstream components within the SPARK framework.**

Calibration and clinical utility.
Where you present multivariable Cox/predictors, if available please report calibration (e.g., calibration slope/Brier) and decision-curve analyses on external/LOCO cohorts, and quantify improvement over clinical baselines.

**Response:**

**We report model calibration as additional information for all multivariate models, using the calibration slope as a metric. This information is provided in Supplementary Tables 30,32,34,36,38 alongside the main results. In the vast majority of models, the calibration slope is close to 1, indicating good agreement between predicted and observed outcomes.**

**We also compute the C-index for both the baseline models (including only clinico-pathological variables) and all multivariate models, and report the ΔC-index (difference between the two) along with the the Likelihood Ratio Test (LRT) p-value. We observe strong concordance between the significance of the p-values—reflecting the added predictive value beyond baseline models—and the p-values from the corresponding multivariate Cox models. This information is likewise included in the same Supplementary Tables.**

Batch / stain / site effects.
I assume slides vary by scanner/stain across centres (TCGA, PLCO, NLST, UKK, UKE, HAL). Please clarify any harmonisation or, if none, provide brief sensitivity analyses to show findings are not driven by site/batch. (Similarly for IMC/TMA batches.)

**Response:**

**Thank you for raising this important point.**

**Batch effects are natural to pathology image data and are largely unavoidable, as they stem from multiple stages of sample handling and imaging. However, they should always be properly accounted for during model development and validation.**

**Common sources of batch effects include:**

- **Grossing principles – for example, whether the front or only the central part of the tumor was sampled.**
- **Laboratory practices – variations in tissue cutting, mounting, and staining that can introduce artifacts.**
- **Digitization – differences between scanning systems, which can result in out-of-focus artifacts and scanner-specific color profiles.**

**In addition, clinical, selection, center-specific, and country-specific biases can significantly influence single retrospective cohorts.**

**Robust parameters should generalize well despite the minimal to moderate domain shifts that are intrinsic to pathology data.**

**SPARK incorporates a rigorous quality control (QC) algorithm that effectively excludes significant artifacts—one of the primary sources of instability. The QC is performed by the GrandQC algorithm (Weng Z. Nat. Commun., 2024), which we have fully open-sourced and made publicly available.**

**Our upstream segmentation models, as noted above, have demonstrated strong generalization performance in independent, multi-centric validation studies specifically designed to capture cross-institutional biases (as detailed in our previous publications). These aspects are a consistent and essential part of our validation strategy and were explicitly optimized for during training.**

**That said, we acknowledge that some degree of technical variability remains inherent to even the most precise pathology image analysis algorithms. For instance, the smoothness of boundary detection of different structures (tissue, cells) can be affected by subtle image quality issues - such as sub-perceptible, pixel-level, scanner-specific blurring - which may lead to minor batch effects in the output parameters. <u>However, we view this not as a flaw, but as a natural characteristic of digital pathology research conditions.</u>**

**Nevertheless, in this revision - prepared in response to your comment - we address in detail another controllable source of instability, namely, the SPARK-generated parameters themselves, which may exhibit cohort-specific variations.**

**As mentioned in the original submission, we originally identified certain parameters that can contribute to cross-center instability or batch effects (initially discussed in the Limitations section). These parameters primarily involve analyses of color or pixel intensity values without normalization, which are more prone to variability across centers. Based on your recommendation, we now address this issue systematically to demonstrate that the validity of our results is not compromised by batch effects.**

**Specifically, for each tumor type, we review all parameters that successfully passed through the stages of univariate and multivariate Cox analysis analysis in the**

**exploration cohort and multivariate analysis in test cohort, in a version without FDR correction (i.e., larger list; corresponding to Figure 4e).**

**Given the natural heterogeneity of cohorts (and, respectively, of analysis results) and potential selection biases at the cohort level (for instance, a higher proportion of high-risk patients due to center-specific recruitment practices or clinical characteristics), it is not always possible to distinguish whether observed parameter differences reflect true biological variation or technical batch effects. To address this, we apply a combination of statistical evaluation and manual review to ensure robust assessment and interpretation.**

**From a statistical perspective, we analyzed the distributions of predicted risk groups during dichotomization (e.g., in cohort 1, 35% of patients were predicted as high-risk, whereas in cohort 2, 50% were predicted as high-risk based on a single parameter). We also examined the case-level parameter distributions within each cohort of a given tumor type, including mean, median, maximum, standard deviation, and quantiles, as well as cross-cohort summaries (global median, global median absolute deviation [MAD], and the largest difference between two cohort medians, including its MAD-normalized counterpart, with latter being one the most helpful parameters at the higher level).**

**Each parameter was then manually reviewed, beginning with those that exhibited substantial discrepancies - specifically, parameters where at least twice as many patients were classified as high-risk in one cohort compared to another, or where the MAD-normalized largest inter-median difference exceeded 2.**

**Through this process, we identified a subset of parameters likely influenced by batch effects, including only few that demonstrated clearly detrimental batch behavior (for example, cases where one cohort showed 70% of patients classified as high-risk, while another showed 0%).**

**In most cases, batch effects were evident in only one out of the two to four test cohorts. Nevertheless, we considered such parameters to be batch effect-vulnerable and excluded them from the updated analysis, as described below, even if they can be included into validation using one or more cohorts (where batch effects were not evident):**

**Parameters vulnerable to batch effects identified among initially validated significant categorical and continuous parameters (larger list of significant parameters without FDR correction, corresponding to Figure 4e).**

| Cohort | Categorical parameters, n | from total parameter n: | Continuous parameters n | from total parameter n: |
|---|---|---|---|---|
| LUAD | 19 from 16 ideas | 162 | 20 from 17 ideas | 81 |
| LUSC | 41 from 35 ideas | 107 | 7 from 7 ideas | 47 |
| COAD | 27 from 22 ideas | 110 | 10 from 10 ideas | 107 |
| BRCA | 6 from 5 ideas | 83 | 3 from 2 ideas | 46 |
| HNSC | 0 from 0 ideas | 22 | 5 from 5 ideas | 55 |

**Although some parameters are vulnerable to batch effects, they represent minor part from the whole number of identified significant parameters with independent validation in test cohorts (with LUSC affected more than other tumor types).**

**Again, we would like to emphasize, that although for purity we consecutively exclude those from the presented results in Figures (updated are now all panels Figures 4e-4t), they do show significance in independent validation (batch effects affect mostly one cohort of many) and are relevant, however, should be handled with caution (see discussion below). Many of the affected parameters were filtered out via FDR correction (update of numbers of parameters for FDR corrected list is presented in Figure 4f). Only single parameters from the top performing parameters were affected not changing the results significantly (obviously non-batch affected parameters from the beginning naturally show higher levels of significance).**

**For the multi-parametric prognostic systems, the results are either unchanged or, in some cases (BRCA), improved - since the exclusion of batch effect–vulnerable parameters led to even better risk stratification performance (updated panels Figure 4m-t).**

**Two important aspects should be highlighted:**

**1. We still consider these features to be of substantial importance (they also passed a multi-step statistical selection process in a single-cohort setup), even though their technical implementation is challenging.**

**All identified parameters affected by batch effects follow the same pattern — they measure absolute pixel intensities (e.g., chromatin density in cells or cytoplasmic coloration) without additional normalization. In contrast, parameters that capture variability of pixel intensities rather than absolute values are not affected.**

**Currently, there is no viable approach to improve interoperability in the pathology domain, i.e., to achieve effective normalization across datasets.**

**Moreover, <u>we propose a clear strategy for potential end users on how these features can be implemented in real clinical use cases</u>:**

- **Scanning on the same scanner to minimize inter-device variability.**
- **Stain normalization (as an additional preprocessing step, e.g., using Macenko or Reinhard algorithms) can mitigate moderate staining variations, though it may not fully correct for severely over- or under-stained slides.**
- **(Recommended) Cohort-level normalization using z-score or ComBat-based approaches to ensure cross-cohort comparability.**

**Therefore:**
**a. we don't use them in presentation in Figures and clearly state so.**
**b. we don't exclude them from Suppl. Figures. We introduce additional column "Batch_effect_detected" and highlight such parameters), as shown below:**

**Table S36. Breast carcinoma: independent validation of prognostic parameters (categorical, FDR correction)**

| Parameter | Batch_effect_detected | OS_HR | OS_P | OS_THR | OS_CI_LOW | OS_CI_HIGH | OS_N | OS_CINDEX_BASE | OS_CINDEX_BASE_CI_LOW | OS_CINDEX_BASE_CI_HIGH |
|---|---|---|---|---|---|---|---|---|---|---|
| 0fuzp53opov4_CYTOPLASM_BASOPHILIA_VARIANCE_MAX | | 0.806138879 | 0.184610483 | 0.0294834 | 0.586335571 | 1.108341238 | 178 | 0.680579361 | 0.636522063 | 0.722294166 |
| 1i39mtnj4oa6_MACRO_SPECKLE_CONTRAST_VAR_MAX | | 1.219121356 | 0.277425393 | 52047.06787 | 0.852649642 | 1.743103858 | 196 | 0.669624153 | 0.630362742 | 0.71312353 |
| 1iv4oahx25zs_LYMPHO_ELONG_DISP_MIN | | 0.907290523 | 0.587582763 | 0.7568342 | 0.638331849 | 1.289573901 | 196 | 0.669624153 | 0.630362742 | 0.71312353 |
| 1iv4oahx25zs_PLASMA_ELONG_DISP_MIN | | 0.943185663 | 0.729590106 | 0.0328255 | 0.67695921 | 1.314110485 | 178 | 0.680579361 | 0.636522063 | 0.722294166 |
| 1iv4oahx25zs_PLASMA_ELONG_DISP_MAX | | 1.222313451 | 0.206583962 | 1.3746722 | 0.895140898 | 1.669067043 | 178 | 0.680579361 | 0.636522063 | 0.722294166 |
| 1iv4oahx25zs_NEUTRO_ELONG_DISP_MIN | | 0.721756468 | 0.092590288 | 0.0127057 | 0.493592376 | 1.05538988 | 148 | 0.685737472 | 0.639997415 | 0.73101373 |
| 1iv4oahx25zs_EOSINO_ELONG_DISP_MIN | | 0.800757084 | 0.192885668 | 0.1280368 | 0.573120384 | 1.118808414 | 182 | 0.67717582 | 0.633972024 | 0.720371028 |
| 1my2c4o8jszj_SD_SPECKLE_INDEX_MIN | X | 0.589696232 | 0.019358299 | 16.2814274 | 0.378782749 | 0.918050376 | 196 | 0.669624153 | 0.630362742 | 0.71312353 |
| 1my2c4o8jszj_MAX_SPECKLE_INDEX_MIN | X | 0.845328847 | 0.368067104 | 122.1644121 | 0.586306099 | 1.218784627 | 196 | 0.669624153 | 0.630362742 | 0.71312353 |
| 1n5felm8naxy_FIBROBLAST_SPINDLE_INDEX_MIN_MIN | | 0.840415501 | 0.261169504 | 1.0076971 | 0.62056616 | 1.138151353 | 196 | 0.669624153 | 0.630362742 | 0.71312353 |
| 1obt4qu2bans_AVG_CLUSTERING_COEFF_MIN | | 0.735514419 | 0.057525826 | 0.0351992 | 0.535699016 | 1.009860844 | 196 | 0.669624153 | 0.630362742 | 0.71312353 |
| 1obt4qu2bans_AVG_CLUSTERING_COEFF_MEAN | | 0.761275139 | 0.096863897 | 0.0517465 | 0.551694817 | 1.050471782 | 196 | 0.669624153 | 0.630362742 | 0.71312353 |
| 1qmkdkato8bq_HYPOXIA_SCORE_TUMOR_MEAN | | 0.865229121 | 0.431962959 | 0.5361981 | 0.603016575 | 1.241460786 | 196 | 0.669624153 | 0.630362742 | 0.71312353 |
| 1smqxig1fjic_NE_RATIO_MAX | | 0.823666198 | 0.551420047 | 8 | 0.435037026 | 1.559467273 | 160 | 0.671973511 | 0.62588342 | 0.715683724 |
| 1smqxig1fjic_NE_RATIO_MEAN | | 0.922550156 | 0.642353188 | 0.3853809 | 0.65650269 | 1.296413257 | 160 | 0.671973511 | 0.62588342 | 0.715683724 |
| 210pdq3rjftr_SD_CHROMATIN_CLUMPING_MIN | | 0.934544748 | 0.652146848 | 21.3339214 | 0.696260299 | 1.25437841 | 196 | 0.669624153 | 0.630362742 | 0.71312353 |
| 210pdq3rjftr_MIN_CHROMATIN_CLUMPING_MIN | | 0.944384676 | 0.802899452 | 16.6666667 | 0.602566696 | 1.480105723 | 196 | 0.669624153 | 0.630362742 | 0.71312353 |
| 29px7lf5a3wx_INNER_LAYER_SLACK_MAX | | 2.253831636 | 2.15E-07 | 7745.966458 | 1.657812346 | 3.064132714 | 196 | 0.669624153 | 0.630362742 | 0.71312353 |
| 29px7lf5a3wx_INNER_LAYER_SLACK_MEAN | | | | | | | | | | |
| 29px7lf5a3wx_MIDDLE_LAYER_SLACK_MIN | | | | | | | | | | |
| 29px7lf5a3wx_MIDDLE_LAYER_SLACK_MAX | | 1.449248736 | 0.023054143 | 25494.64812 | 1.05235953 | 1.995821618 | 196 | 0.669624153 | 0.630362742 | 0.71312353 |
| 29px7lf5a3wx_MIDDLE_LAYER_SLACK_MEAN | | 0.946818831 | 0.722868339 | 5808.643841 | 0.700000415 | 1.28066481 | 196 | 0.669624153 | 0.630362742 | 0.71312353 |
| 2iewr5vwzbnt_CONTACT_PROBABILITY_MAX | | 1.13934439 | 0.485359382 | 0.033224 | 0.789774183 | 1.64364152 | 196 | 0.669624153 | 0.630362742 | 0.71312353 |
| 2pbeobp6dvn1_MIN_NUCLEAR_AREA_MAX | | | | | | | | | | |
| 2pbeobp6dvn1_MIN_NUCLEAR_AREA_MEAN | | 1.002908683 | 0.984210579 | 4.3361472 | 0.752208234 | 1.337164075 | 196 | 0.669624153 | 0.630362742 | 0.71312353 |
| 2txrpjgl75eu_ENTROPY_STD_MAX | | 1.225469202 | 0.224669641 | 0.4575632 | 0.882602236 | 1.701530658 | 196 | 0.669624153 | 0.630362742 | 0.71312353 |
| 2txrpjgl75eu_ENTROPY_STD_MEAN | | 0.611027471 | 0.003024602 | 0.3089462 | 0.441215276 | 0.846195929 | 196 | 0.669624153 | 0.630362742 | 0.71312353 |
| 32v2q5w03ghp_EOSINO_AREA_MEAN_MIN | | 0.784272966 | 0.155940066 | 6.875 | 0.560644901 | 1.097101006 | 190 | 0.670778161 | 0.631299976 | 0.70978807 |

**Crop of Table S36**

**2. We have adapted our parameter verification script to a <u>new parameter analysis tool</u> that enables large-scale parameter evaluation with respect to specific research questions.**

**In this context, we generated a comprehensive list of parameters (including those not previously validated for prognostic relevance) that may be affected by batch effects.**

**<u>We will make this parameter list, along with best-practice guidelines and the parameter screening tool, publicly available as part of our GitHub repository (also included in Table S3 of the manuscript).</u>**

**Inclusion in this list does not necessarily imply that batch effects are expected; it only indicates that pixel intensities are involved and that additional control might therefore be important. In many cases, explicit or implicit normalization is already applied - for instance, when measurements focus not on absolute intensity values but on relative variations, such as chromatin distribution heterogeneity or similar features.**

**Table S3. Full list of parameters before decorrelation (Use cases 1 and 2)**

| Parameter name \| [IDEA NAME]_[PARAMETER NAME] | Works with pixel intensities (consider normalization / control of batch effects) |
|---|---|
| 094tbl12fffo_MEAN_CYTOPLASMIC_BASOPHILIA | x |
| 094tbl12fffo_SD_CYTOPLASMIC_BASOPHILIA | x |
| 09oganc9lq1b_GRADIENT_FIBROBLAST_RESPONSE | |
| 09oganc9lq1b_GRADIENT_INTERCEPT | |
| 09oganc9lq1b_GRADIENT_R_SQUARED | |
| 09oganc9lq1b_MEAN_FIBROBLAST_DENSITY | |
| 09oganc9lq1b_MEAN_TUMOR_GRADIENT | |
| 09oganc9lq1b_MAX_TUMOR_GRADIENT | |
| 09t5d9fkl0s6_MEAN_TUMOR_TO_OTHER_RATIO | |
| 09t5d9fkl0s6_SD_TUMOR_TO_OTHER_RATIO | |
| 09t5d9fkl0s6_MIN_TUMOR_TO_OTHER_RATIO | |
| 09t5d9fkl0s6_MAX_TUMOR_TO_OTHER_RATIO | |
| 0deksrl5dju0_TNP_RATIO | |
| 0fuzp53opov4_CYTOPLASM_BASOPHILIA_VARIANCE | x |
| 0fuzp53opov4_CYTOPLASM_BASOPHILIA_MEAN | x |
| 0fuzp53opov4_CYTOPLASM_BASOPHILIA_STD | x |
| 0gyycvnhqc3z_VARIANCE_NT_RATIO | x |
| 0qvjnks544pk_TUMOR_TO_IMMUNE_RATIO | |
| 0slldfjtvc0p_CO_CLUSTERING_INDEX | |

**Crop of Table S3**

**3. Correlation analysis and predictive analysis.**

**The results presented here (particularly the predictive analyses) pertain to single cohorts; therefore, we do not explicitly remove any parameters, even if they could**

**potentially be vulnerable to batch effects. This issue becomes evident mainly in prognostic analyses using merged cohorts, as the reviewer correctly notes.**

**We have carefully reviewed the SHAP analysis outputs for all predictive models and did not observe the inclusion of color-based parameters previously identified and excluded. Most parameters contributing to the ER, MSI, PD-L1, and HPV predictive models describe tumor cell characteristics (e.g., pleomorphism, nuclear size) or microenvironmental features such as composition, spatial distances, ratios, gradients, or cellular orientation - all of which are biologically plausible with regard to tested predictive parameters.**

**4. Temporal sequence analysis.**

**In this analysis, we work with a large number of features selected for univariate significance (details of FDR-like procedures are provided below in response to your other comment), with an initial set of >900 parameters. For all tumor types we use test cohorts, which are more representative—typically >1 slide per tumor and less selection bias (e.g., TCGA often favors high-purity tumor regions for NGS).**

**Batch effects may affect results in LUAD, LUSC, and COAD, which combine multiple cohorts. In the BRCA use case we analyze a single cohort, so we leave it as is.**

**A manual review of batch-affected parameters is infeasible given their number, so we apply the statistical pipeline estbalished as described above. Because all cases are binarized into high- and low-risk regions, we compare the distributions of high/low-risk parameters within each cohort at the case level and compute summary metrics. As noted above, the most informative metrics are the median, the global median absolute deviation (MAD), and – especially - the largest inter-cohort median difference normalized by the global MAD. This last quantity captures how far apart cohorts are in units of robust global variability.**

**We use the following thresholds for interpretation of the MAD-normalized inter-median difference:**

**< 1: Cohort medians differ less than 1 MAD (≈ within normal variation)**
**~1–2: Moderate shift; possible mild batch imbalance (tolerable)**
**> 2: Strong separation; clear cohort-dependent shift**
**> 3: Very strong / likely systematic difference**

**We exclude all parameters with a MAD-normalized inter-median difference >2, removing 110 parameters in COAD, 302 in LUAD, and 286 in LUSC from the 906 initially included. For additional robustness, we exclude the small UKE sub-cohort in both LUAD and LUSC - which alone accounted for batch effects in a number of further parameters - retaining the merged PLCO, NLST, and UKK sub-cohorts in both tumor types.**

**We then repeat the experiments and update all figures. We observe substantial overlap (≈80%) among the top A, B, and C parameters across tumors (with LUSC somewhat more affected). We find no new results; the main conclusions from the initial experiments remain unchanged. Our review indicates that many batch-affected parameters had already been removed during the original selection process.**

**The fully updated results are presented in Fig. 6 and Extended Data Figs. 9 and 10.**

**The distribution of cell types relevant to each tumor type across stages of tumor development also remains unchanged. The only notable difference is a slightly more pronounced spike in the role of eosinophils in LUSC (see below).**

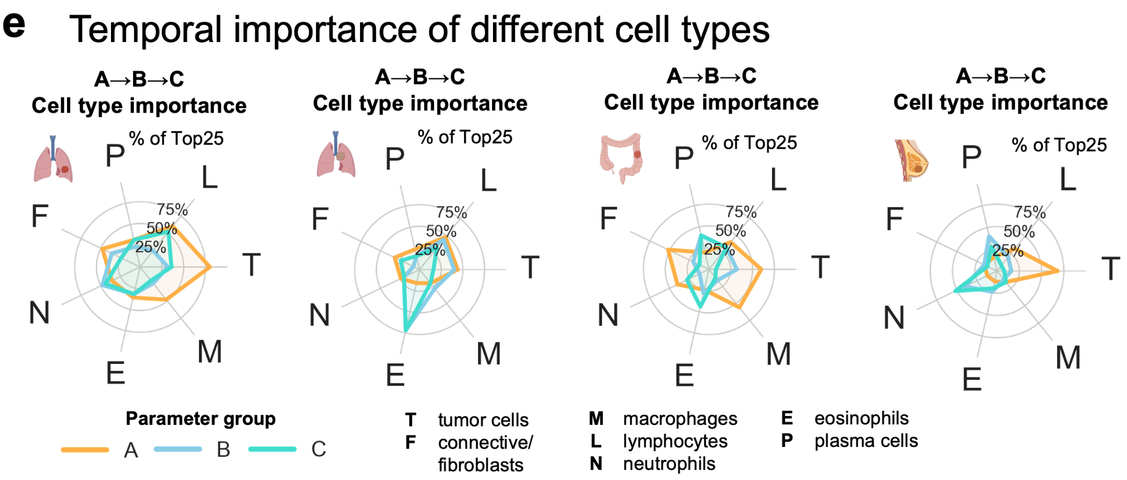


**Fig. 6e – updated results of temporal evolution analysis.**

**##**

**We are not aware of any batch information in the METABRIC cohort. Moreover, we believe this consideration is not applicable in our case, as we do not work with raw images - where batch effects typically arise - but rather with pre-processed, cell-annotated images (i.e., masks). All potential quality issues have already been addressed and filtered out by the authors' pre-processing pipeline (Danenberg et al. Nat Gen 2022; doi [10.1038/s41588-022-01041-y]).**

Segmentation / cell-typing dependencies.
Because downstream features rely on upstream segmentation and cell-typing and the article mentions relabeling to avoid misclassification, is there a check to show the hinge on the relabeling rules?

**Response:**

**Relabeling is performed only in clearly defined situations. All single-cell detections (including those subjected to relabeling) were reviewed and verified by pathologists in our study. The most critical issue for single-cell detection in general involves out-of-focus regions, which are systematically masked during preprocessing by the previously described GrandQC quality control tool in our pipeline.**

**Misclassification statistics are recorded for each slide. In most cases, relabeling affects fewer than 0.01–0.1% of cells. Although all cases were reviewed by pathologists, we consider relabeling exceeding 0.1% of cells a meaningful threshold that should trigger a detailed re-evaluation.**

Patch size & inclusion threshold.
Please briefly justify the 1 mm² patch and the ≥10% tumour-area rule, and show results are robust to reasonable alternatives.

**Response:**

**One important clarification regarding the tumor-area rule: the 10% threshold applies only to the epithelial tumor component, excluding tumor stroma and other associated classes (e.g., necrotic debris, mucin, tertiary lymphoid structures [TLS]) as well as the background.**

**For the region (patch) size, we use 1 mm² as an answer to the question: What is the smallest size of a tumor subclone that can affect patient prognosis? This value represents a consensus among participating expert pathologists.**

**Combined with the 10% tumor-area threshold, the 1 mm² region size also enables the analysis of biopsy samples, should this be considered in future studies.**

**Moreover, tumor percentage primarily relates to calibration, whereas the patch size represents a hyperparameter that may influence results of parameter selection.**

**We conducted additional experiments to confirm that these parameter choices are reasonable and that varying them does not produce substantially different outcomes. Specifically, we tested 20% and 30% tumor-area thresholds, concluding that thresholds above 30% are not meaningful, as even in samples with moderate stromal content, 30% epithelial tumor component typically covers nearly the entire analyzed region.**

**We also evaluated a 0.5 mm² patch size as an only viable alternative in our opinion (given considerations outlined above).**

**In both sets of experiments, we observed substantial overlap in the identified significant parameters within the exploration cohort for LUAD (TCGA), supporting the robustness of our parameter selection.**

**Below is the summary of the results (also included now as a new Extended Data Fig. 4F-H):**

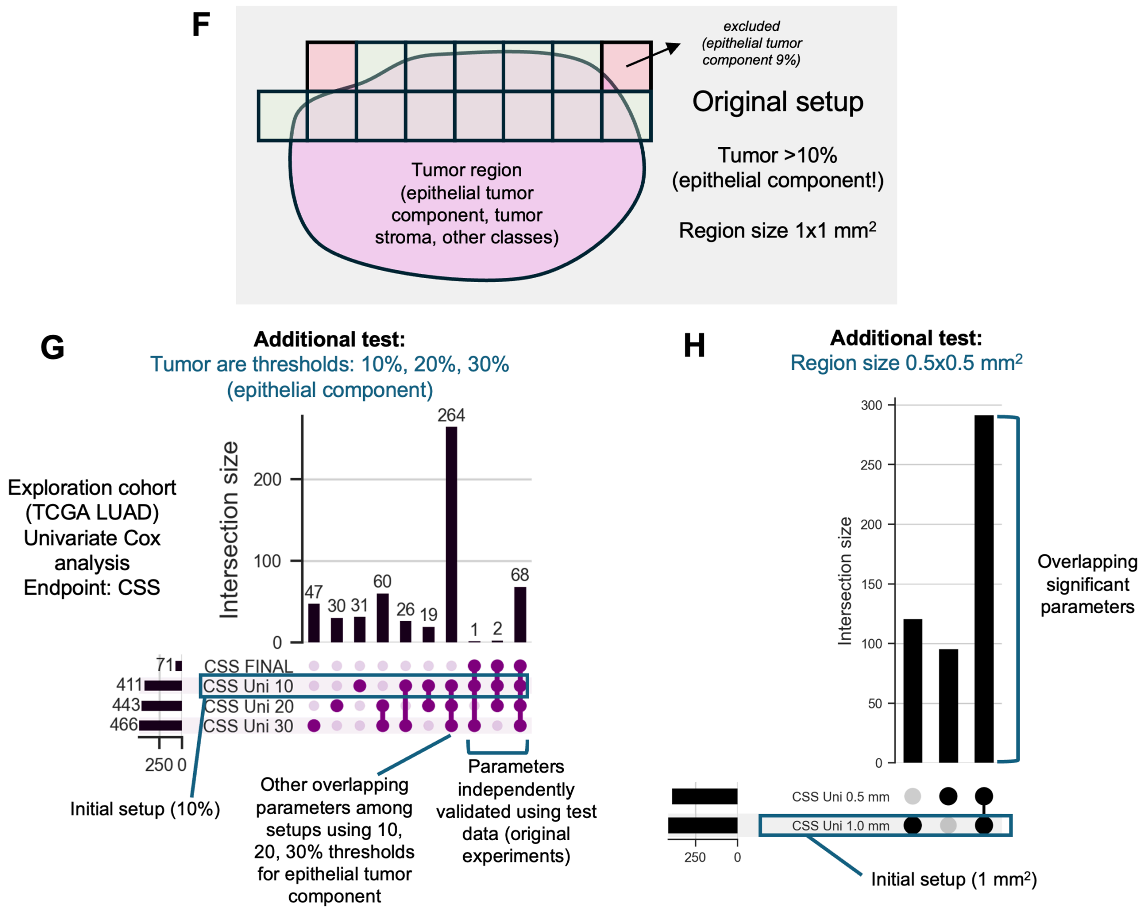


**Extended Data Fig. 4**

**In particular, for the tumor-area thresholds tested (10%, 20%, and 30%), we observed that nearly all parameters identified as independently significant (initial experiments, independent test cohort) overlapped across these three configurations.**

Creativity levels in idea generation.
It is unclear whether “basic → visionary” materially affects outcomes. Please explain the measurable effect of these levels (e.g., diversity or downstream yield).

**Response:**

**The selection of creativity levels was guided by empirical evidence of using LLMs (e.g., ChatGPT/OpenAI or Antropic).**

**During the planning phase of the project (and confirmed again during the current revision), we observed clear effects of prompting large language models (LLMs) with different creativity levels. This observation was further supported by our manual review of generated idea databases. However, such manual evaluation is inherently limited by human cognitive capacity, especially when comparing dozens of ideas.**

**To address this limitation in greater detail, we conducted <u>additional experiments</u> using several open-source models (gpt-oss-120b, DeepSeekR1), each generating ideas across 16 cycles at the same creativity level. We analyzed both the outputs of**

idea generation and the semantic diversity of the outputs, following the methodology described above.

**Below is a representative part of the Suppl. Figure 3 demonstrating the results (only for gpt-oss-120b model).**

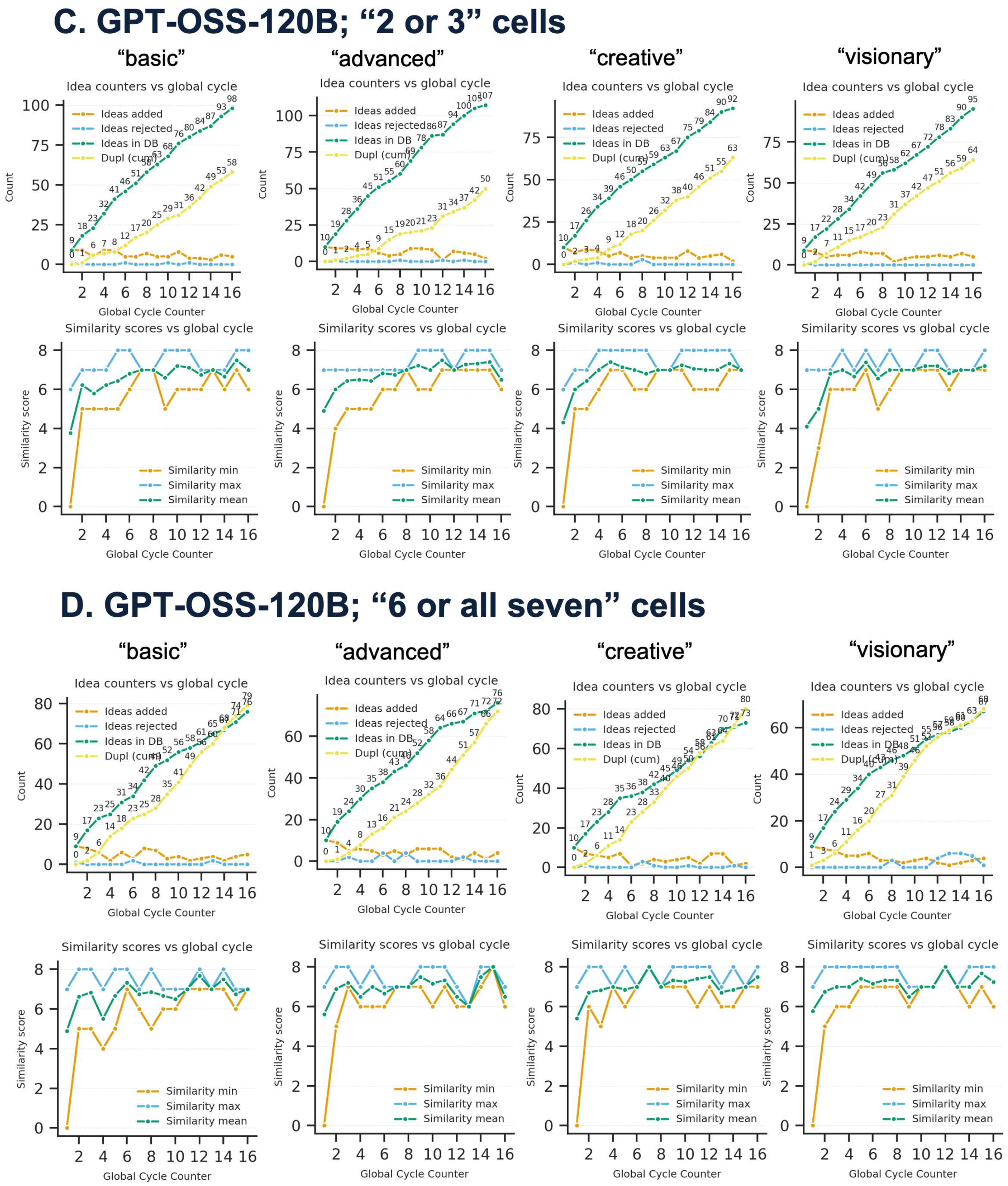


**Suppl. Fig. 3**

**We also plot the semantic diversity of ideas. In brief, we utilize two fields from the idea description - “biological hypothesis” and “analytical/methodological implementation” - to construct a single semantic feature vector. Each field is embedded using a robust biomedical language model (PubMedBERT; output: 768-dimensional embedding vector). Typically, the biological hypothesis field consists of**

a single sentence, while the analytical/methodological implementation field comprises one, occasionally two, sentences. Empirically, and in line with findings from Retrieval-Augmented Generation (RAG) studies, one densely informative sentence appears to represent the optimal unit for comparing ideas.

Accordingly, we generate two embedding vectors - one for each field - and concatenate them for downstream semantic analysis. Technically, this step is straightforward and can be easily adapted to other encoders or semantic analysis frameworks.

The results for the same model are provided below:

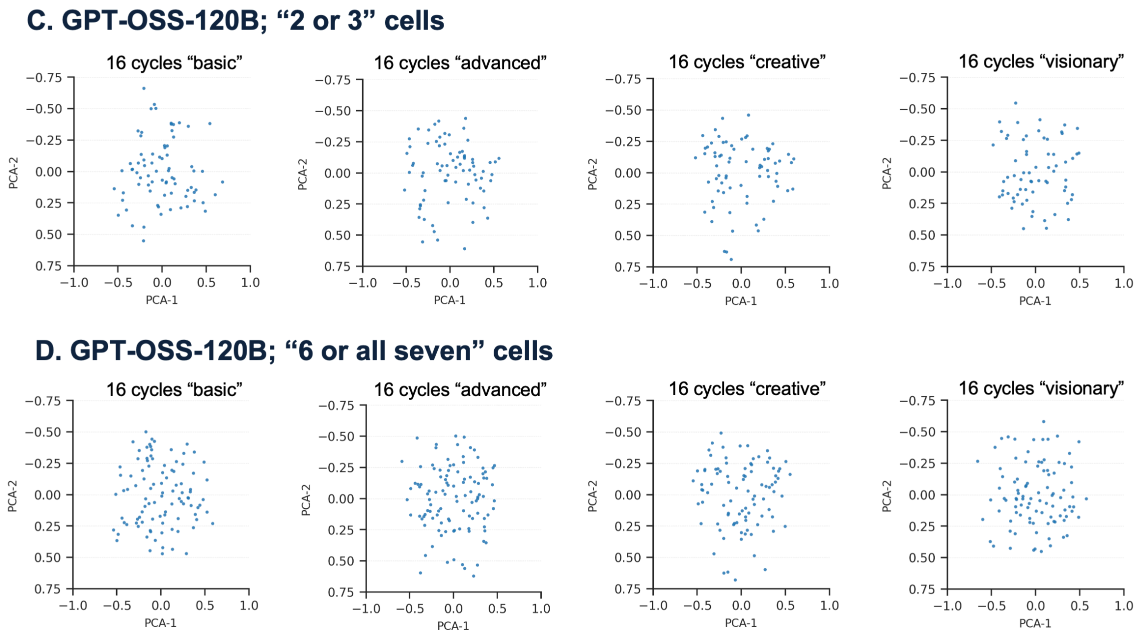


Suppl. Figure 4

We do not observe unambiguous evidence supporting the technical concept of creativity levels in terms of idea yield, number of duplicates, or rejections due to guideline violations. Although the advanced level shows fewer duplicates and a higher number of generated ideas compared to the basic level, comparable evidence for the creative and visionary levels is lacking.

Post hoc, we interpret that most effects of creativity levels apply primarily to generating single ideas in single cycles. However, when the process involves multiple cycles and hundreds of ideas, these effects may become saturated due to the need to generate any divergent ideas that differ from those already present in the database - thus narrowing the potential impact of creativity.

Exactly this was, in fact, our main motivation: when conceptual gaps between individual, already generated ideas become narrower, we increase the model’s creativity level to encourage broader thinking.

Another observation is that we do detect differences between basic and advanced creativity levels in some models, particularly regarding a reduced number of duplicate ideas at the advanced level. Post hoc, we assume that for an LLM, the distinction between terms such as “advanced” and “creative” may be less pronounced.

**Moreover, our clearly defined human concept of four creativity levels was not directly or explicitly communicated to the LLM. The model was not informed that previous ideas had been generated using lower creativity levels, which might involve comparative aspect to boost divergence. A more detailed and explicit task formulation might therefore improve results in the future.**

**In conclusion, although the observed effects may be minimal in highly saturated or exhaustive idea-generation settings, we still perceive subjective differences between outputs at different creativity levels. Given that all LLMs are highly sensitive to linguistic cues and task formulations, we consider our approach valuable and worthy of further refinement.**

**We add these points to discussion (Limitation section, Page 15).**

**> As a side note, we would like to refer to our comprehensive experiments in which the originally used commercial models were replaced with seven open-source models (including both general-purpose and medical-domain–fine-tuned variants). These experiments demonstrate that such models provide an excellent alternative, enabling a fully local SPARK setup without any LLM-related costs. The results are best presented and discussed as the response to the comment of the Reviewer 2 (also Suppl. Figures 1,2 for idea genereation, and 4e for coding modules).**

ORIGINALITY & SIGNIFICANCE
Elements of the concept seem to exist exist (assistant/agent systems, feature-based pathology, multi-agent slide analysis), but the combination and scale appear novel. Related items for positioning:
Pathology assistants/foundation-model pipelines and multi-agent approaches (e.g., conceptually related systems):
https://pmc.ncbi.nlm.nih.gov/articles/PMC11799676/ ,
https://www.nature.com/articles/s41586-024-07618-3 , https://arxiv.org/html/2506.20964v1
“Autonomous scientist”-style loops with a GPT interface in other domain (protein/materials): https://arxiv.org/abs/2504.19017 (Side note, this is named “Sparks”. Maybe consider renaming to avoid confusion?
The claim “single-cell detection is limited to single parameters by humans” seems incorrect (albeit smaller scale than SPARK): https://pmc.ncbi.nlm.nih.gov/articles/PMC12234899/

**Response:**

**The references provided describe foundational and vision-language models (some of which we have already cited). However, these models currently - and likely for the foreseeable future - do not “see” or “analyze” tissue with the level of reasoning and precision demonstrated in our study. We believe that a multimodal approach based on vision–language modeling holds great potential for advancing reasoning; however, this class of models has not yet reached that level of sophistication. Importantly, we consider SPARK’s features to provide a universal framework for automatized generation of highly diverse, image- and outcome-grounded reasoning ground truth for vision–language models - a key bottleneck at present. In this regard, SPARK can facilitate rapid progress in the development of vision–language models.**
**We would also prefer to retain the name SPARK, as the referenced work operates in a completely different domain - protein synthesis. Finally, we have moderated the statement concerning the limitation to single-parameter assessments by human observers and added suggested citation (Page 13).**

Discussion tone
Several phrases read as over-selling for a medical journal audience ("unlimited potential," "new paradigm," "exhaustive descriptors," "fully explainable," "requires no training"). Please moderate language, especially given reliance on pre-trained perception models and at least one trained downstream predictor.

**Response: We moderate the language of the manuscript as suggested.**

CLARITY & TERMINOLOGY
For a non-pathologist, non-computer scientist, this article is hard to read for several reasons.
Abbreviations & cohort mapping.
Define abbreviations like BRCA, LUAD, LUSC, COAD, HNSC, TCGA, PLCO, NLST, UKK, UKE, HAL at first mention. Maybe provide a concise cohort legend. There are several undefined abbreviations and general terms that many readers of Nature Medicine may not know, which should be clarified. The review is already long so I ask you to check this. The above is just examples.

**Response: We provide legend for the tumor types/cohorts (Figure 1f).**

"Parameter."
Example of a term; define parameter explicitly on first use (a computable, numeric image feature derived from an LLM-generated idea; measured on patches/regions; aggregated to the case level).

**Response: We change the text on many occasions accordingly providing definition of parameter in Page 6.**

Data density.
The manuscript is extremely dense (8 main figures but >110 sub-figures; 57 supplementary tables), which makes it hard for non-specialists to navigate. Not sure how to solve this, but maybe consider a hyperlinked parameter book, moving non-essential variants to Supplement—or splitting into focused papers.

**Response: We significantly reduced the density of information moving 40% of plots to extended data and supplements. The number of Figures is reduced to 6.**

CLINICAL UTILITY
If the safeguards above hold and results generalise with calibration and decision-analytic gain beyond clinical baselines, I can see potential utility for explainable risk stratification and feature discovery across imaging tasks. Scope may be limited by the upstream perception models' domains, e.g. imaging, but if it can indeed be generalized beyond pathology I could see it being used for exploration of biomarkers in a lot of complex diseases (such as Alzheimers). However, I temper my expectations for this as my understanding is that it will not be generalizable due to being dependent on unstream pre-training. Maybe a technical reviewer can help clarify.

**Response:**

**We have addressed all of the summarizing comments mentioned here in detail above. We believe that our approach can generalize to multiple disciplines beyond pure pathology (e.g., spatial biology, radiology), as it is essentially unrestricted with respect to the underlying data modality, provided that appropriate perception models are available. While we acknowledge that the extent of generalizability depends on the scope and quality of upstream algorithms (discussed in details above), we see**

**substantial potential given the increasing availability of such tools and the rapid pace of progress in the field.**

Thank you for the opportunity to review.

**Response: Thank you for your valuable comments.**

**Reviewer #4 (Remarks to the Author):**

The submitted manuscript proposes SPARK, a foundational and agentic AI framework for autonomous tumor analysis through reasoning, concept generation, implementation, and data manipulation. It aims to address existing limitations in feature extraction, explainability, and fragmented analysis. The study includes 18 patient cohorts across five malignant tumor types.

In my view, the key features of SPARK are:

1.It helps us understand how tumors grow and become more aggressive over time by analyzing pathology images.
2.It autonomously generates concepts with fully explainable parameters.

I recommend the publication of this work, but I have some concerns that should be addressed before acceptance.

**Response:**

**We sincerely thank the reviewer for the thoughtful and supportive comments, and for highlighting the key strengths and contributions of SPARK.**

Points to address:

• Explain how the generated ideas depend on the model's embedded medical and healthcare knowledge.

**Response:**

**Thank you for this important comment. Indeed, several recent models have been fine-tuned on medical knowledge databases. On the other hand, following the recommendation of Reviewer 2, we conducted comprehensive experiments with open-source large language models, which can serve as an excellent alternative to the initially used proprietary models.**

**At this stage, we are conducting large-scale experiments involving multiple state-of-the-art large language models (LLMs), both general-purpose and medical domain–specific, to evaluate their suitability for the idea-generation step. Importantly, we selected models from different classes, including some that can be executed on low-resource, consumer-grade hardware.**

**A summary of the LLMs used is provided below. Although we do not include very large open-source models (e.g., DeepSeek V3 with 671B parameters or Llama 4 Maverick with 400B parameters), which require HPC clusters for deployment, we acknowledge that some users may have access to such infrastructure. As shown below, scaling laws still apply, and even greater performance can be expected. Our focus, however, is on more realistic deployment scenarios.**

**Table 8. Open-source, locally deployed models used to evaluate reasoning capabilities in the context of idea generation**

| Model | Type | Number of parameters, | Hardware used | Comment |
|---|---|---|---|---|
| Generalist LLMs | | | | |
| DeepSeekR1 | Generalist | 70 b | Server<br>4x A100 80Gb | Llama3-distilled version |
| gpt-oss-120b | Generalist | 120 b | Server<br>4x A100 80Gb | - |
| gpt-oss-20b | Generalist | 20 b | Server<br>2x L40S 48Gb | Can be deployed on MacBook Pro or similar |
| Qwen3:32b | Generalist | 32 b | MacBook Pro<br>M1 Ultra 64Gb RAM | - |
| Medical domain LLMs | | | | |
| Meditron-70B | Medical | 70 b | Server<br>4x A100 80Gb | - |
| MedGemma | Medical | 27 b | MacBook Pro<br>M1 Ultra 64Gb RAM | - |
| BioMistral | Medical | 7 b | MacBook Pro<br>M1 Ultra 64Gb RAM | - |

**For each open-source model in the idea generation task, we used exactly the same setup as in the initial experiments. Although we could have freely used non-commercial models for the idea review and idea duplicate detection subtasks (which are less demanding than idea generation), we chose to use the commercial o3-mini model (OpenAI), as in our initial experiments, to ensure full comparability of the experimental results.**

**The results are summarized in Extended Data Figure 1j (best-performing open-source model), with the complete results provided in Supplementary Figures 1 and 2, also presented below.**

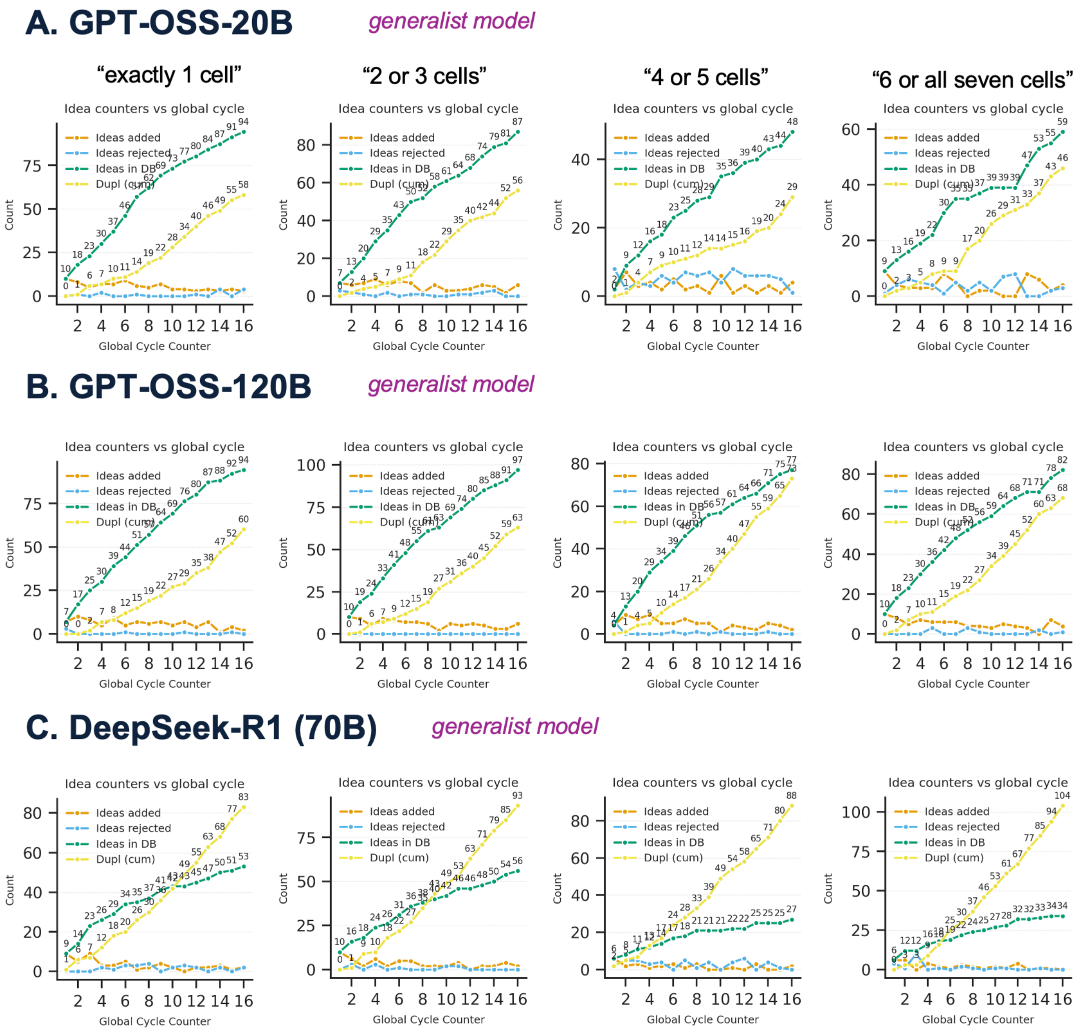
A. GPT-OSS-20B
generalist model
"exactly 1 cell"
"2 or 3 cells"
"4 or 5 cells"
"6 or all seven cells"
B. GPT-OSS-120B
generalist model
C. DeepSeek-R1 (70B)
generalist model
Idea counters vs global cycle
Ideas added
Ideas rejected
Ideas in DB
Dupl (cum)
Count
Global Cycle Counter

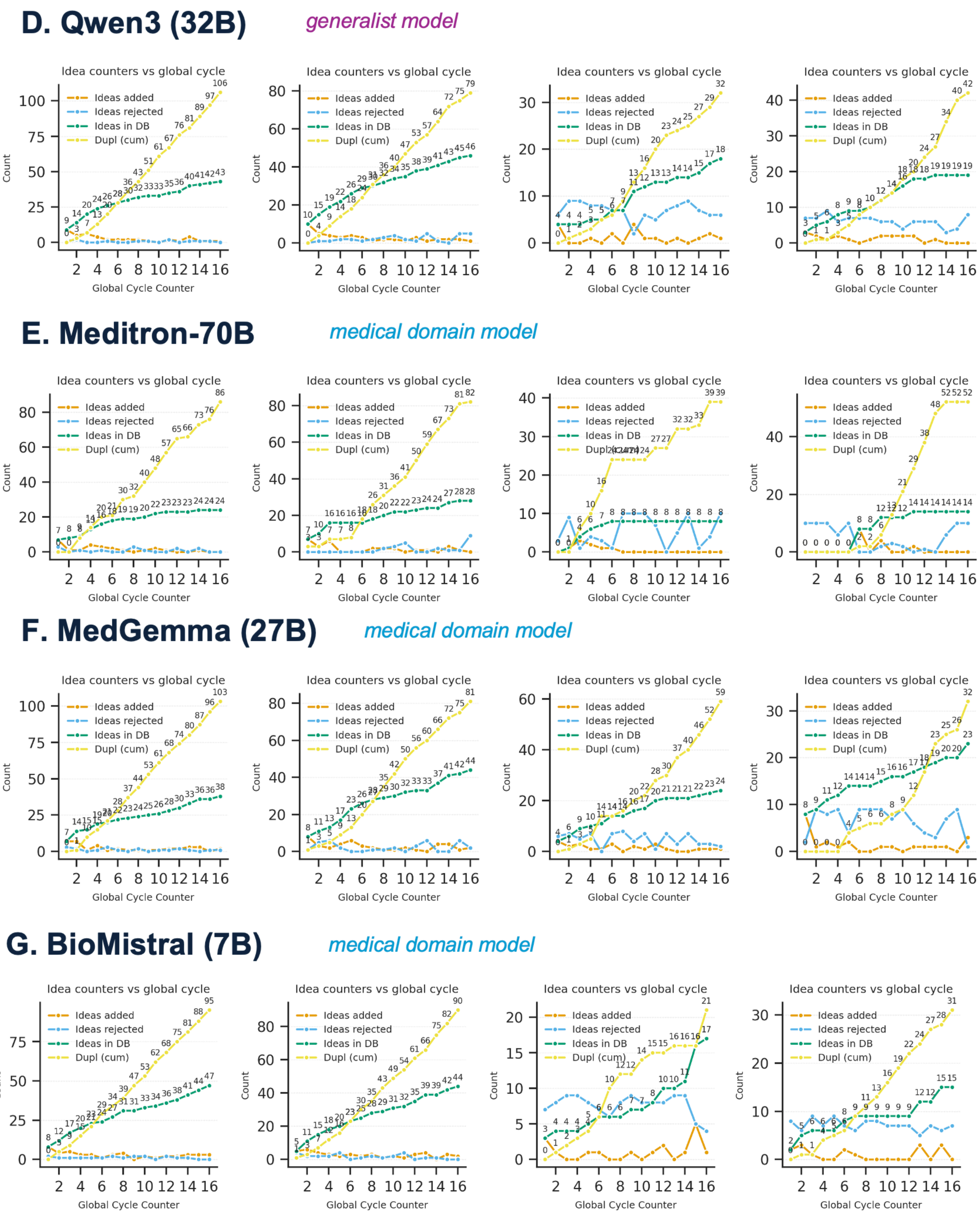


**Suppl. Figure 1.**

**First, we observe notably better results—with more ideas generated, fewer duplicates, and higher compliance with guidelines (i.e., fewer idea rejections due to violations)—for larger models, particularly gpt-oss-120b. This trend also holds for reasoning-oriented models (gpt-oss-120b, gpt-oss-20b, DeepSeekR1 70b with Llama3 distillation) and generalist models. Larger models clearly demonstrate a greater capacity to process and manipulate information, leading to improved performance in dense informational contexts (e.g., when a large number of ideas have already been generated).**

**Second, we did not observe any advantage for models specialized in the medical domain. On the contrary, we had to tighten the task descriptions due to multiple**

additional failures resulting from guideline violations. The most frequent issue was as follows: while medical models were generally able to identify one or a few (2–3) relevant cell types, they consistently struggled to generate more complex ideas involving 4–7 cell types, repeatedly violating the cell number constraint (see the number of rejected ideas in the plots above). These models also produced a substantial number of duplicate ideas with in general low overall yields. Overall, we found no evidence of a competitive advantage in the complexity or diversity of the generated ideas.

We believe that three main factors contribute to this lower performance: (1) most medical models are not reasoning-oriented for now, (2) they are typically smaller in scale (as larger versions are difficult to fine-tune), and (3) their training data are primarily clinically oriented, whereas our tasks focus on tumor microenvironment, tumor biology, and single-cell contexts.

In general, the inferiority of the medical models to generalist models even on medical tasks is a known fact that we confirm in our particular use case. For example, MedGemma (https://arxiv.org/pdf/2507.05201): significantly inferior to o3 in 10/11 medical benchmarks  and in 7/11 medical benchmarks to GPT-4o; BioMistral: (https://arxiv.org/abs/2402.10373) is significantly inferior on 10/10 cinical benchmarks to GPT3.5.

From the perspective of semantic analysis, we observe patterns similar to those found in our initial experiments (Supplementary Figure 2 and below). We conclude that larger and more generalist models are better able to bridge gaps between existing ideas, continuing to identify novel and interesting approaches even in saturated idea spaces.

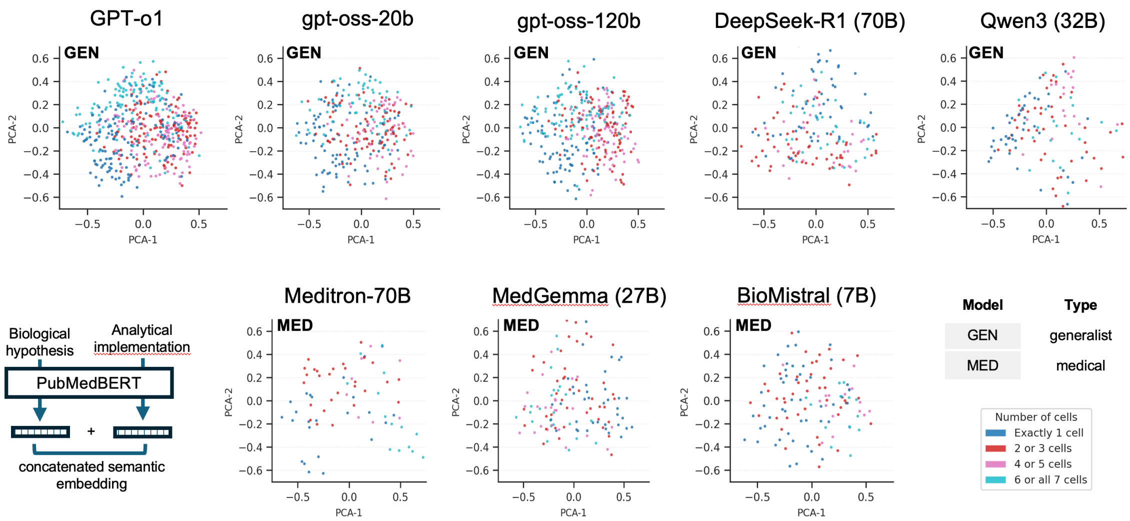


**Suppl. Figure 2**

Overall, we find that open-source models can serve as a viable alternative to commercial models.

<u>In conclusion, our results provide evidence that SPARK can be implemented with high performance using fully open-source, locally running models. However, the best results are achieved with the largest, most capable, generalist models. At this stage, medical domain models can be used but do not appear to be the models of choice,</u>

**primarily due to their smaller size, limited and clinically oriented pre-training, and largely absent reasoning capabilities.**

**We will provide detailed end-user guidelines in our GitHub repository accompanying these benchmarks for idea generation and coding.**

• Discuss how the system handles hallucinations during the idea generation phase

**Response:**

**Our pipeline includes a dedicated idea review stage that evaluates the generated ideas for potential violations of established guidelines, including instances of hallucination. This mechanism has proven highly effective, as we have not observed any cases exhibiting such issues. In general, when reviewing rejected ideas, we found that they were largely unaffected by hallucinations. Such problems typically arise when large language models are required to provide factual knowledge. In our case, the models are not asked to generate factual information but rather to manipulate data and objects based on common sense and general conceptual understanding.**

**We emphasize this in the manuscript (Page 15).**

• Clarify whether expert human validation is always required for newly generated concepts, and how this impacts the system's autonomy.

**Response:**

**Formally, human validation is not required at any intermediate stage. However, we examined the generated ideas and the corresponding Python functions out of interest and to gain a structured understanding of the framework's internal processes. In a typical use case, the analysis proceeds automatically until completion, and only if the generated ideas yield meaningful results with respect to the selected endpoints are they reviewed - for instance, to gain new biological insights or to better understand the mechanism of action of specific parameters, or to plan validation at another level.**

• Elaborate on how the performance of the entire system is assessed. Is there a need to evaluate each phase separately?

**Response:**

**Several key metrics can be analyzed after and during running the framework, as described in the manuscript. For the idea generation phase, these include the number of generated ideas added to database, duplicate ideas, and rejected ideas. For the coding phase, a relevant metric is the number of successfully implemented parameters.**

**Since state-of-the-art reasoning models are employed in both the idea generation and coding stages, it is generally unnecessary to evaluate the performance in single phases or intervene. However, based on the performance results of the open-source models discussed above, when smaller models are used, it may be necessary to closely monitor both stages to enable an early switch to more capable models if the initially selected ones underperform.**

• If an error is detected, is the system capable of identifying the specific module responsible?

**Response:**

**The agentic framework is stable and does not crash under normal operation. Within this framework, ideas for single-cell image analysis are generated and automatically transformed into Python functions capable of analyzing specific parameters.**

**Extensive logging is implemented across all stages of the framework. Most "real errors" naturally occur during the code-generation for parameters and execution phases, where both errors and execution times are recorded and used to iteratively improve the generated code (multiple cycles of code refinement are allowed, number of cycles is defined by human). When an error is detected, the logs enable precise identification of the specific module or stage responsible.**

**Additionally, guideline violations in the generated ideas are continuously monitored — unimplementable ideas are automatically rejected, and all related logs (including the rejected ideas) are preserved for later review.**

**While the framework has been carefully designed and optimized to handle diverse edge cases, adapting it to new task formulations or specialized use cases may require minor fine-tuning of the task descriptions after initial pipeline runs.**

• Please expand on the role of XGBoost in the system. As it appears to use both autonomously and human-generated parameters to predict biomarker status, its role is critical. Also, why was XGBoost chosen over other classifiers such as Random Forest or deep neural networks? A comparative discussion would add value.

**Response:**

**We provide additional details on the selection of XGBoost as the target approach for training predictive models. We do not apply it to human-generated parameters, as this was not our objective. In the human-generated component and the spatial biology use case, we only aim to demonstrate the exploratory capabilities of SPARK beyond its primary application (H&E pathology images and fully autonomous operation). Therefore, XGBoost is applied exclusively to the parameters and hypotheses generated in the main experiment - i.e., those derived through fully autonomous generation without human input.**

**Our decision to select XGBoost was based on its optimal balance of predictive performance during the prototyping phase, along with strong interpretability, robust feature and imbalances handling, and effective regularization.**

**During prototyping, we also evaluated TabPFN, a representative of a new class of deep learning algorithms for tabular data. Although its performance was comparable to that of XGBoost, we discontinued its use early due to explainability limitations. In our use case, SHAP-based explainability available in XGBoost provides a far more transparent and interpretable framework, whereas TabPFN remains largely a black box. Additionally, the relatively small dataset and limited number of parameters in our study were insufficient for TabPFN to achieve its typical state-of-the-art performance.**

**We also tested Random Forest and LightGBM, both of which demonstrated lower accuracy. Conceptually, XGBoost also relies on decision trees, but each tree is trained to correct the errors of previous ones while incorporating additional regularization. Furthermore, XGBoost handles imbalanced datasets more effectively - which aligns with our data characteristics - thereby explaining its superior accuracy.**

**Overall, the differences in performance across methods were relatively small (1–3%), suggesting that they capture essentially the same underlying signal, albeit with slightly different processing strategies. Hence, these approaches can be considered largely interchangeable in terms of their predictive capabilities on this dataset.**

**We have added several clarifying points regarding this choice to the Materials and Methods section.**

Reviewer #4 (Remarks on code availability):

I have checked the code. It is well documented and commented. It includes README file with enough instructions for installing and running the application. However, I did not run it.

**Response:**

**Thank you for reviewing the code. We will release the GitHub repository containing independently peer-verified code, detailed user instructions, optimal usage strategies, and benchmarking results for open-source models.**

Dear Editors and Reviewers,

Thank you for the positive assessment of our work. We have addressed all comments in the revised version of the manuscript.

Please find below our point-by-point responses. We hope you will now find the manuscript acceptable for publication.

As parts of the text were removed to simplify the manuscript and improve readability, we additionally include a Microsoft Word version with Track Changes enabled so that all modifications can be easily reviewed.

Please do not hesitate to contact me if you have any further questions.

Kind regards,
Yuri Tolkach, MD

***Editorial comments***

1) The introduction can be shortened significantly, while retaining the section on previous studies.

**Response:**
**The intoduction has been significantly shortened.**

2) In the results, following the overview of SPARK, the editorial team suggests to condense the sections related to Scenarios 1/2: from page 7 "Correlation to key pathological variables" to page 9 "Multiparametric prognostic system" by focusing on one or two easy-to-follow examples per scenario, which will also be included in the related main figure. The examples should include the rationale, details on the datasets used, more details on the ideas generated and the validation of some of the ideas generated. We feel that throughout the results the validation parts are very brief and more details should be added in the text on the data that were used to test and validate the ideas.

**Response:**
**We reformatted and streamlined the manuscript in line with these suggestions. We simplified the text and figures by adding clearer rationale, reducing the number of tumors/parameters/SHAP plots, and focusing the correlation and predictive analyses on fewer, more relevant tests. For the prognostic use case, we concentrate on lung adenocarcinoma. We also condensed the sections on multiparametric models to the essential results and removed most other tumor types.**

**In addition, we provide a concise validation and biological interpretation of the selected parameters in both the Figures and the text across all three parts**

**(correlation, predictive, and prognostic), again demonstrating their relevance and meaning. Thank you for this comment - showing what are the parameters and that they are biologically grounded is indeed crucial for readers. To support biological validity and the direction of our findings, we added citations to recent high-quality studies. For example, Aung et al. (Nature Genetics, 2025) from highly complex molecular integration analysis reports largely overlapping features for predicting immunotherapy outcomes in lung cancer with our top SHAP features for predicting PD-L1 status (simple H&E analysis with SPARK ideas), which we interpret more broadly as reflecting immune-evasion biology.**

**For the mentioned advanced zonal concept (Figure 3), we include a graphical abstract that explains the general principle and highlights this idea as it consistently showed significance in both the correlation and prognostic analyses.**

3) The Human-initiated idea generation could be expanded to provide some examples in the text of the ideas that were proposed, and also the text related to the independent validation should be expanded.

**Response:**
**We provide idea examples and expland independent validation description.**

4) We suggest to condense the section on the tracing of tumor evolution. The discussion should also be condensed, and the limitations of the model and the need for further validation should be highlighted.

**Response:**
**The section has been condensed to concetrate on high level, most important results with links to extended data and supplements for deeper insights if necessary. Discussion section has been significantly condensed. We believe that limitations section (including point mentioned) is very comprehensive.**

5) The figures need to be significantly simplified with a maximum of 8 to 10 panels per figure. We suggest the following: Figure 1 should contain a simplified version of panels c and d (the workflow) and f (a visual summary of the use cases) only. Figure 2 and 3 could be dedicated to case use 1 and 2, selecting only couple of tumor types and leaving more space to the visualisation of the ideas and parameters (for example some of the parameters in 3g are hard to understand like correlation_coeff or Zone4_std_perim). Similarly, figure 4 could contain the a selected examples for scenario 3. Figures 5 and 6 also should be simplified by selecting a couple of tumor types and reducig the number of plots.

**Response:**
**The Figures have been significantly simplified.**

**Figure 1 – panels a and b removed, panel c (Pipeline overview) – significantly simplified.**

**Panel c (in new Figure 1) is in out opinion crucial for understanding and actually very simple, we would prefer to leave it in the Figure, also given that for now, Figure 1 does not seem overloaded any more.**

**Figure 2 – significantly simplified in many aspects and significantly reduced in size and load.**

**Figure 3 – significantly simplified and most of the information moved to Extended Data Figures or Supplements.**

**Validation of parameters added as well as graphical abstract of one idea which proven to be significant for many types of analysis.**

**Figure 4 – significantly simplified, validation added. Concentration on LUAD as tumor type. Significantly simplified multi-parametric scoring system part.**

**Figure 5 - We do not believe further simplification is feasible or appropriate, as the figure already consists of straightforward, easy-to-follow elements and has been reduced to the minimum necessary content in the previous simplification round.**

**Figure 6 – shortened to essential parts, concentrates on LUAD in major parts, two panels moved to Extended data.**

6) The number of Supplementary tables is eccessive. We suggest to combine all of these in a single workbook.

**Response:**
**We substantially reduced the number of Supplementary Tables via merging – now 24 tables (previously: 57). We now provide a reference to all Supplementary Tables for easy navigation on the first page of the document.**

7) Please deposit the SPARK code into a suitably publicly accessible repository such as Zenodo and provide the link in the code availability at this stage. In the data Availability Statement please also include links to access the data that were used in the study.

**Response:**
**All relevant code, coded functions, reference books for parameters were deposited at Zenodo: https://zenodo.org/records/18047852**

**SPARK github repositorium has been available to public (https://github.com/cpath-ukk/SPARK).**

**Data availability section with correponding links has been added to Methods.**

8) In the methods provide additional details on the datasets used. Pleae note that there is no word limit for the methods, so some of the more technical details that are currently in the results section could also be moved into the methods.

**Response:**
**We substantially expanded information on the datasets used (summarizin information from Table S1).**

***Formatting issues***

In addition to addressing the remaining points from the editors and reviewers, please edit your manuscript to comply with our formatting guidelines for Articles, which are:

* Abstract: 200 words, unreferenced.

**Response: Confirmed.**

* Main text: 4000 words with subheadings for the Introduction, Results and Discussion

**Response: The main text size was reduced to cope with this limit during implementing the recommendation to shorten and simplify Introduction, Results and Discussion sections.**

* References: up to 60 in the main text + 20 methods-only references

**Response: Confirmed.**

* Display items: up to 6 main display items (inclusive of figures and tables) and up to 10 Extended Data display items (inclusive of figures and tables). Extended Data are an integral part of the paper and only data that directly contribute to the main message should be presented.

**Response: Confirmed (One extended Figure was moved to Suppl. Figures, resulging in 9 extended Figures).**

* Online Methods: no word limit; please provide the methods consolidated in a single section at the end of the main text document

**Response: Confirmed.**

***Reviewers' Comments:***

Reviewer #2 (Remarks to the Author):

The authors have satisfactorily addressed most of my main concerns. In particular, I appreciate the new comparison between generalist models and dedicated medical models, as well as the authors’ rationale for the observed inferiority of the latter. Given the broad and diverse set of experiments, some increase in complexity of the

manuscript is understandable. I encourage the authors to make full use of the Supplementary Information to present methodological details and additional analyses so that the main text remains clear and focused while retaining academic rigor.

**Response: Thank you. Also following the recommendation of Editors, we significantly simplified the main Figures and manuscript text.**

Reviewer #3 (Remarks to the Author):

General Comments First, I thank the authors for their comprehensive response. As a clinical reviewer, I cannot verify the accuracy of the purely technical adjustments; however, I appreciate the thoroughness of the revisions. My previous concerns are mostly alleviated, though I have a few remaining observations:

A. Clarity and Readability The text is significantly improved. I believe my experience reflects that of the average reader, and I could follow the narrative much better this time. The added definitions and the revisions to the text (and black text updates) are helpful. Regarding abbreviations in the figures: while I appreciate the effort, the abbreviations within the figures are somewhat obscured by the sheer volume of subfigures. I appreciate the authors' diligence in addressing clarity, though I remain slightly cautious that my improved understanding might be partly due to my previous deep-dive into the material rather than the text alone. I hope the editors are aware of this limitation in my revision.

B. Formatting Errors: I cannot fully evaluate the manuscript due to rendering errors in the PDF. Page 15 appears to be cut off/missing half the content, and the reference list seems to suffer from the same issue.

C. Organization of Supplementary Data: With 57 Excel sheets and a supplementary PDF containing 20 complex figures, the data is difficult to navigate. I strongly recommend adding a dedicated Table of Contents as the first sheet in the Excel file and the first page of the supplementary PDF.

D. Final Recommendation: The volume of data remains overwhelming, though I acknowledge this may be unavoidable given the scope of the work. I have not read all 57 tables nor all the subfigures in the supplementary - and neither will the average viewer. That's probably okay (as I suppose it is for those interested), but it is very hard to navigate in its current form, which makes it inaccesible, and there's too much to delve into the data whilst you read the article the first time.
I appreciate the data in the main manuscript has been drastically reduced.

Assuming the technical reviewers are satisfied with the system's validation, I recommend publication. Whether this fits Nature Medicine or a domain-specific journal is up to the editors, but I believe the quality and scope are worthy of Nature Medicine. The work is impressive, and its open-source nature makes it a valuable tool. I also see significant potential for expanding this approach to other domains (e.g., radiology, biochemistry) if upstream tools allow. A major differentiator between this work and

other AI manuscripts I have reviewed is the robust utilization of agentic AI. Of course - this is all hinging on the evaluation of more technical reviewers.

Thank you to both Nature and the authors.

**Response: Thank you for your positive evaluation. The complexity of the main Figures was reduced, the number of supplementary Tables was reduced from 57 to 24 and the reference was added in the beginning of the document. The text of the manuscript was also simplified to aid understanding.**

*Reviewer #4 (Remarks to the Author):*

The authors have addressed my concerns in the revised manuscript, and I recommend the publication of this work.

**Response: Thank you.**
